# SPHK1-mediated M2 macrophage polarization drives TGF-β1-dependent thrombus fibrosis

**DOI:** 10.3389/fimmu.2025.1681485

**Published:** 2025-11-17

**Authors:** Xiaoyun Chen, Fajiu Li, Guofeng Ma, Haifeng Qiang, Maohe Chen, Shi Chen, Yedong Huang, Xingyue Lai, Qinghuang Lin, Chaosheng Deng

**Affiliations:** 1 The School of Clinical Medicine, Fujian Medical University, Department of Respiratory and Critical Care Medicine, Fujian Provincial Geriatric Hospital, Fuzhou, China; 2 Department of Pulmonary and Critical Care Medicine, The Sixth Hospital of Wuhan, Affiliated Hospital of Jianghan University, Wuhan, China; 3 Department of Pulmonary and Critical Care Medicine, Regional Medical Center for National Institute of Respiratory Diseases, School of Medicine, Sir Run Run Shaw Hospital, Zhejiang University, Hangzhou, China; 4 Department of Cardiac Surgery, Xiamen University Affiliated Cardiovascular Hospital, Xiamen, China; 5 Department of Radiation Oncology, Clinical Oncology School of Fujian Medical University, Fujian Cancer Hospital, Fuzhou, China

**Keywords:** SPHK1, venous thrombosis, fibrosis, M2 macrophages, TGF-β1, single-cell sequencing

## Abstract

**Background and objective:**

Venous thrombus fibrosis contributes to post-thrombotic syndrome (PTS) and chronic thromboembolic pulmonary hypertension (CTEPH). M2 macrophages promote fibrosis via TGF-β1 secretion. This study investigates whether sphingosine kinase 1 (SPHK1) promotes thrombus fibrosis by regulating M2 macrophage polarization.

**Methods:**

Histological staining and immunofluorescence (IF) were performed on thrombus tissues from patients with acute thrombosis and CTEPH. Single-cell RNA sequencing (scRNA-seq) was used to characterize immune cell heterogeneity and to identify SPHK1 expression within macrophage subsets. *In vivo*, a rat model of thrombus was established via inferior vena cava (IVC) ligation, and the SPHK1 inhibitor PF543 was administered to evaluate its effects on fibrosis and macrophage polarization. *In vitro*, bone marrow-derived macrophages (BMDMs) were subjected to M2 polarization and co-cultured with fibroblasts to assess the TGF-β1-dependent fibroblast activation.

**Results:**

Histological analysis revealed significantly increased ECM deposition and macrophage infiltration in CTEPH thrombi compared to acute thrombi. Masson staining demonstrated extensive collagen fiber accumulation in CTEPH samples. Immunofluorescence analysis of fibrotic thrombi from a rat inferior vena cava (IVC) ligation model showed strong co-expression of SPHK1 and CD68, indicating the presence of SPHK1-expressing macrophages in thrombus remodeling. scRNA-seq analysis further revealed high SPHK1 expression in M2 macrophage subsets, particularly in the MARCO-1 cluster, and its expression was closely correlated with TGF-β1 secretion. *In vivo*, PF543 treatment significantly reduced collagen deposition, TGF-β1 expression, and M2 macrophage polarization in thrombus tissue. *In vitro*, SPHK1 knockdown markedly suppressed the expression of TGF-β1, Arg1, CD36, and FASN in BMDMs, indicating an inhibition of pro-fibrotic macrophage function. Co-culture experiments further confirmed that M2 macrophages activated fibroblasts via a TGF-β1-dependent mechanism.

**Conclusion:**

This study demonstrates that SPHK1 promotes M2 macrophage polarization and drives TGF-β1-dependent thrombus fibrosis, underscoring its critical role in the progression of CTEPH. Pharmacological inhibition of SPHK1 by PF543 effectively attenuates fibrotic remodeling and suppresses M2 macrophage polarization, suggesting that SPHK1 may serve as a promising therapeutic target for the treatment of chronic thrombus-associated fibrosis.

## Introduction

Venous thromboembolism (VTE), comprising deep vein thrombosis (DVT) and pulmonary embolism (PE), is a major global cause of morbidity and mortality (1). Despite adequate anticoagulation using agents such as low-molecular-weight heparin, vitamin K antagonists (e.g., warfarin), and direct oral anticoagulants (DOACs), up to 20–50% of DVT patients develop post-thrombotic syndrome (PTS), characterized by chronic thrombus fibrosis, pain, swelling, and ulceration (2). Similarly, 25–50% of PE patients develop persistent thrombi that undergo fibrotic remodeling of the pulmonary vasculature, progressing to chronic thromboembolic pulmonary disease (CTEPD), and in some cases, to chronic thromboembolic pulmonary hypertension (CTEPH) (3). CTEPH is a serious and potentially fatal condition with a 3-year survival rate as low as 10–20% without treatment (4). Importantly, a subset of patients develops CTEPH despite being on adequate anticoagulation, suggesting that non-thrombotic mechanisms such as pathological fibrosis play a critical role in disease progression.

Chronic thrombus fibrosis contributes to vascular occlusion and impaired thrombus resolution. Fibroblast activation and excessive extracellular matrix (ECM) deposition, driven by immune cell infiltration, are hallmarks of this process. Single-cell RNA sequencing (scRNA-seq) analyses of pulmonary endarterectomy (PEA) samples from CTEPH patients have revealed substantial infiltration of M2-polarized macrophages, fibroblasts, and α-SMA^+^ myofibroblasts, highlighting the immune-fibrotic nature of thrombus remodeling in CTEPH (5, 6). Among immune cells, macrophages are particularly important due to their plasticity in response to environmental cues. M2-like macrophages, induced by IL-4 and IL-13, are associated with tissue repair and fibrosis (7). These cells secrete profibrotic cytokines such as transforming growth factor-β1 (TGF-β1) and IL-10, which activate fibroblasts and promote ECM accumulation (8).

Recent studies have revealed a strong link between M2 macrophage polarization and intracellular lipid metabolism, which supports their anti-inflammatory and pro-reparative functions (9). Dysregulation of lipid metabolism has been shown to modulate M2 polarization in various fibrotic disease models (10–12). In thrombotic tissues from CTEPH patients, TGF-β1 expression is markedly elevated, suggesting sustained local activation of M2 macrophages (13). As a key profibrotic cytokine, TGF-β1 further amplifies M2 polarization and fibroblast activation, promoting ECM deposition and fibrotic remodeling (14).

Sphingosine kinase 1 (SPHK1), a rate-limiting enzyme in sphingolipid metabolism, has been shown to regulate inflammatory activation and fibrosis in various diseases, including spinal cord injury and liver fibrosis, via pathways involving S1P/S1PR3/p38 MAPK and CCL2-CCR2 (15–18). However, its role in thrombus fibrosis remains unexplored. In this study, we investigated the functional role of SPHK1 in thrombus fibrosis through analysis of human thrombus samples, single-cell transcriptomics, *in vitro* macrophage–fibroblast co-culture systems, and pharmacological inhibition in a rat model. We found that SPHK1 is highly expressed in M2 macrophages and promotes fibroblast activation via TGF-β1 secretion, thereby driving thrombus fibrosis. These findings provide new mechanistic insight into the development of chronic thrombotic disease and suggest that SPHK1 may represent a promising antifibrotic therapeutic target.

## Materials and methods

### Human thrombus sample collection and processing

Acute thrombotic tissues were obtained from six patients with pulmonary embolism (PE) who underwent catheter-directed thrombectomy at the Department of Interventional Radiology, First Affiliated Hospital of Fujian Medical University. Immediately after retrieval, the samples were preserved in a commercial tissue stabilization solution and transported via cold chain to Singleron Biotechnologies (Nanjing, China) for single-cell suspension preparation and downstream RNA sequencing. The thrombi were classified as acute based on the clinical onset (<14 days), intraoperative appearance (dark red, soft, non-recanalized), and absence of fibrous remodeling.

Chronic thrombotic tissues were derived from publicly available single-cell RNA sequencing (scRNA-seq) data of pulmonary endarterectomy (PEA) specimens from patients with chronic thromboembolic pulmonary hypertension (CTEPH), downloaded from the GEO database (accession number: GSE224143). Among the five original samples, GSM7016384 and GSM7016387 were excluded due to poor quality and low cell count. The remaining three samples, each with >2,000 high-quality cells, were included for integrated single-cell analysis. The diagnosis of CTEPH in these patients was based on right heart catheterization and computed tomography pulmonary angiography in accordance with the 2022 ESC/ERS guidelines.

For validation experiments, both acute thrombi and PEA tissues were additionally collected and processed for histological and molecular studies. All specimens were handled within 30 minutes of excision. Tissues were either fixed in 4% paraformaldehyde for paraffin embedding and histological analysis or snap-frozen in liquid nitrogen for RNA and protein extraction. Gross and microscopic evaluations were performed to ensure accurate sampling of thrombotic material without contamination from adjacent vascular structures.

### Animal model and thrombus tissue collection

Sprague-Dawley (SD) rats (200–250 g, 8–10 weeks old) were obtained from the Laboratory Animal Center for Medical Sciences and housed in a specific pathogen-free (SPF) facility with ad libitum access to food and water. Rats were randomly assigned to the model group or treatment group. Venous thrombus formation was induced using a partial ligation method of the inferior vena cava (IVC), performed under sterile conditions and general anesthesia. The IVC was exposed through a midline laparotomy, and a ligature was placed approximately 1 cm below the renal veins to restrict blood flow without completely occluding the vessel. After surgery, rats were allowed to recover in a temperature-controlled environment. Treatment began 24 hours after ligation. Rats in the treatment group received the SPHK1 inhibitor PF543 (5 mg/kg, intraperitoneally, once daily) for 7 or 14 days. Control rats were administered an equal volume of normal saline.

At the indicated time points, rats were euthanized by intraperitoneal injection of sodium pentobarbital (150 mg/kg body weight), in accordance with the American Veterinary Medical Association (AVMA) Guidelines for the Euthanasia of Animals (2020). Following euthanasia, the IVC segments containing thrombi were carefully dissected. Surrounding connective tissue and fat were meticulously removed under a stereomicroscope. The vein was longitudinally incised, and the thrombus core was gently separated from the vascular wall using fine forceps. Only the thrombus tissue itself—excluding any adherent vascular or perivascular structures—was collected for downstream histological, molecular, and biochemical analyses. Care was taken to ensure consistent sampling and minimize contamination from non-thrombotic tissue. Throughout the experiment, animals were monitored daily for changes in body weight, food intake, and general condition to assess any adverse effects of treatment.

### Single-cell RNA sequencing and data analysis

Thrombus tissues were collected from patients with CTEPH and acute pulmonary embolism for single-cell suspension preparation, followed by library construction and sequencing using the 10x Genomics platform. Raw sequencing data were processed using the CeleScope pipeline (v1.9.0, Singleron), which includes demultiplexing, alignment to the GRCh38 reference genome, barcode counting, and unique molecular identifier (UMI) quantification. Downstream data analysis, including quality control, dimensionality reduction, and clustering, was performed using Scanpy (v1.9.3) in the Python (v3.7) environment. For each sample, genes expressed in fewer than 5 cells, and cells expressing fewer than 200 genes or having mitochondrial gene content exceeding 10%, were excluded from further analysis. Batch effects across subgroups were assessed and removed using Harmony (version 0.0.6). Cell clustering was performed based on the CellID algorithm (version 0.1.0), with the resolution parameter set to 1.2, resulting in 22 distinct clusters. Marker gene analysis for each cluster was conducted using the “FindAllMarkers” function with default parameters. The clustering results were visualized using t-distributed stochastic neighbor embedding (t-SNE). Intercellular ligand–receptor interactions were analyzed using the CellChat framework.

### Reclustering of macrophages and monocytes

Macrophage and monocyte populations were extracted from the initial clustering results for myeloid-specific reclustering. Using a resolution parameter of 1.2, the extracted cells were reclustered, yielding six distinct subclusters.

### Pseudotime trajectory reconstruction

Cellular differentiation trajectories were reconstructed using Monocle2 (v2.22.0). Cells were first ordered based on highly variable genes (HVGs) to reflect their spatial and temporal progression during differentiation. Feature extraction and dimensionality reduction were performed using the DDRTree algorithm and the FindVariableFeatures function. The resulting trajectories were visualized using the plot_cell_trajectory function.

### Hematoxylin–eosin and Masson’s trichrome staining

Thrombus tissues were fixed in 4% paraformaldehyde for 24 hours, followed by paraffin embedding and sectioning into 5-μm-thick slices. For HE staining, the sections were deparaffinized in xylene, rehydrated through a graded ethanol series, and stained with hematoxylin and eosin. Nuclei appeared blue-purple, while cytoplasm was stained pink, allowing clear visualization of tissue architecture. To assess collagen deposition, Masson’s trichrome staining was performed using a commercial kit (Solarbio, China). In the stained sections, collagen fibers appeared blue, muscle fibers and cytoplasm red, and nuclei dark blue. For each sample, five high-power fields were randomly selected under a light microscope for image capture. Fibrotic area and cell density were quantified using ImageJ software, and differences among treatment groups were compared. Whole-slide scanning of stained sections was performed using the Panoramic SCAN II scanner (3D HISTECH, Hungary).

### Immunofluorescence staining

Paraffin-embedded tissue sections were first dewaxed in an environmentally friendly dewaxing agent for 30 minutes, followed by sequential rehydration in absolute ethanol, 95% ethanol, and 75% ethanol for 5 minutes each. Sections were then rinsed three times with distilled water to complete hydration. Antigen retrieval was performed according to experimental requirements using different methods, including high-temperature and high-pressure retrieval with EDTA buffer (pH 9.0, 1 minute 30 seconds), citrate buffer (pH 6.0, 2 minutes), or microwave retrieval by heating at high power for 3 minutes per cycle for three cycles, with cooling to room temperature between cycles. After antigen retrieval, tissue sections were blocked with 10% normal serum at 37°C for 30 minutes to prevent nonspecific antibody binding. Excess serum was removed, and primary antibodies (50–100 μL) were applied at appropriate dilutions and incubated overnight at 4°C. The following day, sections were brought back to room temperature and washed three times with TBST buffer. Secondary antibodies (50–100 μL) were then added and incubated at 37°C for 30 minutes. After incubation, nuclei were counterstained with DAPI for 5 minutes in the dark, followed by three TBST washes. Sections were mounted using antifade fluorescence mounting medium and stored at 4°C in the dark. Stained sections were examined and imaged using a fluorescence microscope. Positive staining areas in high-power fields were quantified using ImageJ software. The primary antibodies used for immunofluorescence included CD206 (rabbit, 1:500, 24595, Cst), CD68 (rabbit, 1:4000, Ab303565, Abcam), iNOS (rabbit, 1:250, ab283655, Abcam), SphK1 (rabbit, 1:200, 10670-1-AP, Proteintech), and α-SMA (rabbit, 1:2000, ab124964, Abcam). For immunohistochemistry, TGF-β1 (rabbit, 1:400, 21898-1-AP, Proteintech) was used.

### RNA sequencing analysis

Total RNA was extracted using TRIzol reagent (Invitrogen), and RNA quality was assessed using the 2100 Expert Bioanalyzer (Agilent). RNA sequencing was performed on the Illumina HiSeq 2000 platform at Majorbio Biotech (Shanghai, China). Online data analysis was conducted via the I-Sanger Cloud Platform. Differential expression analysis between the two groups was performed using the DESeq2 package. Genes or transcripts with a false discovery rate (FDR) < 0.05 and an absolute fold change ≥ 2 were defined as differentially expressed genes (DEGs) or transcripts.

### Gene set enrichment analysis

Gene set enrichment analysis was carried out on the I-Sanger online platform. The gene expression matrix was uploaded, and genes were ranked based on log_2_ fold change values between the experimental and control groups. KEGG (Kyoto Encyclopedia of Genes and Genomes) gene sets were used as the reference database for enrichment analysis. Significant enrichment was defined by the following thresholds: normalized enrichment score (NES) > 1, FDR < 0.05, and P-value < 0.05. The I-Sanger platform automatically generated enrichment plots and detailed statistical reports, providing a list of significantly enriched gene sets and their associated biological pathways.

### Reactome pathway enrichment analysis

Reactome pathway enrichment analysis was also conducted on the I-Sanger platform. DEGs were uploaded and analyzed using the Reactome Pathway Database. The significance of pathway enrichment was assessed using a hypergeometric test, and pathways with a P-value < 0.05 were considered significantly enriched. Enrichment results were visualized using bubble plots, which illustrated the biological functions and interactions of DEGs within enriched signaling pathways.

### Isolation of rat bone marrow-derived macrophages and induction of M2 polarization

Rat bone marrow-derived macrophages (BMDMs) were isolated following a standard protocol. Bone marrow was harvested from the femurs and tibias of 8-week-old Sprague-Dawley rats. The ends of the bones were cut, and the marrow cavities were flushed with sterile phosphate-buffered saline (PBS) to collect bone marrow cells into sterile centrifuge tubes. The cell suspension was filtered through a 70 µm cell strainer to remove bone fragments and debris, followed by centrifugation at 300 × g for 5 minutes. The supernatant was discarded, and the cell pellet was resuspended in Dulbecco’s Modified Eagle Medium (DMEM) supplemented with 10% fetal bovine serum (FBS) and 1% penicillin-streptomycin. Cells were seeded into sterile culture dishes and incubated at 37°C in a humidified atmosphere with 5% CO_2_. To promote macrophage differentiation, 20 ng/mL macrophage colony-stimulating factor (C470, Novoprotein) was added. The medium was replaced every 2 days with fresh medium containing M-CSF. After 7 days of induction, adherent cells were assessed by flow cytometry for dual expression of CD11b and CD68 to confirm macrophage identity. For M2 polarization, mature BMDMs were washed with PBS and cultured in serum-free medium supplemented with 20 ng/mL interleukin-4 (400-04, PeproTech). Cells were maintained for an additional 48 hours under standard conditions (37°C, 5% CO_2_). Following induction, M2-polarized macrophages were harvested for functional assays and further experimental analyses.

### Isolation and culture of fibroblasts from thrombus tissue

Thrombus tissue was harvested from the inferior vena cava ligation model in rats and immediately rinsed with PBS containing antibiotics to remove residual blood and minimize contamination. The tissue was transferred to a sterile culture dish and finely minced using sterile scissors to maximize the surface area for enzymatic digestion. Tissue digestion was performed using the Tissue Dissociation Kit (Miltenyi Biotec, Germany) following the manufacturer’s instructions. The minced tissue was transferred into C-tubes containing 4.7 mL of pre-warmed serum-free medium (SFM) to optimize enzymatic activity. Mechanical dissociation was conducted using the GentleMACS Dissociator (Miltenyi Biotec, Germany), and C-tubes were incubated in a 37°C water bath for 30 minutes. This cycle of mechanical dissociation and enzymatic incubation was repeated twice to ensure efficient tissue dissociation. After the final mechanical step, enzymatic digestion was terminated by adding 20 mL of PBS. The resulting cell suspension was passed through a 70 µm cell strainer to remove undigested tissue fragments. Filtered cells were centrifuged at 300 × g for 5 minutes, and the supernatant was discarded. The cell pellet was resuspended in DMEM supplemented with 10% FBS and 1% penicillin-streptomycin, and then plated into culture dishes. Cells were cultured at 37°C in a humidified atmosphere with 5% CO_2_. After 24 hours, the culture medium was replaced to remove non-adherent cells, and fresh medium was added every 2–3 days thereafter. To selectively enrich fibroblasts, a differential adhesion method was employed, taking advantage of the rapid adherence properties of fibroblasts. This approach allowed gradual enrichment and expansion of fibroblasts during medium changes. Confluent monolayers of adherent cells were typically observed within 7–10 days, at which point cells were passaged for subsequent experiments.

### Co-culture system

In the conditioned medium (CM) co-culture system, M2-polarized macrophages were first cultured alone for 48 hours. The supernatant was then collected and filtered through a 0.22 µm membrane to obtain M2-conditioned medium (M2-CM), which was stored at 4°C until use. Fibroblasts were seeded into 6-well plates at a density of 1 × 10^5^ cells/well and cultured for 48 hours with different types of conditioned media (see **Table 1**). Morphological changes in fibroblasts under each condition were observed using a light microscope. In addition, immunofluorescence staining and Western blot analysis were performed to evaluate the expression of the myofibroblast marker α-smooth muscle actin (α-SMA).

**Table 1:** Treatment and cells in 4 groups of supernatant-cell coculture systems.

Groups	SB525334	M2 CM	FBs
Control	–	–	^+^
M2 CM	–	^+^	^+^
M2 CM^+^SB525334	^+^	^+^	^+^
SB525334	^+^	–	^+^

Abbreviations: CM=conditioned medium; FBs=fibroblasts.

### Western blot analysis

Total protein was extracted from cells and tissues and separated by sodium dodecyl sulfate–polyacrylamide gel electrophoresis (SDS-PAGE), followed by transfer to a polyvinylidene fluoride (PVDF) membrane (Millipore). The membrane was blocked with 10% non-fat milk and incubated sequentially with primary and secondary antibodies. Protein bands were visualized using an enhanced chemiluminescence (ECL) substrate (MA0186-1, Meilunbio) and detected with a chemiluminescence imaging system (SCG-W3000, Saivill). The following primary antibodies were used: SPHK1 (rabbit, 1:5000, 10670-1-AP, Proteintech), CD68 (rabbit, 1:1000, ERP23917-164, Abcam), CD206 (rabbit, 1:1000, 24595, CST), iNOS (rabbit, 1:1000, EPR16635, Abcam), Arginase-1 (rabbit, 1:1000, 93668, CST), TGF-β1 (rabbit, 1:1000, EPR21143, Abcam), α-SMA (rabbit, 1:50000, ET1607-53, Huabio), MMP9 (rabbit, 1:1000, RM1020, Abcam), Phospho-STAT3 (Tyr705) (rabbit, 1:1000, 9145, CST), and STAT3 (rabbit, 1:1000, 12640, CST). HRP-conjugated secondary antibodies and ECL were used for detection. β-actin was used as the internal loading control.

### Quantitative real-time PCR

Quantitative real-time PCR (qRT-PCR) was performed using the SYBR Green system, and data were analyzed using the 2^–ΔΔCt method. Total RNA was extracted from cells using a Fast RNA Extraction Kit (AG21023), followed by reverse transcription into complementary DNA (cDNA) using the Evo M-MLV RT Premix Kit (AG11728). qRT-PCR was conducted using the SYBR^®^ Green Pro Taq HS Premix qPCR Kit (AG11718). Primer sequences used for amplification are listed in **Table 2**. Gene expression levels were normalized to β-actin as the internal control.

**Table 2:** Primer sequences used for amplification.

Gene symbol	Forward 5’-3’	Reverse 5’-3’
β-actin	CGCGAGTACAACCTTCTTGC	CCTTCTGACCCATACCCACC
CD36	TCAACATACTGGTCAAGCCAGC	GACCATCTCAACCAGGCCCAG
FASN	GAAGCCTAGCTGCACCATCA	AGGGTTGATGTCGATGCCTG

### Flow cytometry

Single-cell suspensions were prepared from thrombus tissues via collagenase digestion. After Fc receptor blocking, cells were stained with the following antibodies: CD11b-FITC (201805, Biolegend), CD68-APC (bs-20403R, Bioss), CD163-RY610 (759119, BD OptiBuild), and 7-AAD (AP104, MultiScience) for viability exclusion. Data were acquired using a CytoFLEX LX flow cytometer and analyzed with FlowJo software to determine the proportion of CD68^+^CD11b^+^CD163^+^ M2-polarized macrophages.

### Enzyme-linked immunosorbent assay

The concentration of TGF-β1 in the culture supernatants of fibroblasts was measured using a commercial ELISA kit (Elabscience, E-EL-0162) following the manufacturer’s instructions. Briefly, collected supernatants were added to a microplate pre-coated with a rat TGF-β1-specific antibody, incubated with detection reagents, and the absorbance was measured at 450 nm using a microplate reader. TGF-β1 concentrations were calculated based on a standard curve. All samples were assayed in duplicate.

### Statistical analysis

Data are presented as mean ± standard deviation (SD). Statistical differences between two groups were assessed using an unpaired Student’s t-test, while comparisons among multiple groups were performed using two-way analysis of variance (ANOVA) followed by Bonferroni *post hoc* correction. A P-value < 0.05 was considered statistically significant. Data visualization and statistical analysis were performed using GraphPad Prism 9.0 and R software.

## Results

### CTEPH thrombi exhibit enhanced immune cell infiltration, fibrotic remodeling, and increased SPHK1 expression

**Figure 1:**
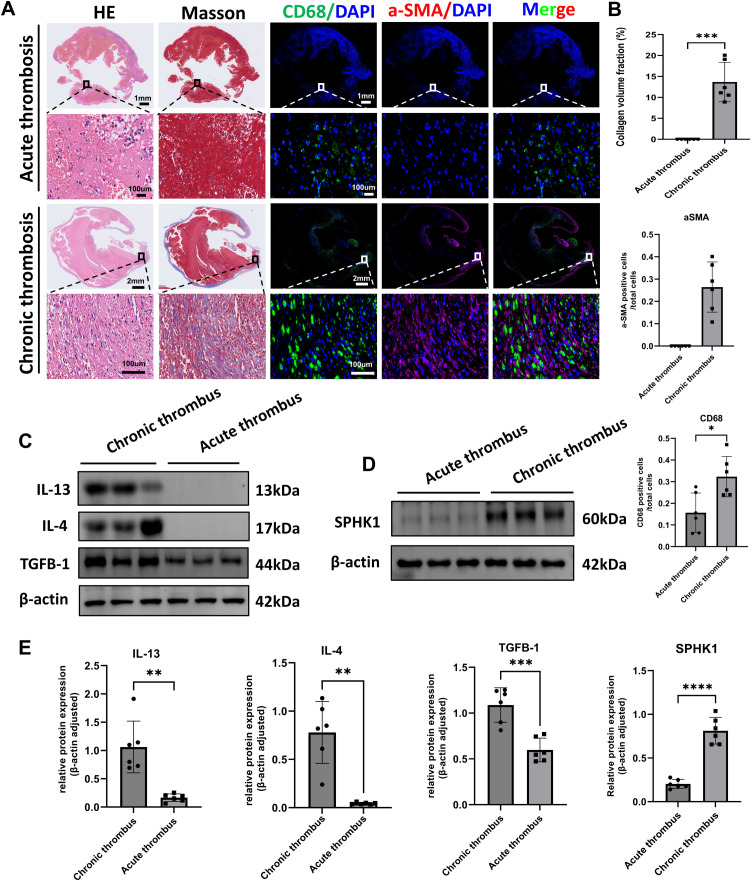
Histological and molecular characterization of acute and chronic thrombi in human samples. **(A)** Representative histological and immunofluorescence staining of thrombus sections from patients with acute pulmonary embolism and CTEPH (n = 6 per group). Panels include H&E staining, Masson’s trichrome staining (collagen in blue), immunofluorescence for CD68 (green, macrophages), α-SMA (red, smooth muscle actin–positive cells), and merged images. Nuclei were counterstained with DAPI (blue). **(B)** Quantification of fibrotic area (Masson’s), CD68^+^ macrophage count, and α-SMA^+^ cell count per high-power field. Data are presented as mean ± SD (n = 6 per group). *P < 0.05 by unpaired t-test. **(C)** Western blot analysis of IL-4, IL-13, and TGF-β1 protein expression in acute thrombi and CTEPH thrombi (n = 6 per group). β-actin was used as a loading control. **(D)** Western blot analysis of SPHK1 protein expression in the same samples as in **(C)**. **(E)** Densitometric quantification of IL-4, IL-13, TGF-β1, and SPHK1 expression normalized to β-actin. Data are presented as mean ± SD (n = 6 per group). *P<0.05, **P<0.01, ***P<0.001, ****P<0.0001.

To investigate the pathological differences between acute and chronic thrombi, we performed histological and molecular analyses on thrombus specimens from patients with acute pulmonary embolism (n = 6) and CTEPH (n = 6). H&E staining revealed that acute thrombi primarily consisted of erythrocytes and loosely organized inflammatory cells, whereas CTEPH thrombi exhibited dense cellular infiltration and organized tissue structure (**Figure 1A**). Masson’s trichrome staining showed substantial collagen deposition in CTEPH thrombi, indicative of advanced fibrosis. Immunofluorescence analysis further demonstrated a marked increase in CD68^+^ macrophage infiltration and α-SMA^+^ spindle-shaped cells—representing activated fibroblasts or myofibroblasts—in CTEPH specimens compared to acute thrombi (**Figures 1A, B**).

To explore underlying molecular changes, we assessed the protein expression of key pro-fibrotic and Th2-type inflammatory cytokines. Western blot analysis showed that levels of IL-4, IL-13, and TGF-β1 were significantly elevated in CTEPH thrombi relative to acute thrombi (**Figures 1C, E**). In parallel, we found that the expression of SPHK1 was also markedly upregulated in CTEPH samples (**Figures 1D, E**). These findings suggest that chronic thrombi in CTEPH are characterized by sustained inflammation, immune cell recruitment, fibroblast activation, and SPHK1-associated fibrotic signaling.

### Single-cell RNA sequencing reveals elevated SPHK1 expression associated with M2 macrophage subpopulations in CTEPH thrombi

**Figure 2:**
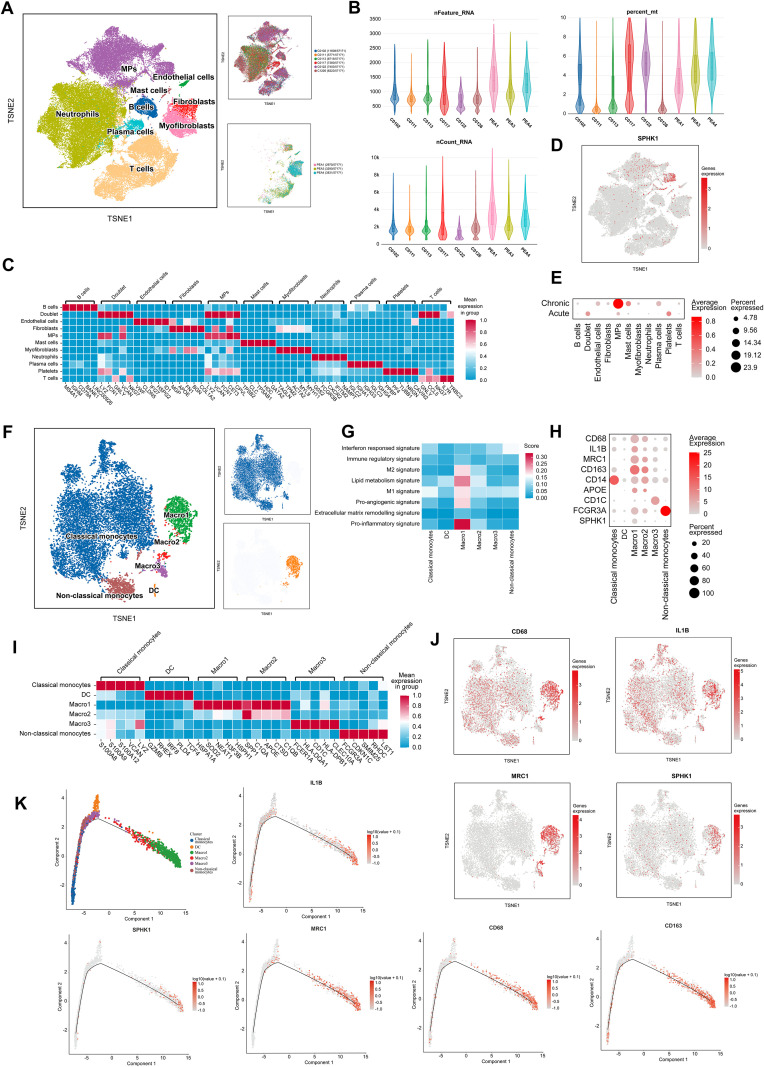
SPHK1-associated macrophage heterogeneity in thrombi revealed by single-cell analysis. **(A)** t-distributed Stochastic Neighbor Embedding (tSNE) analysis of cells isolated from thrombi of APE and CTEPH patients. **(B)** Sequencing depth, read count, and the percentage of reads mapped to mitochondrial genes across 9 single-cell samples. **(C)** Heatmap of marker genes for different cell populations, color-coded by cluster. Rows represent marker genes, and columns represent individual cells. **(D)** tSNE plot of 22 clusters colored by SPHK1 expression. Cluster coloring is based on normalized gene expression values. **(E)** Bubble plot representing SPHK1 expression levels in each cell population in both PE and CTEPH groups. **(F)** tSNE plot showing different monocyte/macrophage subpopulations, including classical monocytes, dendritic cells (DC), MARCO-1, MARCO-2, MARCO-3, and non-classical monocytes. **(G)** Macrophage-related gene set scoring shows that the Macro1 subset has significantly higher scores in M2 signature, M1 signature, lipid metabolism, pro-inflammatory, and pro-angiogenic categories compared to other subsets. **(H)** Bubble plot showing the expression levels of macrophage markers and M1/M2 polarization markers. **(I)** Heatmap of differentially expressed genes in monocyte and macrophage clusters, color-coded by cluster. Rows represent marker genes, and columns represent individual cells. **(J)** tSNE plots of 6 clusters, color-coded by normalized expression levels of CD68, IL-1B, MRC1, and SPHK1. **(K)** Pseudotime ordering of monocyte/macrophage clusters in a two-dimensional state space. The left side represents the origin of development, and the right side represents the terminal state. Trajectories of five representative genes (CD68, CD163, IL-1B, MRC1, and SPHK1) are shown.

To investigate cellular heterogeneity and gene expression profiles in thrombi at different disease stages, we performed single-cell RNA sequencing (scRNA-seq) on thrombus tissues from 3 patients with CTEPH and 6 patients with acute pulmonary embolism (APE). Detailed clinical information for all patients is provided in **Supplementary Table S1**. t-distributed stochastic neighbor embedding (tSNE) analysis identified multiple well-defined cell clusters (**Figure 2A**). Quality control metrics confirmed that all samples exhibited sufficient sequencing depth, a high number of detected genes, and appropriate mitochondrial gene proportions, indicating high data quality (**Figure 2B**). Based on canonical marker genes, major cell populations were annotated, including monocytes/macrophages, T cells, neutrophils, B cells, endothelial cells, and fibroblasts (**Figure 2C**).

We next examined SPHK1 expression across clusters. Notably, SPHK1 was highly enriched in the monocyte/macrophage cluster (**Figure 2D**). Comparative analysis revealed significantly higher SPHK1 expression in CTEPH samples than in APE samples across multiple cell types, with the most pronounced increase observed in monocytes/macrophages (**Figure 2E**). These findings suggest that SPHK1 may play cell type–specific regulatory roles in the chronic remodeling of CTEPH thrombi.

To further explore macrophage heterogeneity and its association with SPHK1 in CTEPH thrombi, we performed subclustering of the monocyte/macrophage population. Reclustering based on tSNE identified six distinct subpopulations: classical monocytes, non-classical monocytes, dendritic cells (DCs), and three MARCO^+^ macrophage subsets (MARCO-1, MARCO-2, MARCO-3) (**Figure 2F**). Gene signature analysis revealed that MARCO-1 macrophages expressed high levels of M1 and M2 polarization markers, genes related to lipid metabolism, inflammation, and angiogenesis (**Figure 2G**), suggesting a functionally active “mixed polarization” phenotype involved in both immune regulation and tissue remodeling. Pseudo-time analysis shows that the MARCO-1 subpopulation is located in the later stages of the differentiation trajectory, suggesting that these cells may represent a mature subpopulation rather than a transient activation state (**Supplementary Figure S1**).

Heatmap visualization confirmed distinct expression patterns of key polarization markers, including CD68, IL1B, MRC1, and SPHK1, among macrophage subsets (**Figures 2H–I**). Notably, tSNE co-localization plots indicated that SPHK1 expression was preferentially enriched in the MRC1^+^ fraction of MARCO-1 cells (**Figure 2J**), suggesting that SPHK1 may align with the M2-oriented trajectory despite underlying heterogeneity. In support of this, pseudotime trajectory analysis reconstructed the transition from monocytes to macrophages (**Figure 2K**), showing that CD163, MRC1, and SPHK1 expression progressively increased along differentiation.

### SPHK1 expression is upregulated during thrombus progression and co-expressed with M2 macrophages

**Figure 3:**
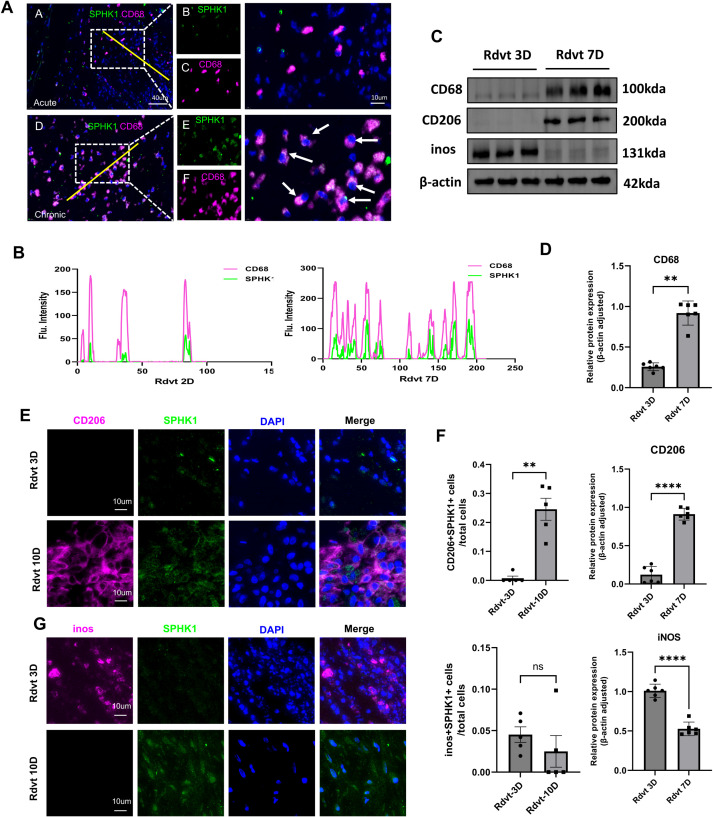
SPHK1 is associated with macrophage polarization and thrombus fibrosis during thrombus progression in a rat model. **(A)** Representative immunofluorescence images of SPHK1 (green) and CD68 (red) co-staining in thrombus tissues from rats at 3 days (Rdvt3D) and 7 days (Rdvt7D) after IVC ligation. Nuclei were counterstained with DAPI. **(B)** Fluorescence intensity profiles along the yellow line in merged images of SPHK1 and CD68, showing co-localization. **(C)** Western blot analysis of CD68, CD206 and iNOS expression in thrombus tissues from Rdvt3D and Rdvt7D rats (n = 6). **(D)** Quantification of Western blot band intensities in panel **(C)** Data are presented as mean ± SD. **(E, G)** Dual immunofluorescence staining of SPHK1 with CD206 **(E)** and iNOS **(G)** in thrombus tissues from 3D and 7D groups. **(F)** Quantitative analysis of co-localization coefficients between SPHK1 and CD206 or iNOS using ImageJ software (n = 5). **P<0.01, ****P<0.0001.

To further investigate the relationship between SPHK1 expression and macrophage polarization, we performed a systematic analysis of thrombus tissues at day 3 and day 7 following inferior vena cava (IVC) ligation in a rat model. Dual immunofluorescence staining for SPHK1 and CD68 revealed that SPHK1 was predominantly expressed in CD68^+^ macrophages, with a marked increase in expression observed on day 7 (**Figure 3A**). Fluorescence intensity profiles confirmed substantial spatial overlap between SPHK1 and CD68 (**Figure 3B**). Subsequently, we conducted Western blot analysis to assess the expression of key fibrosis- and polarization-related proteins in thrombus tissue. Results showed that CD68, and the M2 marker CD206 were significantly upregulated by day 7, while the M1 marker iNOS was notably downregulated (**Figures 3C, D**). To clarify the cellular source of iNOS expression at day 3, we further performed double immunofluorescence staining for iNOS and CD68. The results confirmed that iNOS signal was localized within CD68^+^ macrophages (**Supplementary Figure S2**). To further define the distribution of SPHK1 in different macrophage polarization states, we performed dual immunofluorescence staining for SPHK1 with CD206 (M2 marker) and iNOS (M1 marker). SPHK1 was found to be highly enriched in CD206^+^ macrophages, whereas its co-localization with iNOS was limited (**Figures 3E, G**). Quantitative co-localization analysis using ImageJ confirmed that the overlap coefficient between SPHK1 and CD206 was significantly higher than that between SPHK1 and iNOS (**Figure 3F**).

### SPHK1 knockdown reprograms the immune and metabolic phenotypes of M2 macrophages

**Figure 4:**
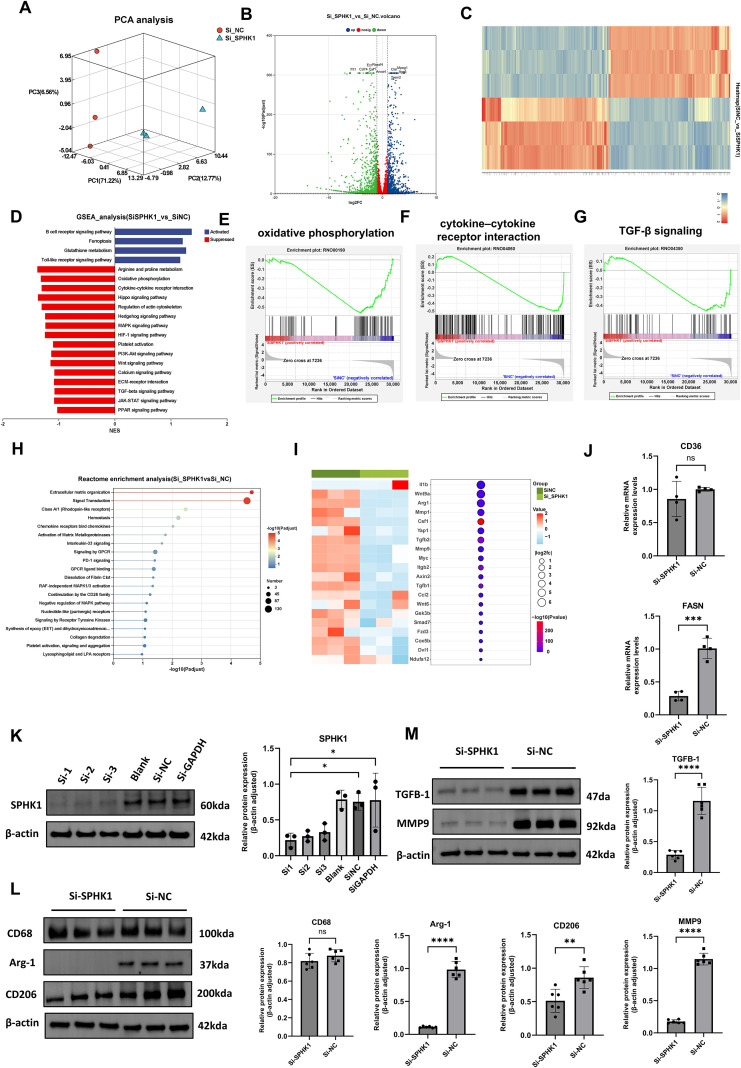
Effects of SPHK1 knockdown on gene expression and signaling pathway. **(A)** PCA analysis showed distinct transcriptomic separation between siSPHK1 and control groups. **(B)** Volcano plot revealed widespread differential gene expression. **(C)** Heatmap demonstrated clear clustering of samples between groups. **(D)** GSEA showed upregulation of ferroptosis and glutathione metabolism pathways, while metabolic and signaling pathways were downregulated in the siSPHK1 group. **(E–G)** GSEA indicated suppression of oxidative phosphorylation, cytokine–cytokine receptor interaction, and Hippo signaling pathways. **(H)** Reactome pathway analysis revealed enrichment in extracellular matrix organization and signal transduction. **(I)** Bubble and heatmap plots showed downregulation of fibrosis- and immune-related genes following SPHK1 knockdown. **(J)** RT-qPCR (n = 3) confirmed consistent downregulation of CD36, and FASN. **(K)** Western blot screening of three siRNAs (n = 3) showed effective SPHK1 knockdown at the protein level. **(L)** Western blot analysis (n = 6) showed decreased Arg-1 and CD206 expression in the siSPHK1 group, with no significant change in CD68. **(M)** SPHK1 knockdown also reduced TGF-β1 and MMP9 protein expression (n = 6). *P<0.05, **P<0.01, ***P<0.001, ****P<0.0001, “ns” indicates not significant.

To further validate SPHK1 expression in macrophages and establish an *in vitro* platform for mechanistic studies, we isolated and cultured rat bone marrow-derived macrophages (BMDMs). Flow cytometry confirmed a high purity of the macrophage population, with CD11b^+^CD68^+^ double-positive cells accounting for 92.7% of the total population (**Supplementary Figure S3**), providing a reliable cellular model for subsequent polarization and signaling pathway investigations. To evaluate the transcriptional regulatory role of SPHK1 in M2-polarized macrophages, we established an M2 polarization model using rat bone marrow-derived macrophages (BMDMs) stimulated with IL-4 and IL-13. SPHK1 expression was silenced using siRNA (siSPHK1 group), and compared with a negative control (siNC group). Principal component analysis (PCA) revealed a clear transcriptional separation between the siSPHK1 and siNC groups, indicating that SPHK1 knockdown led to extensive gene expression reprogramming (**Figure 4A**). A volcano plot showed widespread differential expression of genes, including both upregulated and downregulated transcripts (**Figure 4B**). Hierarchical clustering also demonstrated distinct transcriptomic profiles between the two groups (**Figure 4C**). Gene set enrichment analysis (GSEA) revealed that pathways upregulated in the siSPHK1 group included B cell receptor signaling, ferroptosis, and glutathione metabolism, suggesting the activation of immune responses and oxidative stress defense following SPHK1 inhibition (**Figure 4D**). In contrast, downregulated pathways were enriched in classical signaling and metabolic processes, such as arginine and proline metabolism, oxidative phosphorylation (**Figure 4E**), cytokine–cytokine receptor interaction (**Figure 4F**), Hippo signaling, PI3K-Akt, and TGF-β signaling (**Figure 4G**). Reactome pathway enrichment analysis further revealed significant enrichment in ECM organization and signal transduction pathways (**Figure 4H**), implying that SPHK1 may regulate macrophage interaction with the microenvironment, thereby influencing tissue fibrosis and immune responses. We visualized representative genes associated with inflammation, lipid metabolism, chemotaxis, and fibrosis using combined heatmaps and bubble plots (**Figure 4I**).

To validate the RNA-seq results, we performed RT-qPCR for two key functional genes in M2 macrophages: CD36, and FASN. Two genes were significantly downregulated in the siSPHK1 group compared to the siNC group (**Figure 4J**). To identify the most effective SPHK1-targeting siRNA and confirm knockdown efficiency, three siRNA sequences (Si1, Si2, Si3) were transfected into BMDMs, western blot analysis showed that all three siRNAs effectively reduced SPHK1 protein expression, with Si1 achieving the highest silencing efficiency (**Figure 4K**). Si1 was selected for subsequent functional studies. Western blot results demonstrated that SPHK1 knockdown significantly decreased protein expression of Arg-1 and CD206, while CD68 levels remained unchanged, suggesting that SPHK1 primarily regulates macrophage functional status rather than cell numbers (**Figure 4L**). Additionally, both TGF-β1 and MMP9 levels were markedly reduced (**Figure 4M**), indicating that SPHK1 silencing suppresses the pro-fibrotic potential of M2 macrophages. These protein-level findings further support the pivotal role of SPHK1 in maintaining M2 polarization and fibrotic activity.

### M2 macrophages promote STAT3 activation in fibroblasts through the secretion of TGF-β1

**Figure 5:**
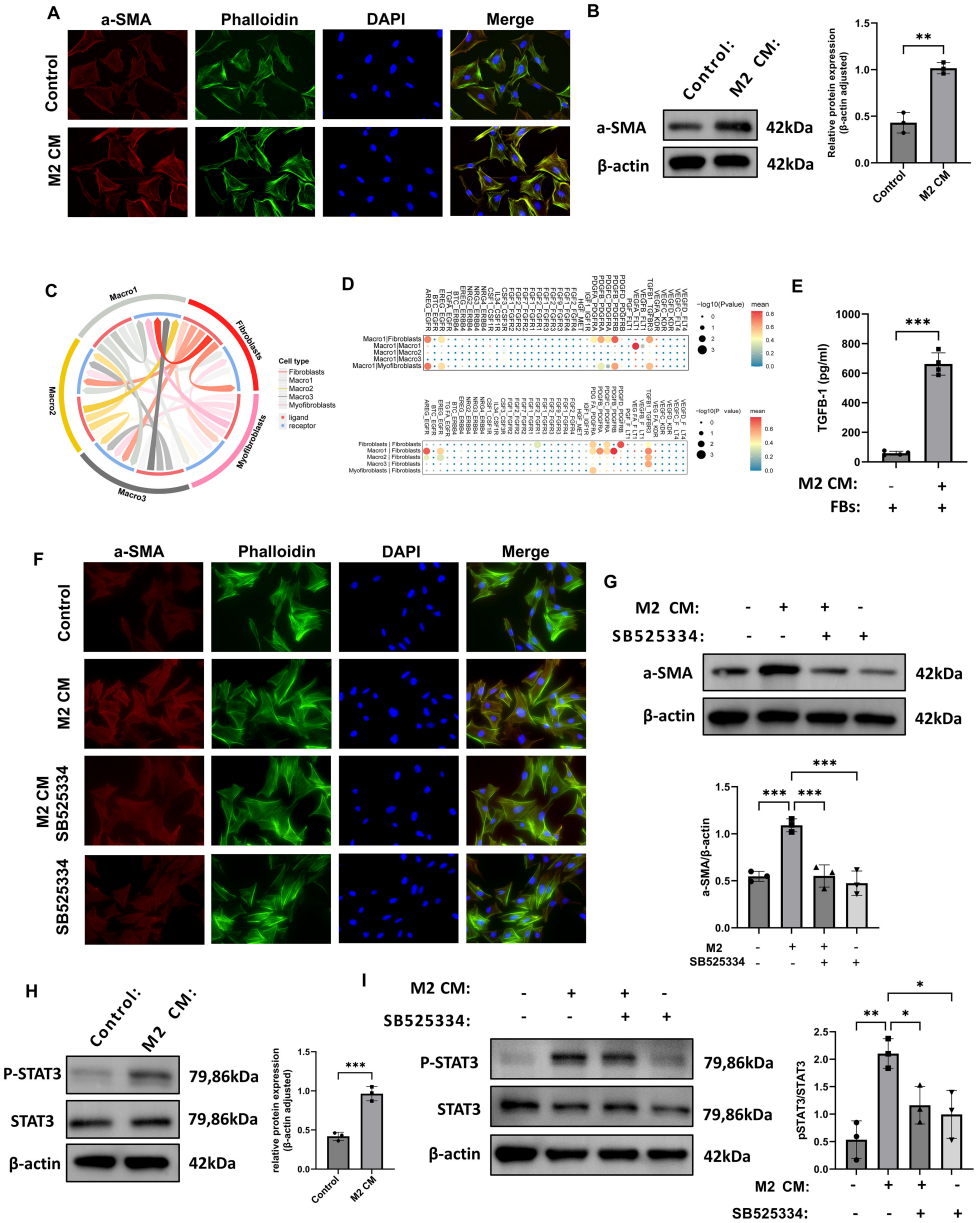
M2 macrophages induce fibroblast-to-myofibroblast transition via TGF-β1-mediated STAT3 signaling. **(A)** Immunofluorescence images showing increased α-SMA in fibroblasts treated with M2 macrophage-conditioned medium (CM). **(B)** Western blot confirms α-SMA upregulation after M2 CM treatment (n = 3). **(C, D)** Ligand–receptor bi-directional interaction analysis based on scRNA-seq data illustrating the intercellular communication network between macrophages and fibroblasts. **(E)** ELISA results showed increased TGF-β1 concentration in the supernatant of fibroblasts co-cultured with M2 CM. **(F)** Immunofluorescence analysis showed that treatment with the TGF-β1 receptor inhibitor SB525334 suppressed the α-SMA fluorescence signal induced by M2 CM. **(G)** Western blot analysis demonstrated that SB525334 reversed the M2 CM-induced upregulation of α-SMA protein expression (n = 3). **(H)** Western blot analysis of fibroblasts revealed that M2 CM stimulation significantly increased the level of phosphorylated STAT3 (P-STAT3), while total STAT3 expression remained unchanged (n = 3). **(I)** Western blot analysis of fibroblasts revealed that SB525334 reduced P-STAT3 levels to near control (n = 3). *P<0.05, **P<0.01, ***P<0.001.

To further investigate the regulatory role of M2 macrophages in fibroblast phenotypic transformation, we examined the expression of the myofibroblast marker α-smooth muscle actin (α-SMA). Immunofluorescence staining revealed that α-SMA expression was markedly increased in fibroblasts stimulated with conditioned medium (CM) from M2 macrophages, indicating that paracrine factors derived from M2 macrophages can induce fibroblast-to-myofibroblast transdifferentiation (**Figure 5A**). Consistently, Western blot analysis showed a significant upregulation of α-SMA protein levels in the co-culture group, further confirming the pro-fibrotic induction effect at the molecular level (**Figure 5B**). We performed cell–cell communication analysis based on single-cell RNA sequencing data (**Figure 5C**). Ligand–receptor interaction mapping revealed strong signaling connections between fibroblasts and Macro1-derived profibrotic ligands (**Figure 5D**).

To functionally validate the pro-fibrotic effect of M2 macrophages, we isolated and cultured primary fibroblasts from rat thrombus tissue and exposed them to M2 CM. ELISA revealed significantly increased levels of TGF-β1 in the supernatants of co-cultured cells (**Figure 5E**). Immunofluorescence staining demonstrated that α-SMA signal intensity was markedly increased in fibroblasts exposed to M2 CM, while the TGF-β1 receptor inhibitor SB525334 effectively reduced this signal (**Figure 5F**). Western blot analysis confirmed that α-SMA protein levels were significantly elevated in the M2 CM group compared with controls, and SB525334 treatment reversed this effect (**Figure 5G**). Signal transducer and activator of transcription 3 (STAT3) is a key transcription factor involved in inflammation, immune regulation, and tissue remodeling. Western blot results showed that P-STAT3 levels were significantly increased in fibroblasts treated with M2 CM, while total STAT3 levels remained unchanged, indicating phosphorylation-dependent activation of the pathway (**Figure 5H**). Furthermore, treatment with SB525334 reduced P-STAT3 expression to baseline levels, without affecting total STAT3 protein (**Figure 5I**). Collectively, these results demonstrate that M2 macrophages promote fibroblast activation through TGF-β1-mediated phosphorylation of STAT3, potentially contributing to pro-fibrotic responses during thrombus remodeling.

### SPHK1 inhibitor PF543 suppresses thrombus fibrosis and M2 macrophage polarization *in vivo*

**Figure 6:**
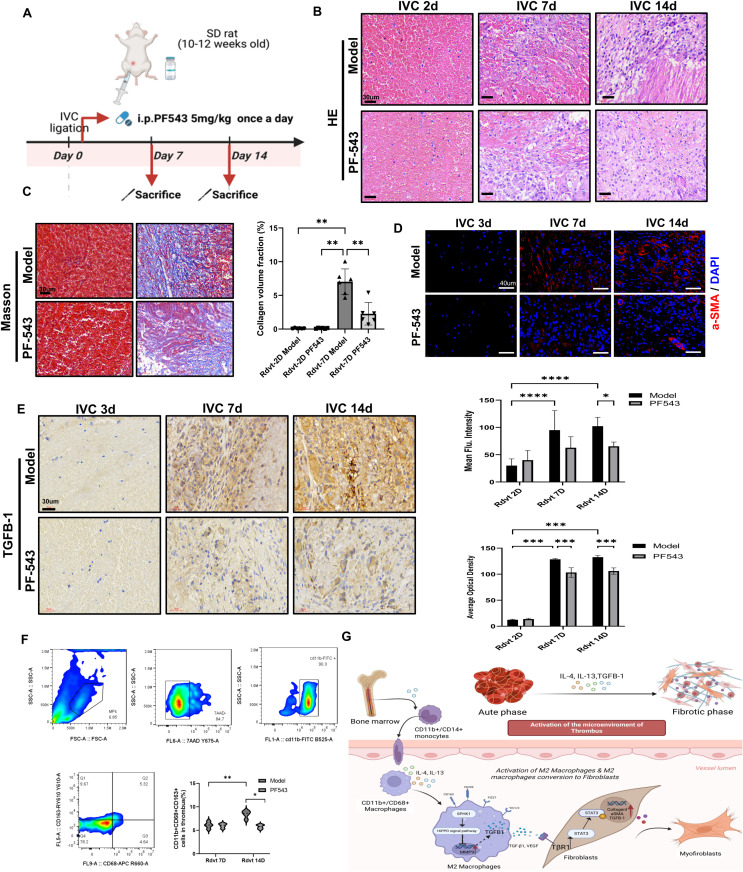
PF543 attenuates venous thrombus fibrosis and modulates the immune microenvironment *in vivo*. **(A)** Schematic diagram of the animal experimental protocol. **(B)** Hematoxylin and eosin (HE) staining of thrombus tissues at 2, 7, and 14 days. **(C)** Masson’s trichrome staining at days 2 and 7 showed collagen fiber deposition. The bar graph quantifies collagen-positive area (n = 6). **(D)** Immunofluorescence staining of α-SMA at days 2, 7, and 14. The bar graph presents mean fluorescence intensity quantification (n = 6). **(E)** Immunohistochemical staining of TGF-β1 at days 3, 7, and 14. The bar graph quantifies the TGF-β1-positive area (n = 4). **(F)** Flow cytometry gating strategy to identify CD163^+^ M2 macrophages within thrombus tissue. The bar graph shows the proportion of CD163^+^ macrophages in each group (n = 5). **(G)** Schematic illustration of the proposed mechanism. Bone marrow-derived CD11b^+^/CD14^+^ monocytes differentiate into CD11b^+^/CD68^+^ macrophages, which are polarized into M2 macrophages by IL-4 and IL-13. M2 macrophages express markers such as Ym1/2, FIZZ1, CD206, and CD163, and secrete TGF-β1, VEGF, and MMP9. These factors activate the TβR1/STAT3 pathway, which promotes fibroblast activation (e.g., α-SMA and Collagen I expression) and tissue remodeling. *P<0.05, **P<0.01, ***P<0.001, ****P<0.0001.

To further investigate the functional role of SPHK1 in thrombus fibrosis, we employed a rat model of venous thrombosis established by inferior vena cava (IVC) ligation, and administered the selective SPHK1 inhibitor PF543 via intraperitoneal injection (**Figure 6A**). H&E staining revealed progressive compaction of thrombus structure and increased cellular infiltration over time in the model group, indicating ongoing fibrotic remodeling. In contrast, PF543-treated rats exhibited looser tissue architecture and reduced inflammatory cell infiltration at days 2, 7, and 14 (**Figure 6B**). Masson’s trichrome staining further demonstrated a marked reduction in collagen fiber deposition in the PF543 group, indicating effective inhibition of thrombus fibrosis (**Figure 6C**). At the molecular level, immunofluorescence staining for α-SMA showed that PF543 significantly suppressed myofibroblast accumulation and activation (**Figure 6D**). Immunohistochemical staining for TGF-β1 also revealed markedly decreased expression in thrombus tissue following PF543 treatment (**Figure 6E**). Flow cytometric analysis of macrophage polarization indicated a significant enrichment of CD163^+^ M2 macrophages in the model group at both day 7 and day 14, whereas PF543 treatment effectively reduced the proportion of M2-polarized cells (**Figure 6F**). Taken together, these findings demonstrate that the SPHK1 inhibitor PF543 attenuates thrombus fibrosis *in vivo* by downregulating TGF-β1 expression and inhibiting M2 macrophage polarization, thereby mitigating the progression of thrombus remodeling (**Figure 6G**).

## Discussion

SPHK1 is a key enzyme in the sphingolipid metabolic pathway and has been widely studied in the context of cancer, cardiovascular disease, and organ fibrosis, including liver and pulmonary fibrosis (18–20). However, its role in the pathological context of thrombosis, particularly in chronic thrombus fibrosis, has not been explored. Our study is the first to reveal the role of SPHK1 in thrombus fibrosis in CTEPH, particularly in the polarization of M2 macrophages. While these findings align with existing literature, we believe this new application broadens the role of SPHK1 in the immune-fibrotic axis and provides a new direction for future research.

By comparing thrombus tissues from CTEPH and acute pulmonary embolism patients, we found significantly increased ECM deposition, accumulation of α-SMA^+^ fibrotic cells, and infiltration of CD68^+^ macrophages in CTEPH samples, suggesting the key roles of macrophages and fibroblasts in the fibrosis process. Additionally, there was a significant upregulation of IL-4, IL-13, and TGF-β1 expression in proximal pulmonary endarterectomy tissues. TGF-β1 promotes the differentiation of fibroblasts into myofibroblasts and forms a positive feedback loop with M2 macrophages (21, 22), amplifying fibrotic signals and driving lesion progression. This mechanism may explain the stabilization of chronic thrombi and the therapeutic challenges in CTEPH, as well as the persistent pulmonary hypertension in some patients even after surgical intervention.

This study revealed the complex cellular landscape of CTEPH thrombus tissue through single-cell RNA sequencing analysis and identified a distinct macrophage subpopulation, termed Macro1. Gene set scoring indicated that the Macro1 subpopulation exhibited high expression of genes associated with inflammation, lipid metabolism, and pro-angiogenic activity, suggesting that Macro1 macrophages display a functionally active “mixed polarization” phenotype. Notably, SPHK1 was highly expressed in the Macro1 subpopulation and co-expressed with M2 markers. Immunofluorescence analysis further confirmed that the co-expression of SPHK1 with M2 markers increased, particularly during disease progression, whereas co-localization with M1 markers was relatively limited. Consistent with previous studies, SPHK1 has been shown to regulate macrophage polarization, particularly in M2 macrophages, in various fibrotic diseases (18, 23). Future research will need to verify the stability of the Macro1 subpopulation and further investigate its role in the fibrosis process through longer-term dynamic tracking and marker analysis.

Our functional experiments demonstrated that SPHK1 knockdown not only suppressed the expression of pro-fibrotic and immunometabolic molecules but also significantly weakened the phenotypic characteristics of M2 macrophages, indicating that SPHK1 plays a multifaceted role in maintaining the pro-fibrotic identity of M2 macrophages. Moreover, SPHK1 knockdown led to the downregulation of several pathways related to ECM remodeling and microenvironmental reprogramming, including the TGF-β, PI3K-Akt, and oxidative phosphorylation pathways. These results are consistent with the literature, as TGF-β and PI3K-Akt signaling pathways play important roles in fibroblast activation, proliferation, and collagen deposition (24, 25). In studies of pulmonary and liver fibrosis, oxidative phosphorylation has been shown to be a key metabolic pathway in fibrosis, regulating redox reactions and participating in immune cell polarization (26, 27). Future research will further explore the relationship between SPHK1-regulated metabolic pathways, immune responses, and fibrotic phenotypes through methods such as mitochondrial function assays.

Our study shows that SPHK1 downstream signaling regulates TGF-β1 production and is associated with the phosphorylation and activation of STAT3. In the macrophage–fibroblast co-culture system, we observed a significant increase in P-STAT3 levels, which could be effectively inhibited by SB525334, confirming the role of the TGF-β1–STAT3 signaling axis in intercellular communication. Recent studies have revealed that M2 macrophages remodel the fibrotic microenvironment through paracrine mechanisms, promoting fibroblast activation and disrupting tissue homeostasis (28, 29). Our research extends this mechanism to thrombus-associated fibrosis, filling the knowledge gap regarding macrophage–fibroblast interactions. Previous studies have shown that SPHK1 regulates the production of sphingosine-1-phosphate (S1P), which may modulate fibrosis-related pathways such as TGF-β1 and STAT3 through S1P receptors (24, 30, 31). Additionally, S1P receptors play a crucial role in fibrosis, particularly in pulmonary fibrosis models, where S1P promotes fibroblast migration and activation via S1P receptors 1 and 3 (20, 32). These studies support our findings and provide a foundation for future research to validate the interaction between the SPHK1–S1P axis and the TGF-β1–STAT3 signaling pathway.

We used the rat inferior vena cava ligation model to simulate thrombus formation and its fibrotic response. Due to the strong fibrinolytic activity in animals, thrombi are prone to dissolution, making it difficult to fully replicate the characteristics of organized thrombi that persist in CTEPH, which presents challenges in modeling the chronic progression and immune response of CTEPH (33, 34). Nevertheless, previous studies have shown that this model partially recapitulates the chronicity of thrombi and provides experimental evidence for thrombus fibrosis and immune response (35, 36). In our *in vivo* experiments, the use of the PF543 inhibitor to suppress SPHK1 significantly reduced thrombus fibrosis in rats, as evidenced by decreased TGF-β1 expression, reduced accumulation of α-SMA^+^ myofibroblasts, and a significant decrease in CD163^+^ M2 macrophages. These results provide *in vivo* validation of SPHK1’s role in regulating the M2 polarization–pro-fibrotic axis. To more accurately simulate the chronic progression of CTEPH, future studies may require more complex animal models, such as those involving fibrinolysis inhibition or chronic immune response simulations, to better replicate the pathological features of CTEPH.

Although this study provides preliminary evidence of SPHK1’s role in thrombus fibrosis, there are still limitations. First, the PF543 pharmacological inhibitor may have off-target effects. Future research will validate these findings using genetic models such as SPHK1 knockout mice to eliminate non-specific effects. Second, the current IL-4 and IL-13 *in vitro* polarization model does not fully replicate the immune microenvironment of CTEPH. Future studies will use additional cytokines and more complex models to more accurately reflect the pathological features of CTEPH. Finally, due to the limited sample size of CTEPH samples and potential selection bias, future research will expand the sample size and incorporate multi-center data to enhance the reliability of the results. Although PF543, as an SPHK1 inhibitor, played a role in this study, its dosage and therapeutic window have not been optimized. Future studies will assess its efficacy and safety in chronic diseases through dose-gradient experiments and long-term pharmacological research.

In summary, Our study provides multi-dimensional evidence that SPHK1 plays a critical pro-fibrotic role in venous thrombus remodeling. SPHK1 is highly expressed in M2 macrophages, where it promotes TGF-β1 secretion and activates the STAT3 signaling pathway in fibroblasts, thereby driving their transdifferentiation into myofibroblasts and accelerating thrombus fibrosis. Pharmacological inhibition of SPHK1 using PF543 effectively disrupts this process, alleviates fibrotic progression, and suppresses M2 polarization. Future studies will use gene knockout models and more in-depth mechanistic analyses to further elucidate the role of SPHK1 and its interactions with key pathways such as the TGF-β1–STAT3 axis. Additionally, optimizing therapeutic strategies based on more detailed pharmacological research will be key to confirming the therapeutic potential of SPHK1 in CTEPH and similar diseases.

## Data Availability

The datasets presented in this study can be found in online repositories. The names of the repository/repositories and accession number(s) can be found in the article/**Supplementary Material**.
