# Supplementary material for: SPHK1-mediated M2 macrophage polarization drives TGF-β1-dependent thrombus fibrosis
Source: Front Immunol. 2025 Nov 17;16:1681485. doi: 10.3389/fimmu.2025.1681485 (PMC12665702; doi:10.3389/fimmu.2025.1681485)
Supplement: Supplementary file 4 [file Table1.docx]

**Uncropped Gels and Blots image(s) in Figure 1C**

**
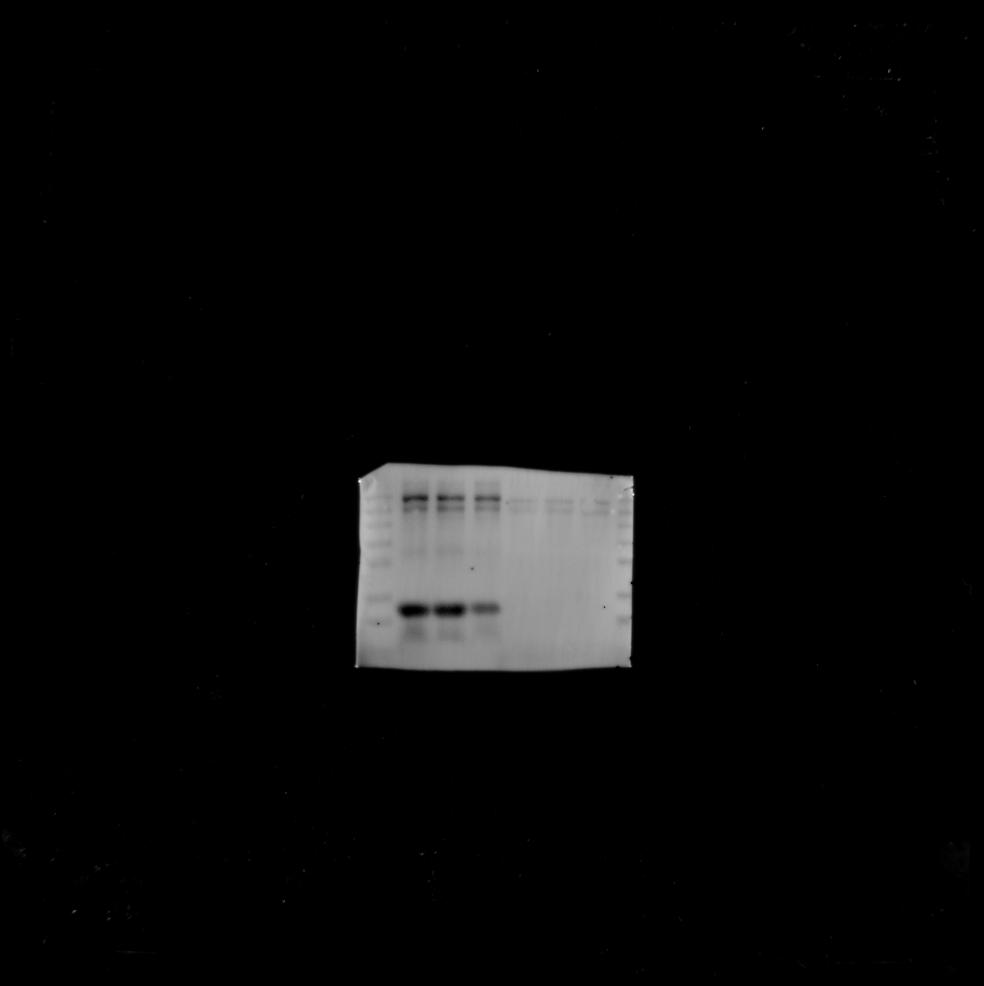

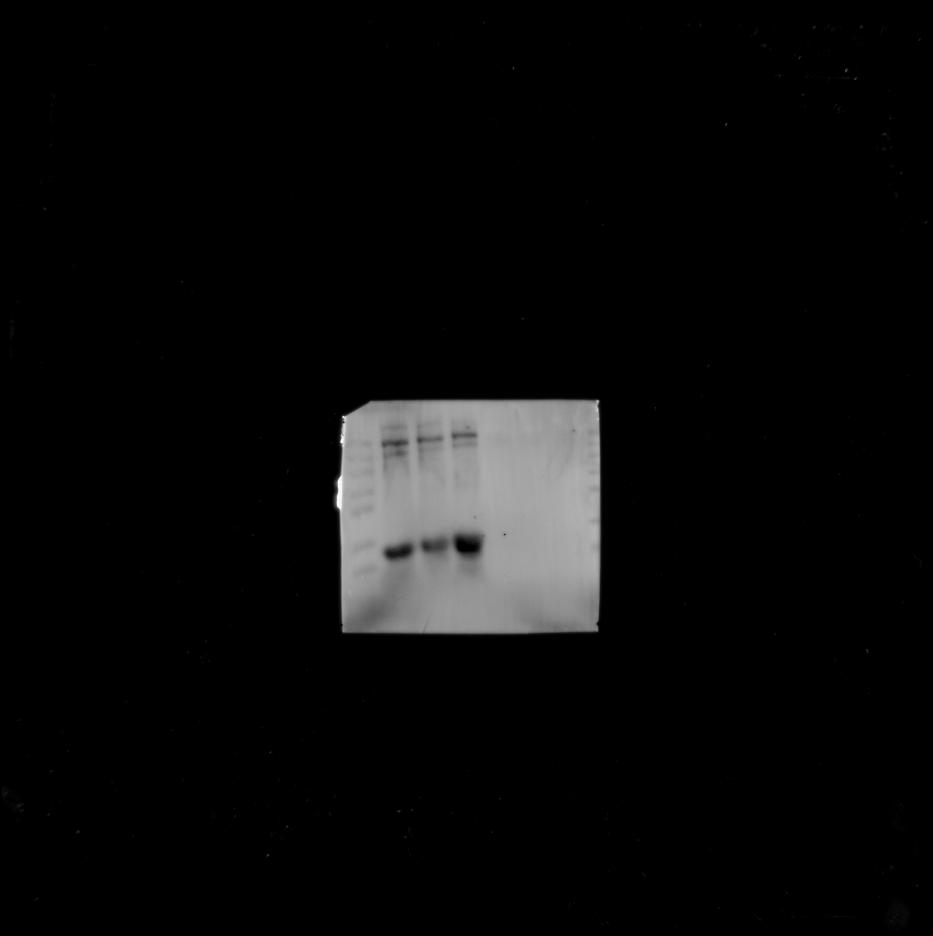
**

**17kda**

**10kda**

**IL-13**

**IL-13**

**
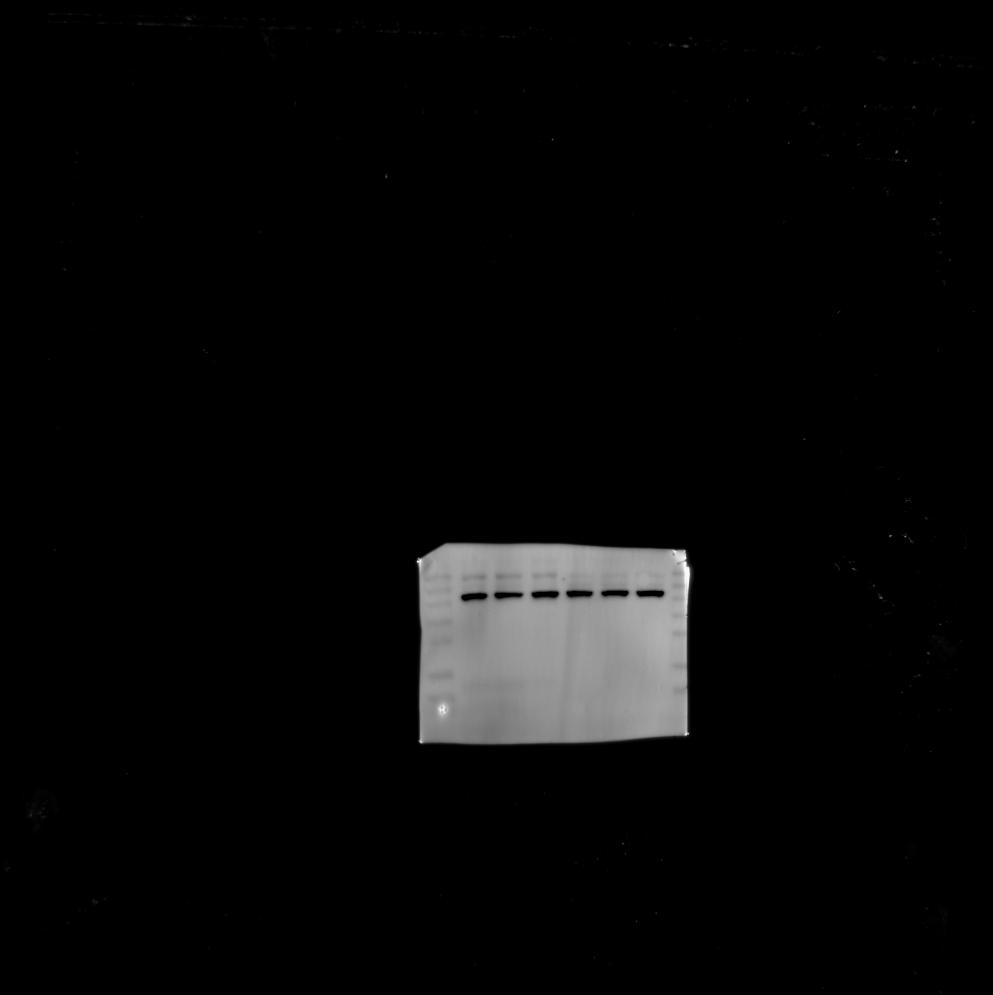

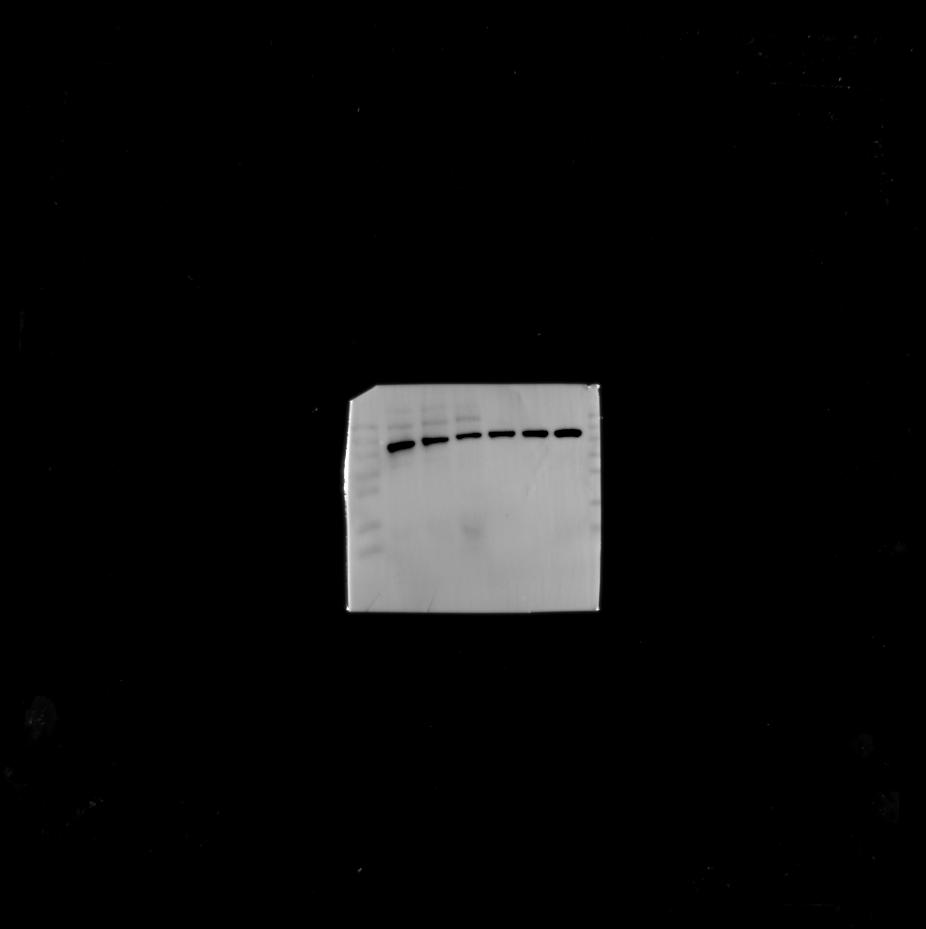
**

**55kda**

**75kda**

**43kda**

**β-actin**

**β-actin**

**
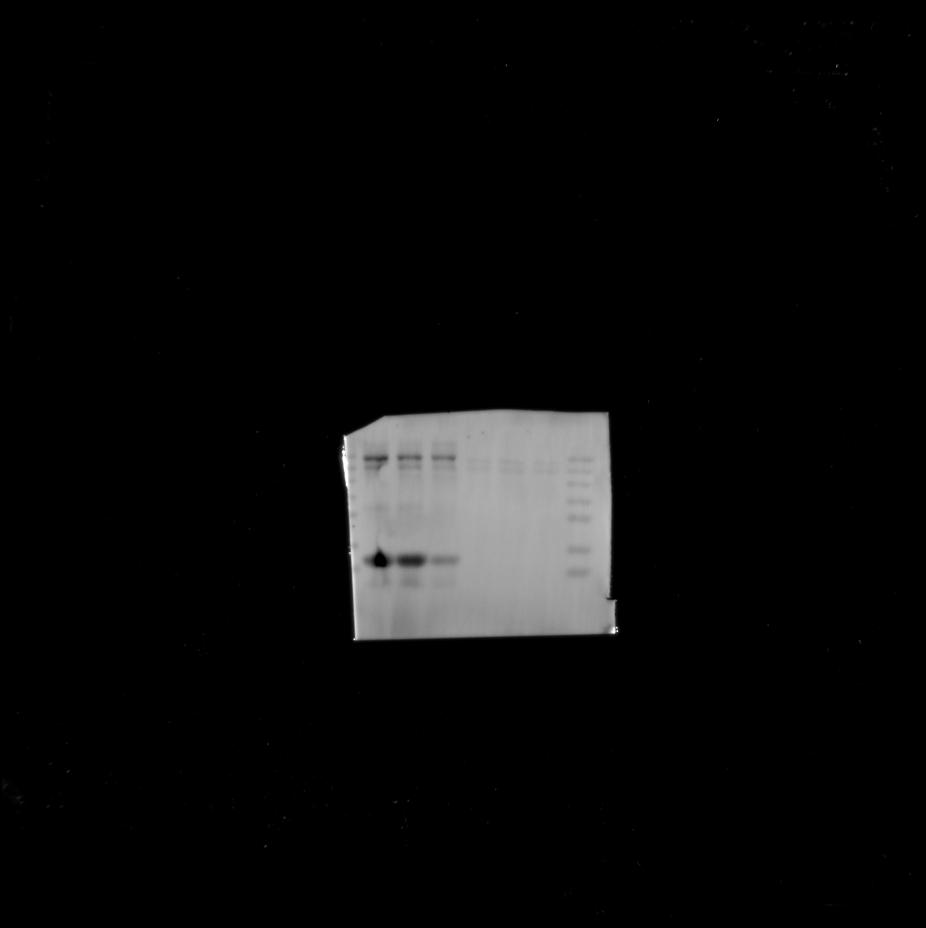
**

**
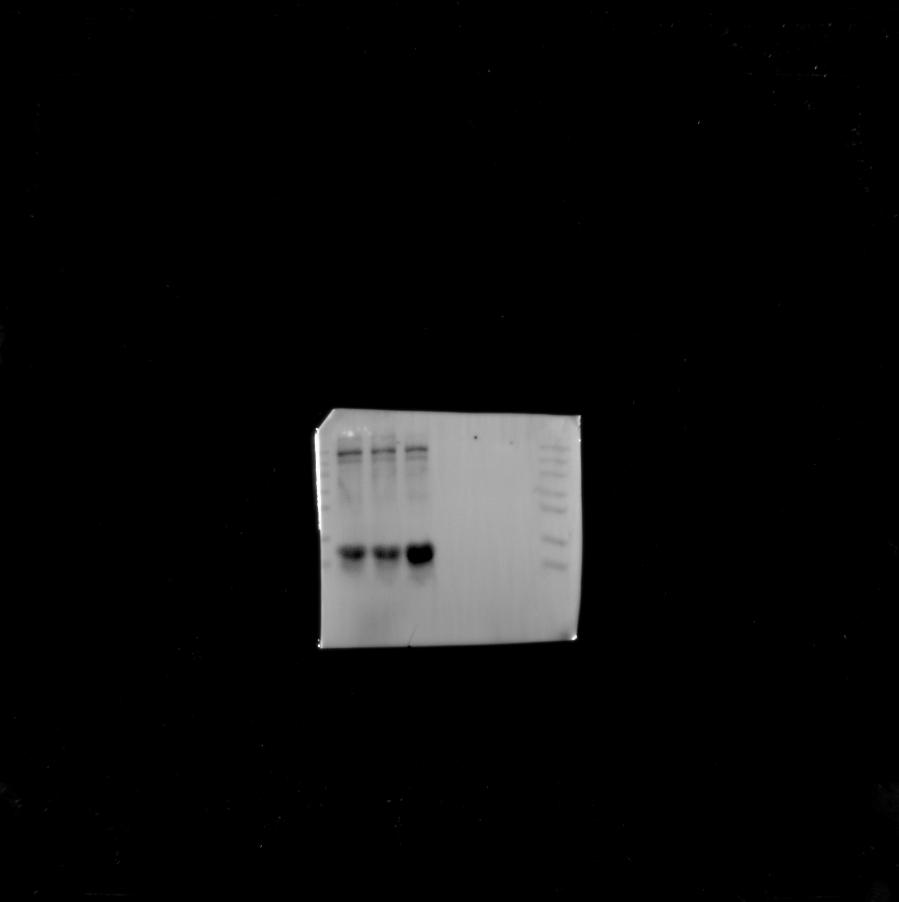
**

**17kda**

**10kda**

**IL-4**

**IL-4**

**
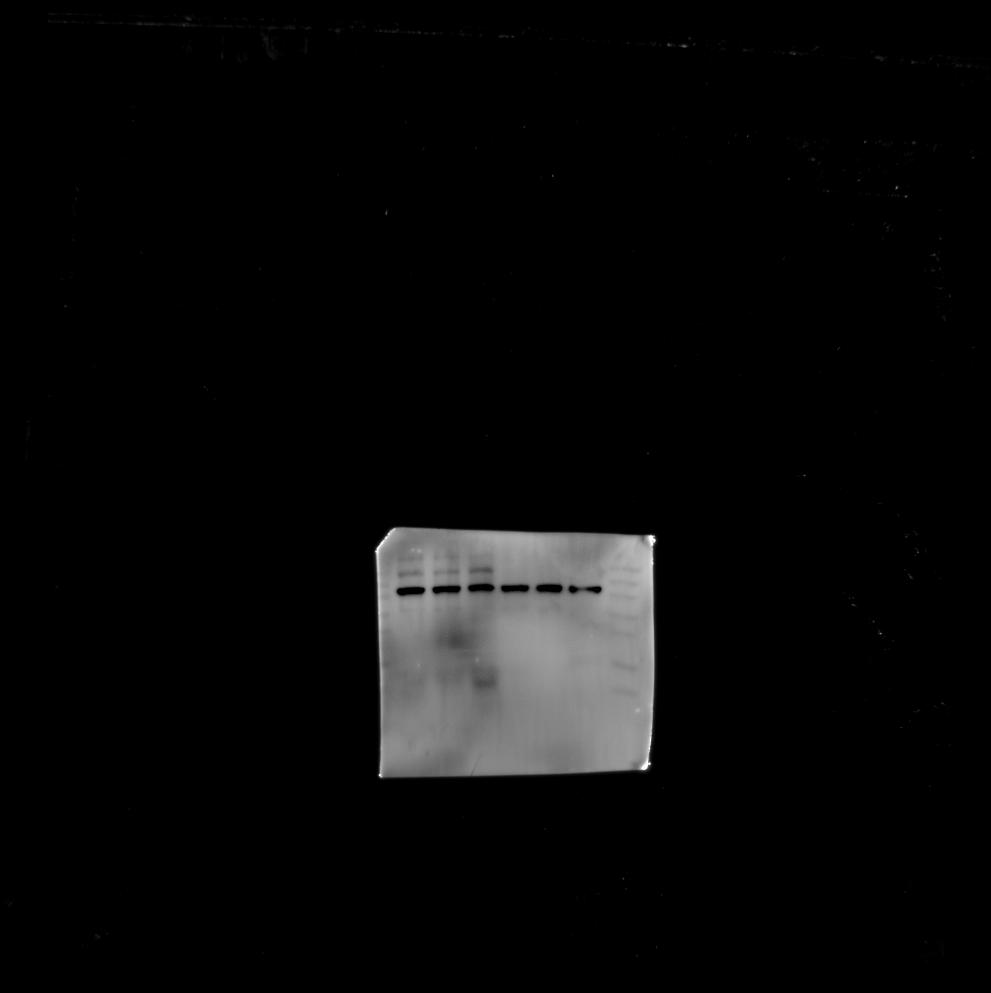

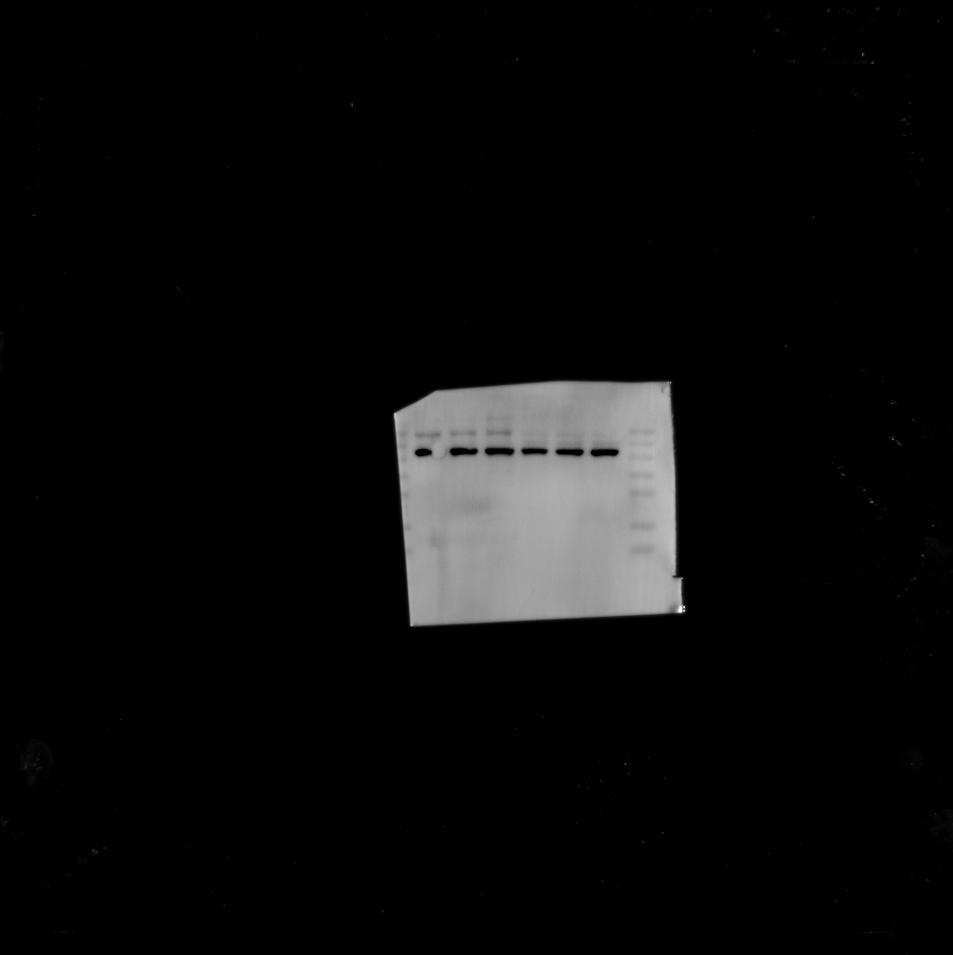
**

**75kda**

**55kda**

**43kda**

**β-actin**

**β-actin**

**
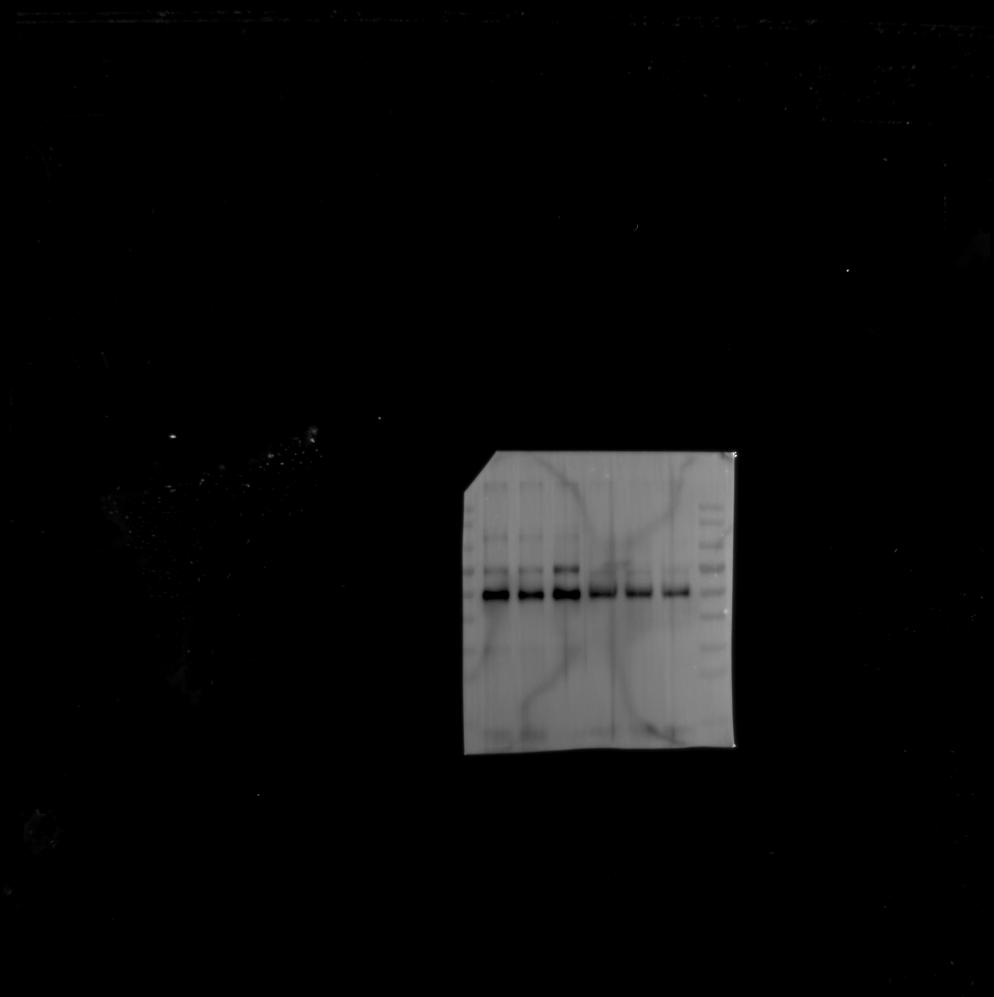

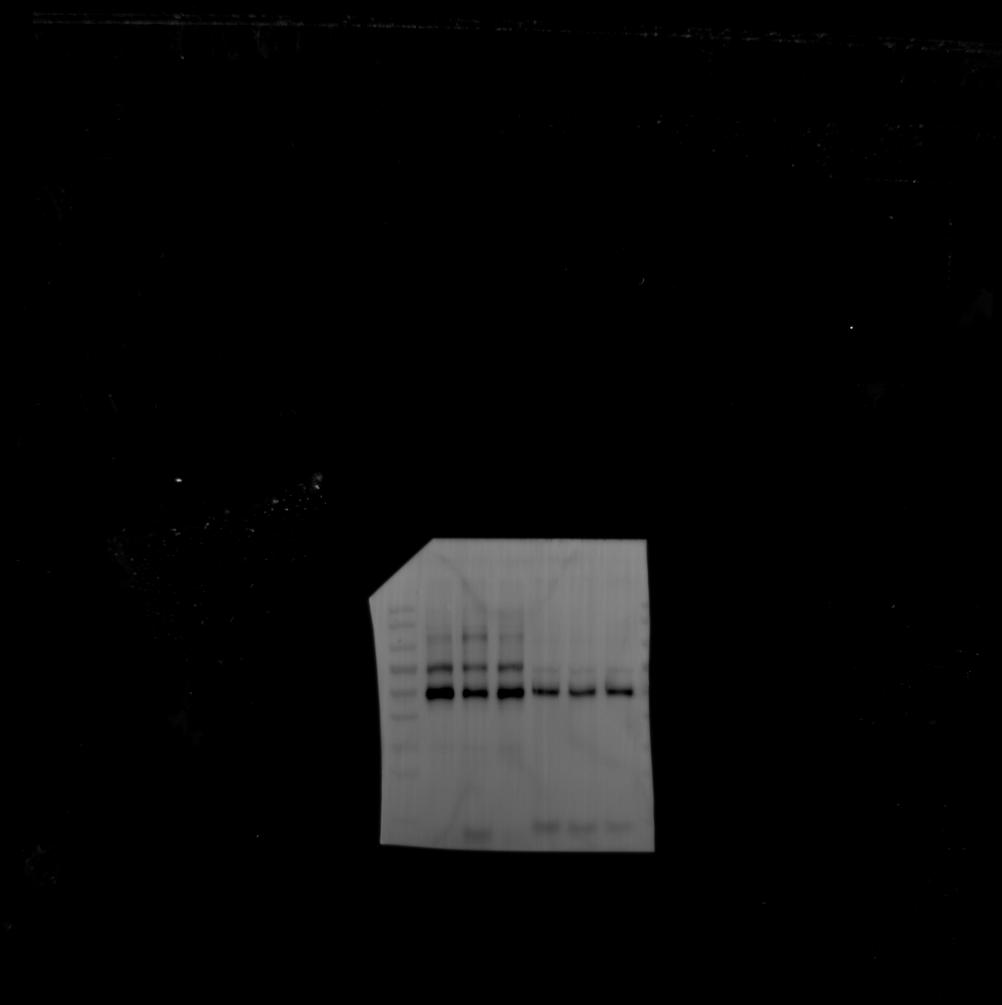
**

**75kda**

**55kda**

**43kda**

**TGFB-1**

**TGFB-1**

**
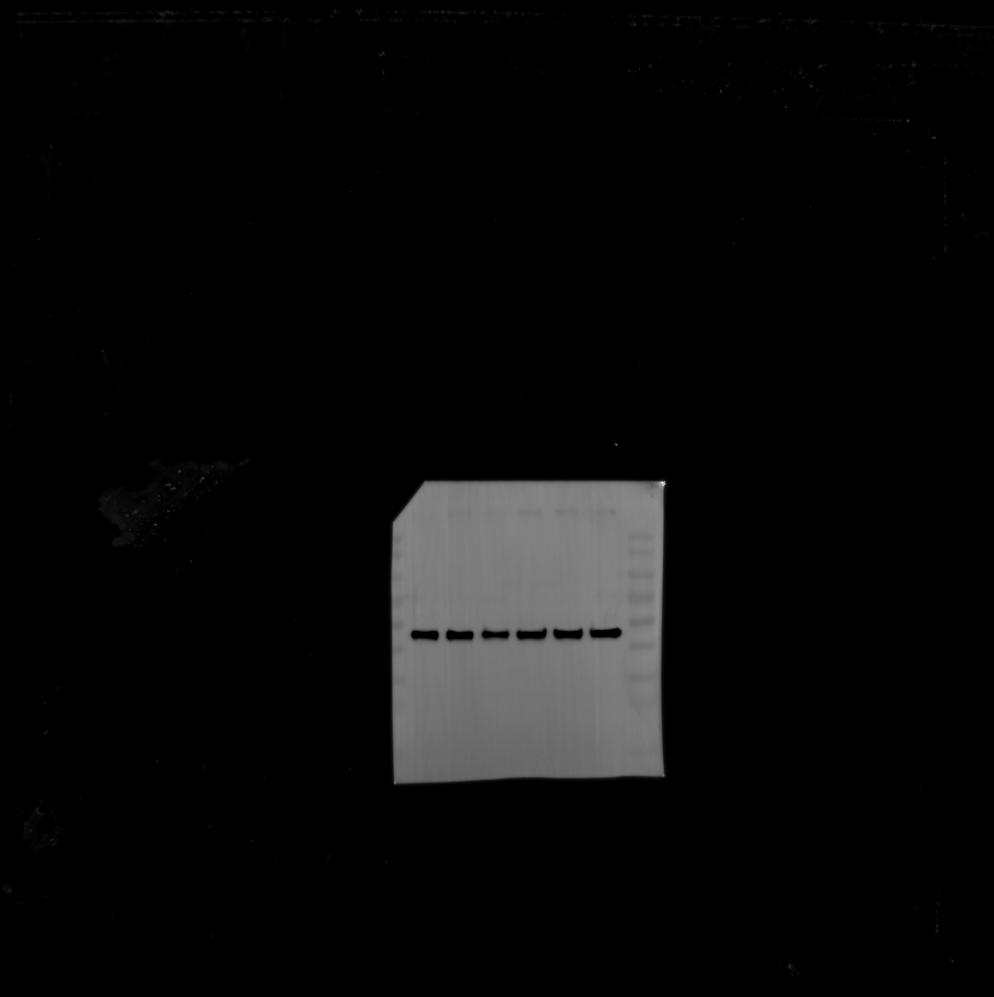
**

**
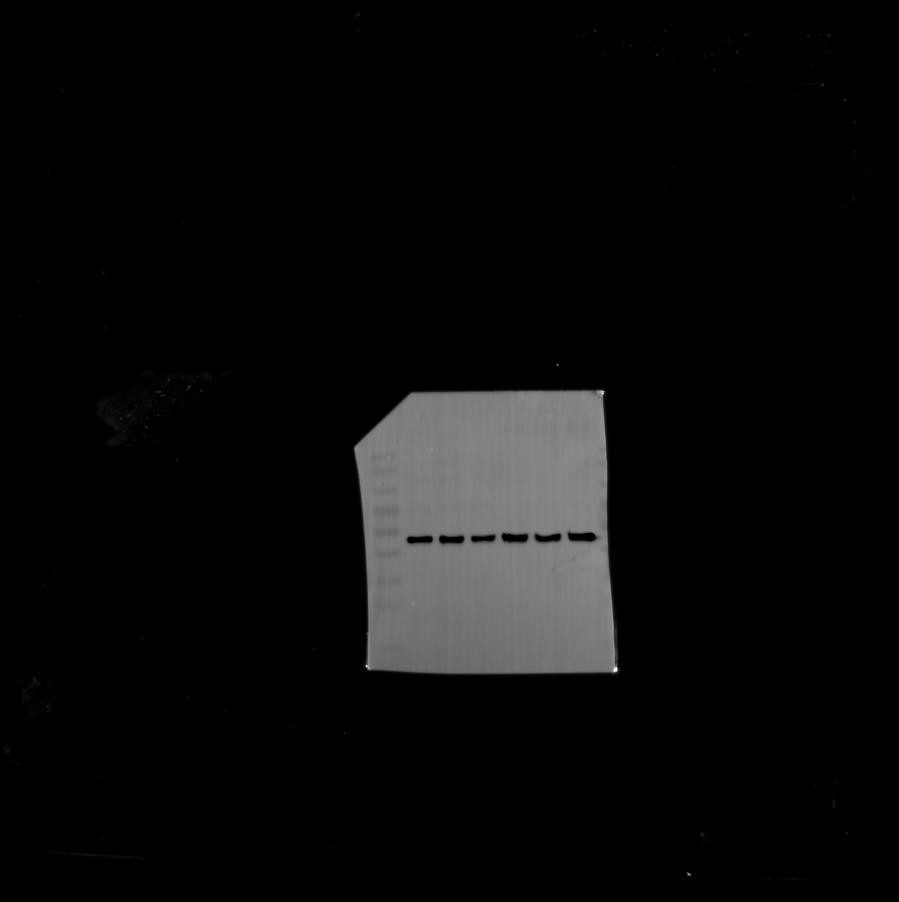
**

**75kda**

**55kda**

**43kda**

**β-actin**

**β-actin**

**Uncropped Gels and Blots image(s) in Figure 1D**

**
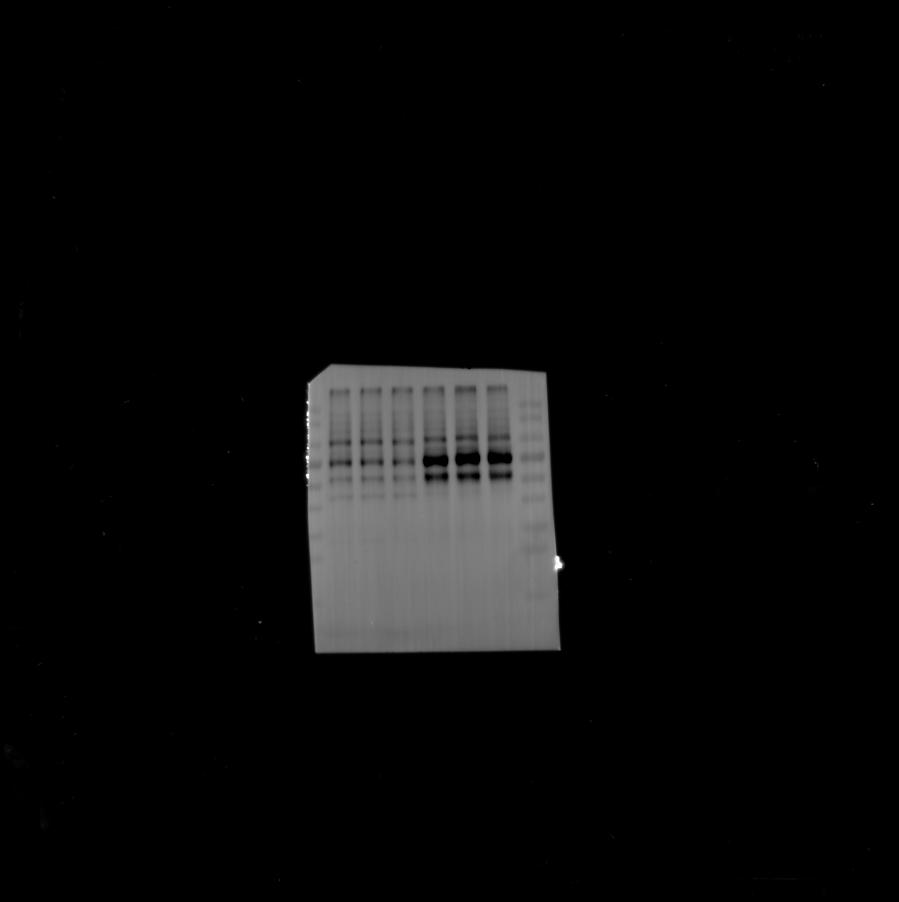

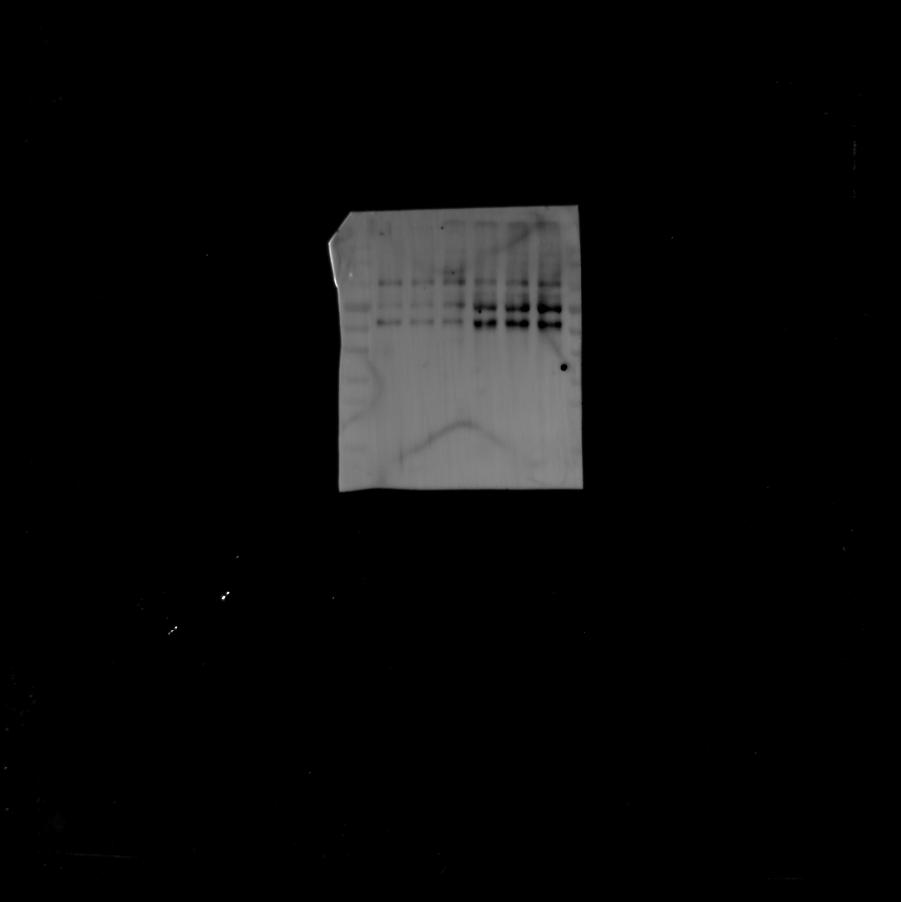
**

**75kda**

**55kda**

**43kda**

**SPHK1**

**SPHK1**

**
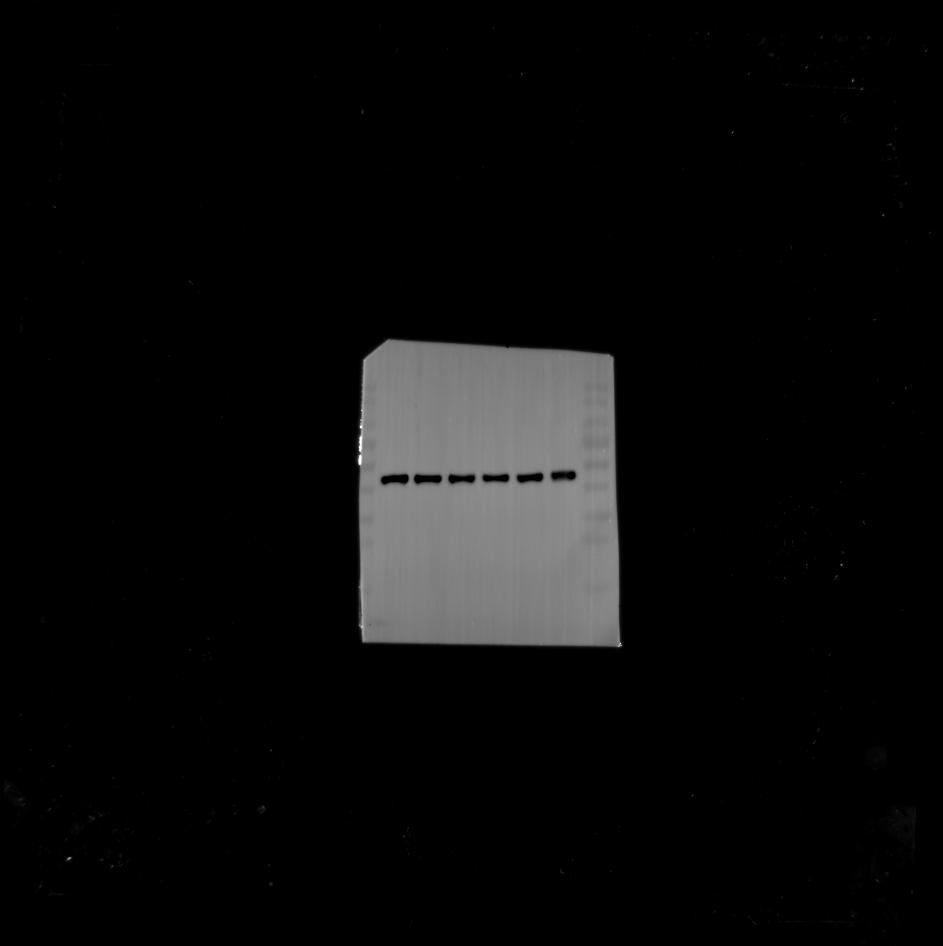

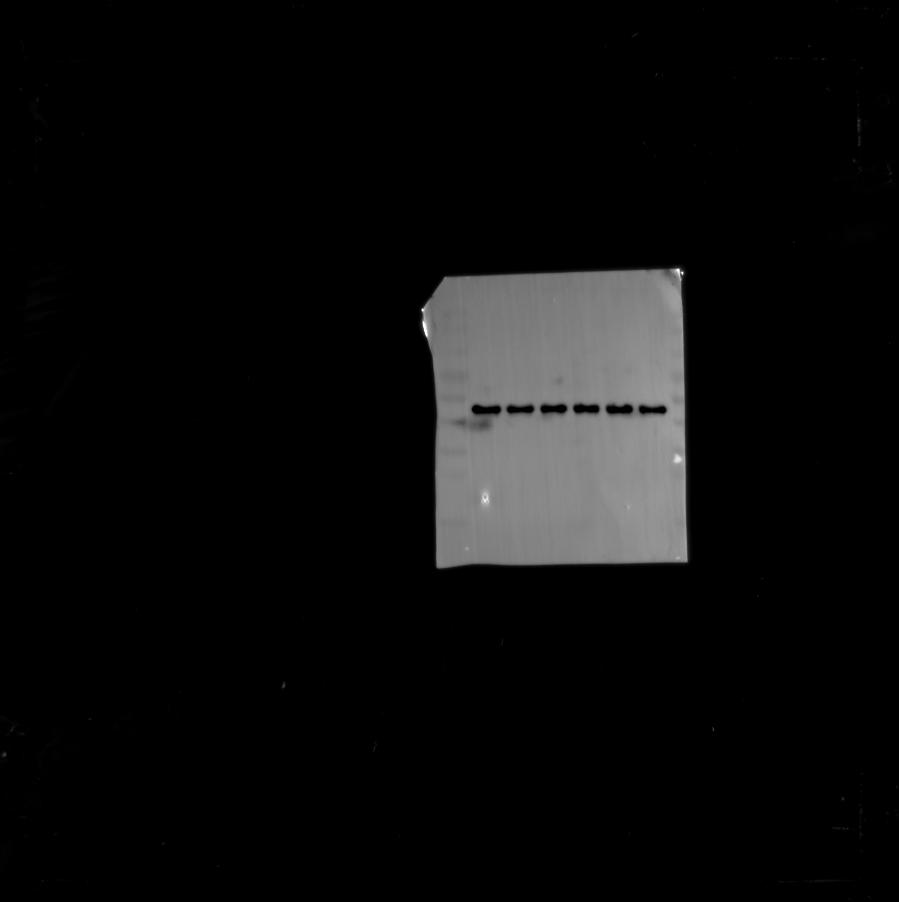
**

**75kda**

**55kda**

**β-actin**

**43kda**

**β-actin**

**Uncropped Gels and Blots image(s) in Figure 3C**

**
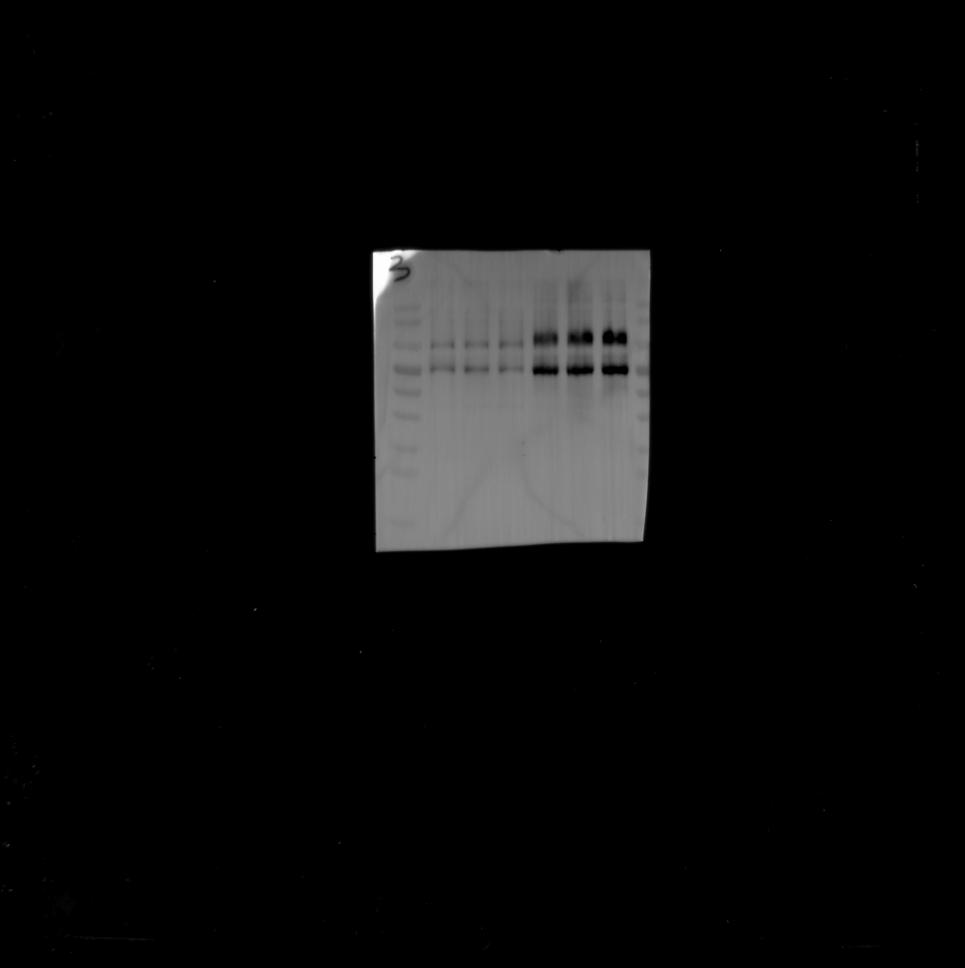
**



**130kda**

**95kda**

**75kda**

**CD68**

**CD68**

**
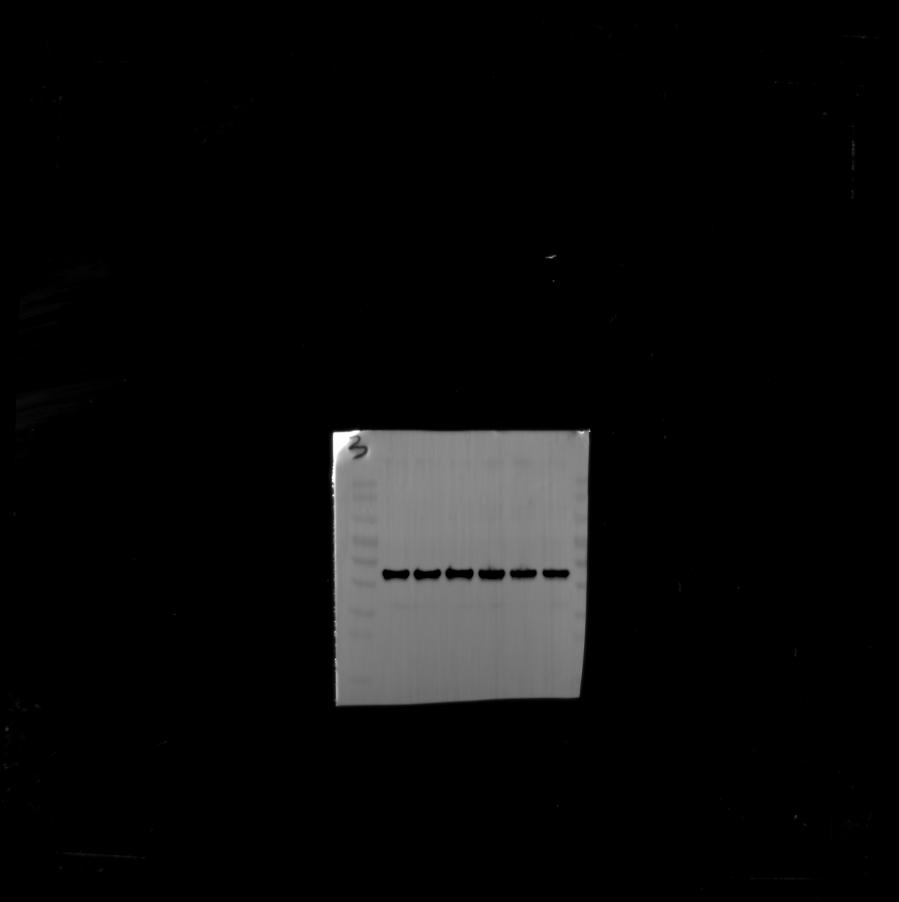

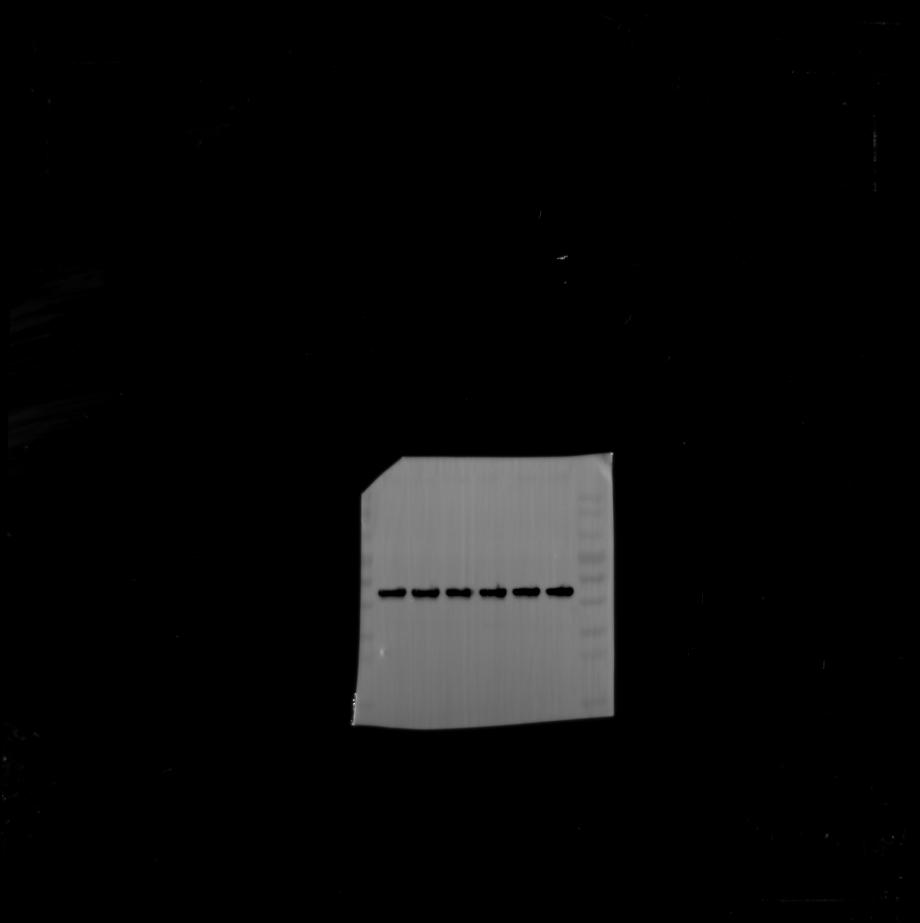
**

**75kda**

**43kda**

**55kda**

**β-actin**

**β-actin**

**
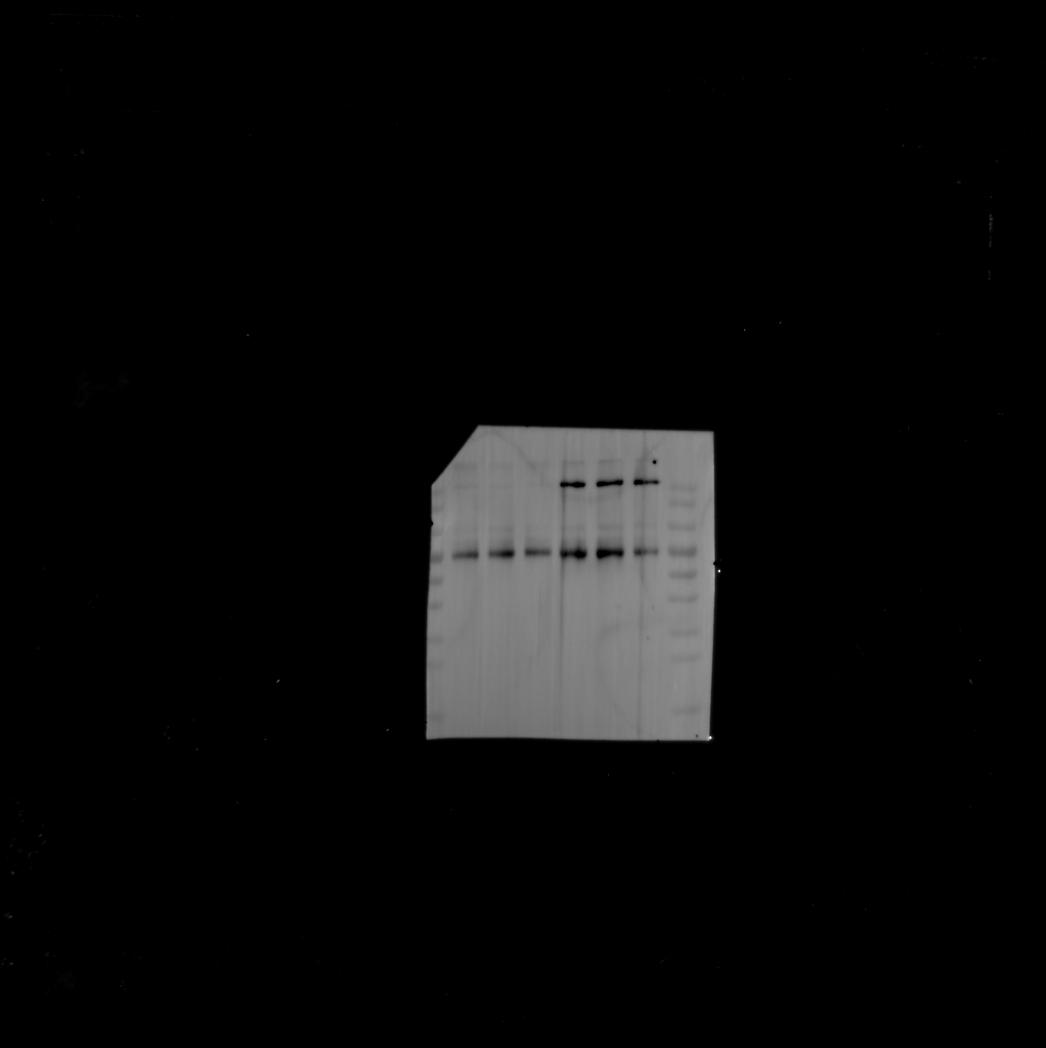

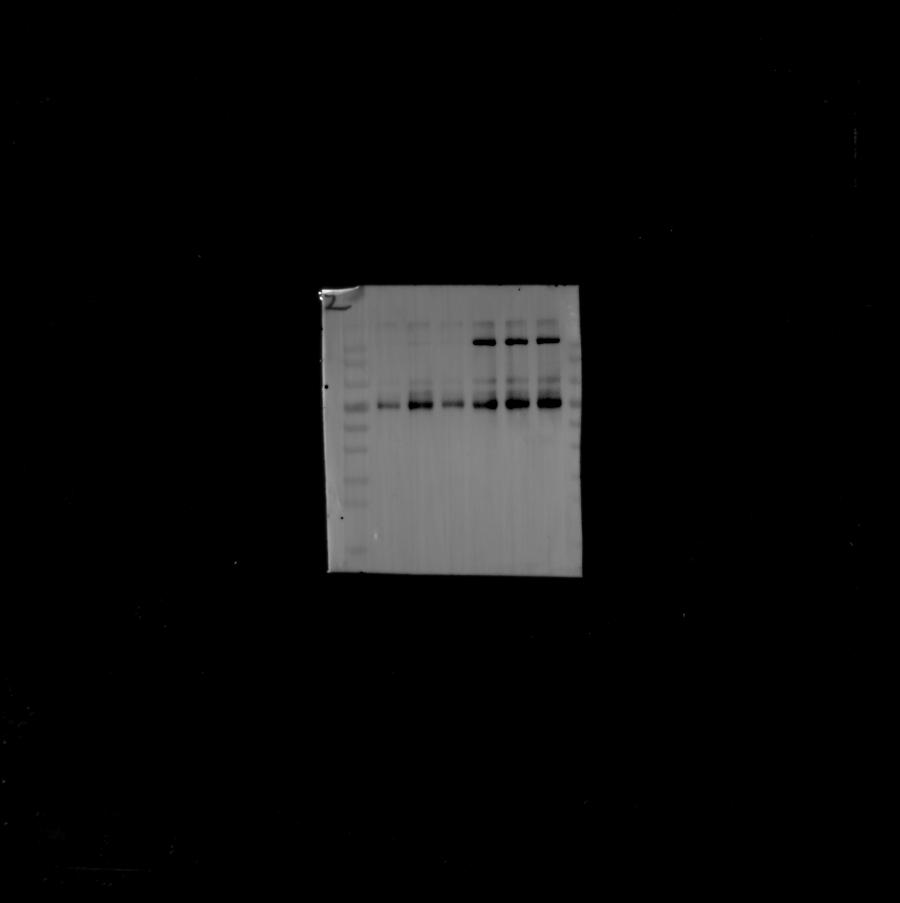
**

**180kda**

**130kda**

**95kda**

**CD206**

**CD206**

**
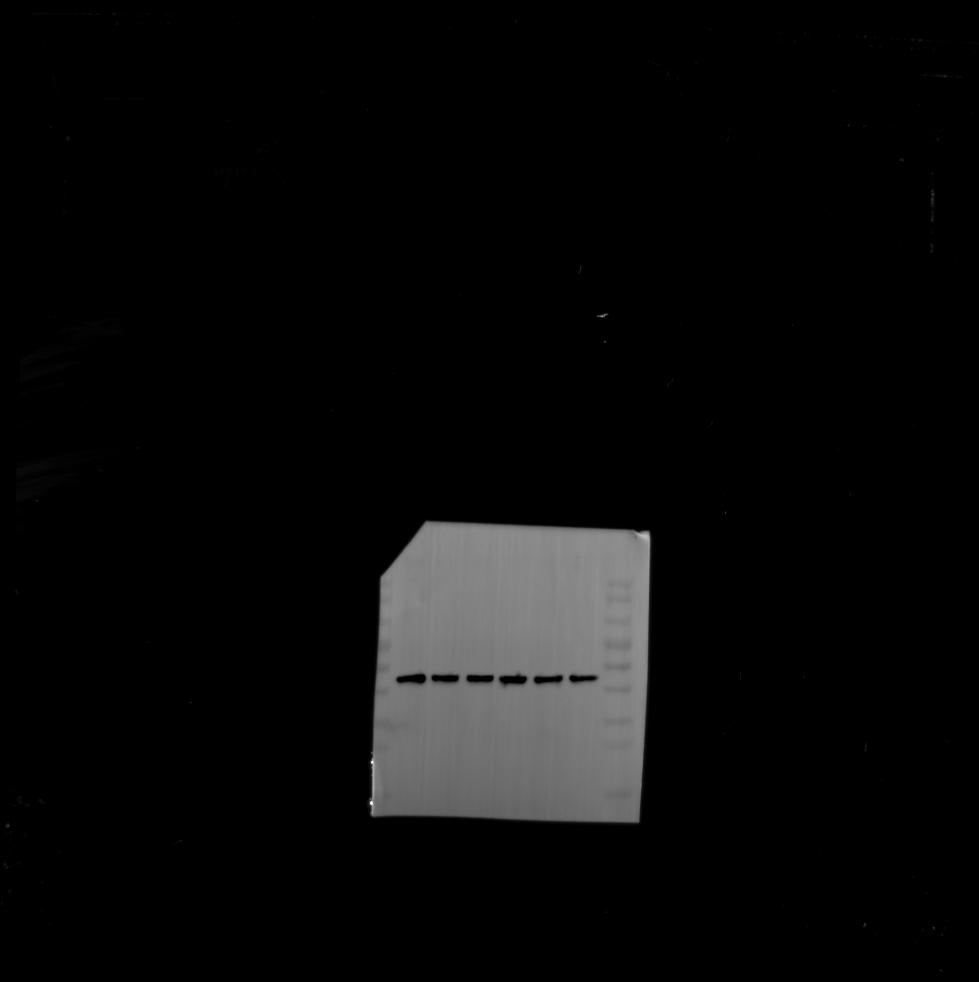

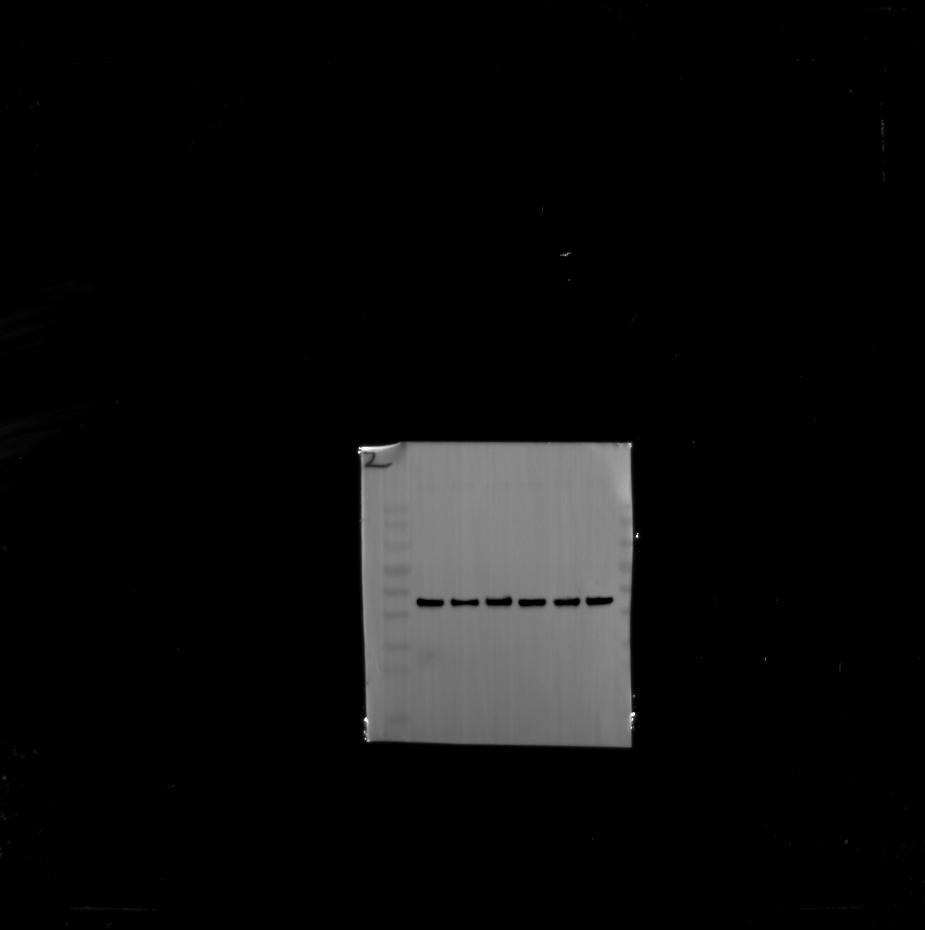
**

**55kda**

**43kda**

**β-actin**

**β-actin**



**
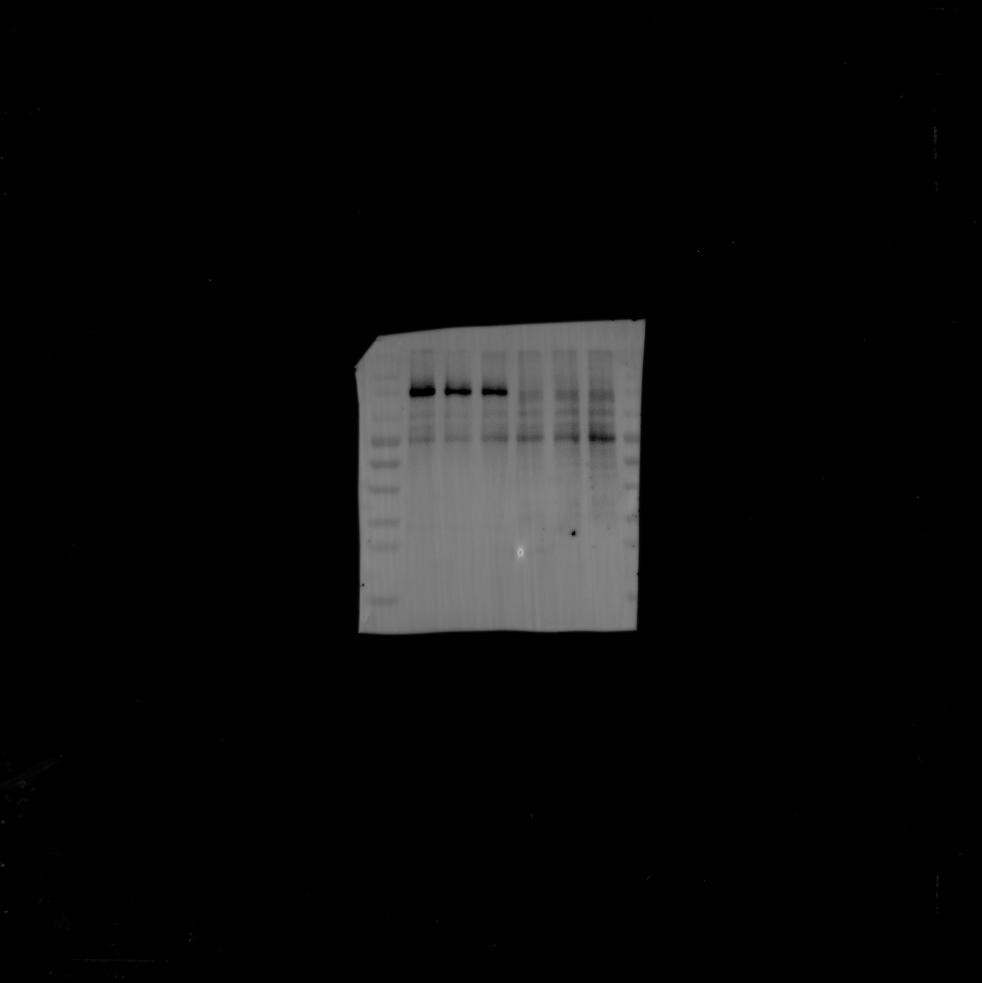
**

**130kda**

**95kda**

**inos**

**inos**

**
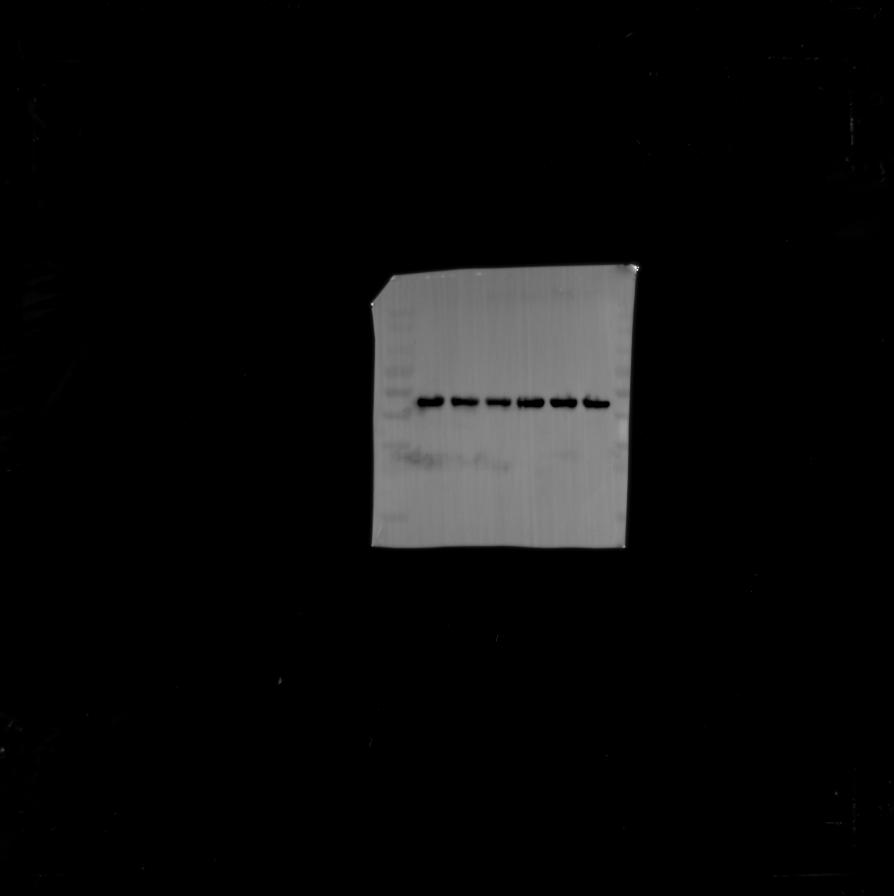
**



**55kda**

**43kda**

**β-actin**

**β-actin**

**Uncropped Gels and Blots image(s) in Figure 4K**

**
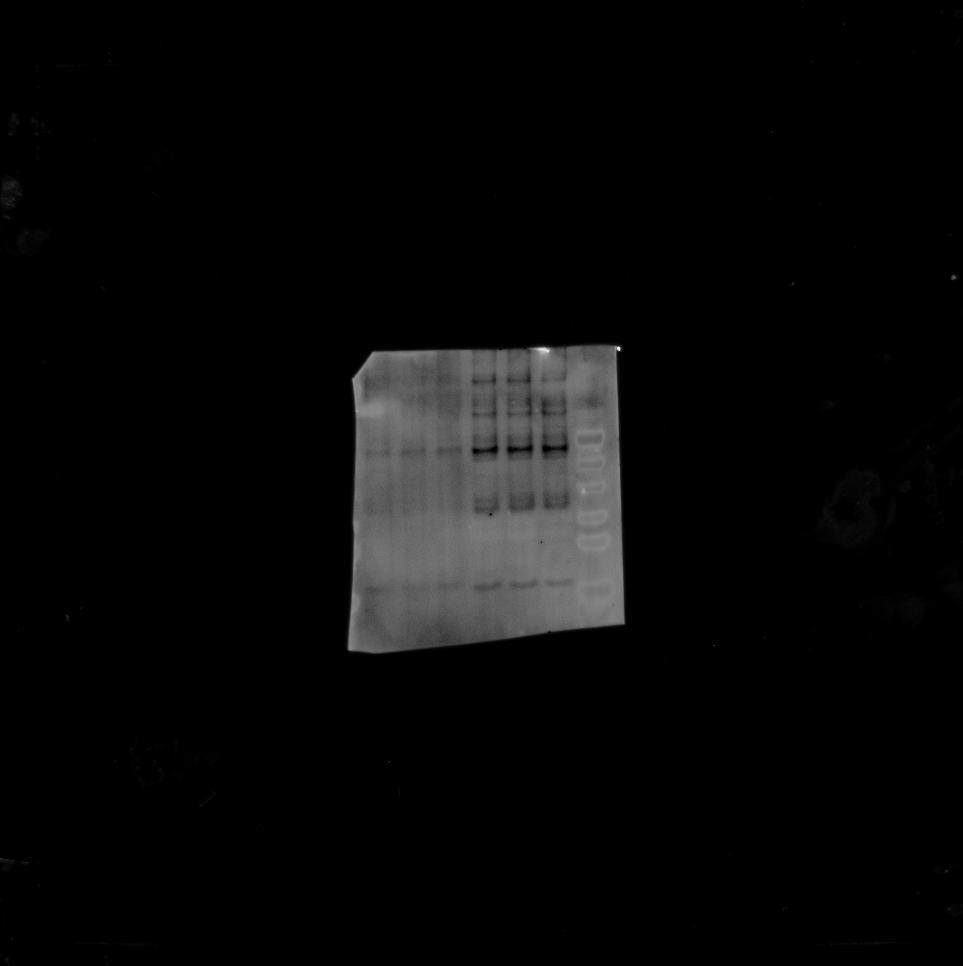

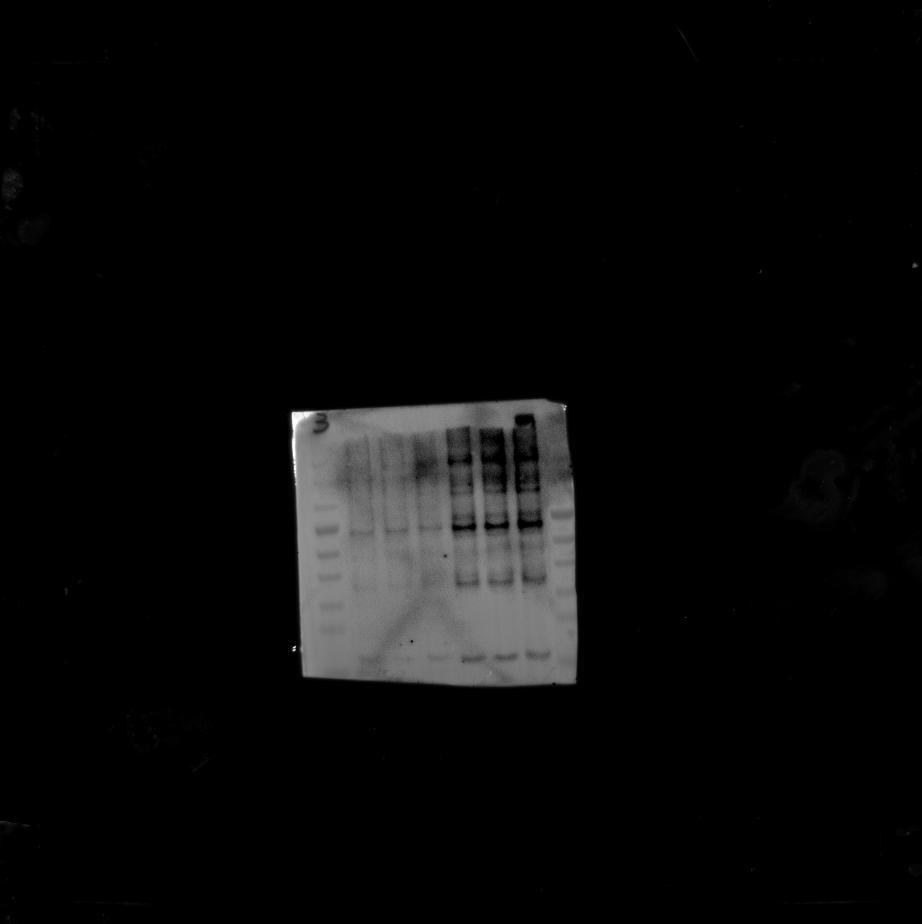
**

**75kda**

**55kda**

**SPHK1**

**SPHK1**


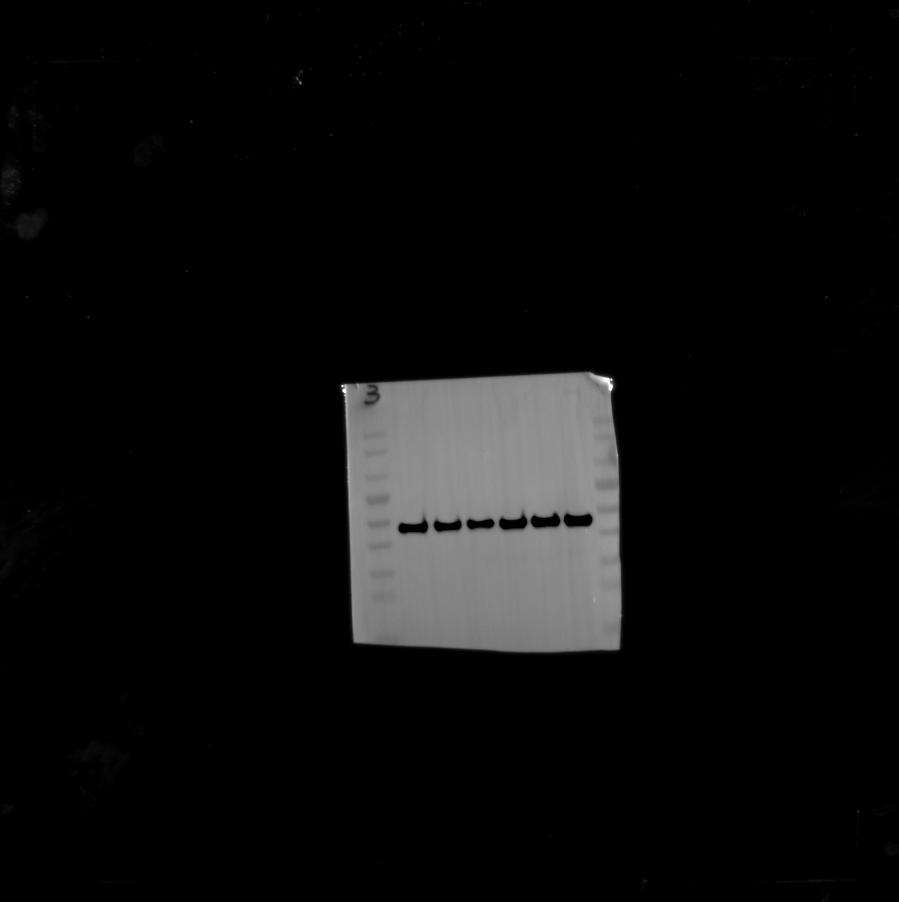
**
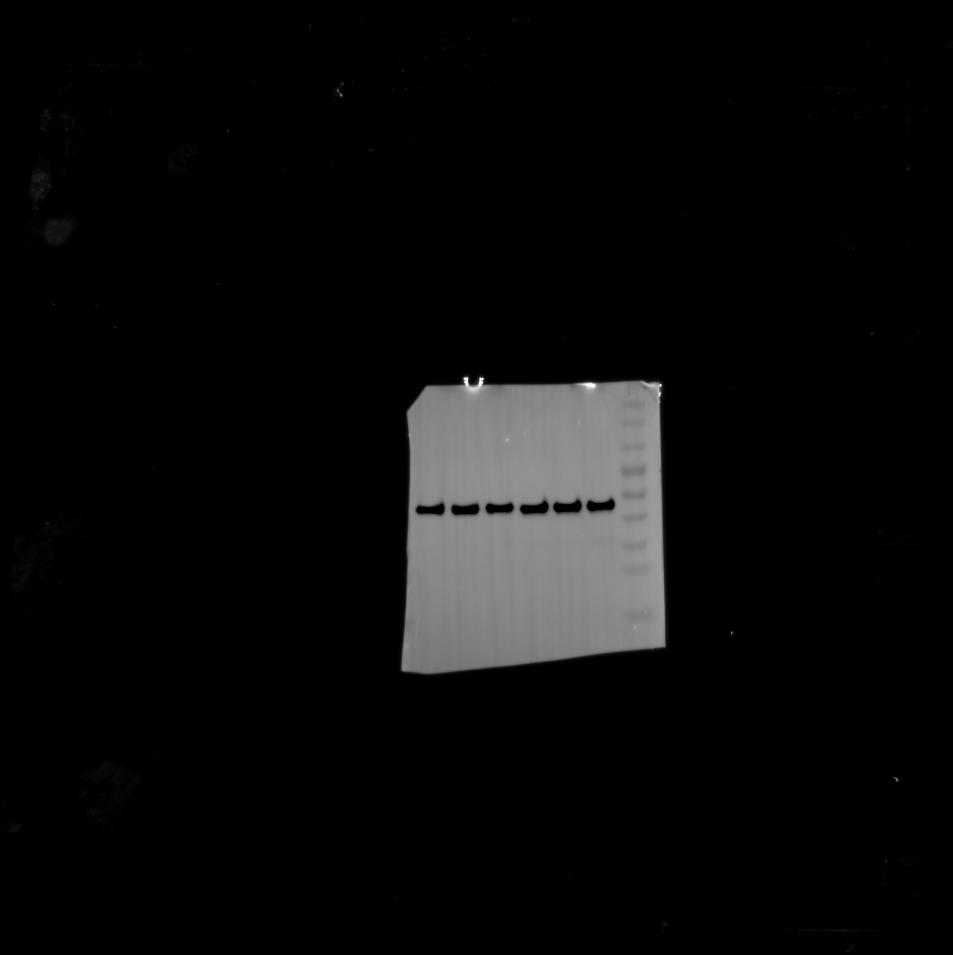
**

**55kda**

**43kda**

**β-actin**

**β-actin**

**Uncropped Gels and Blots image(s) in Figure 4M**

**
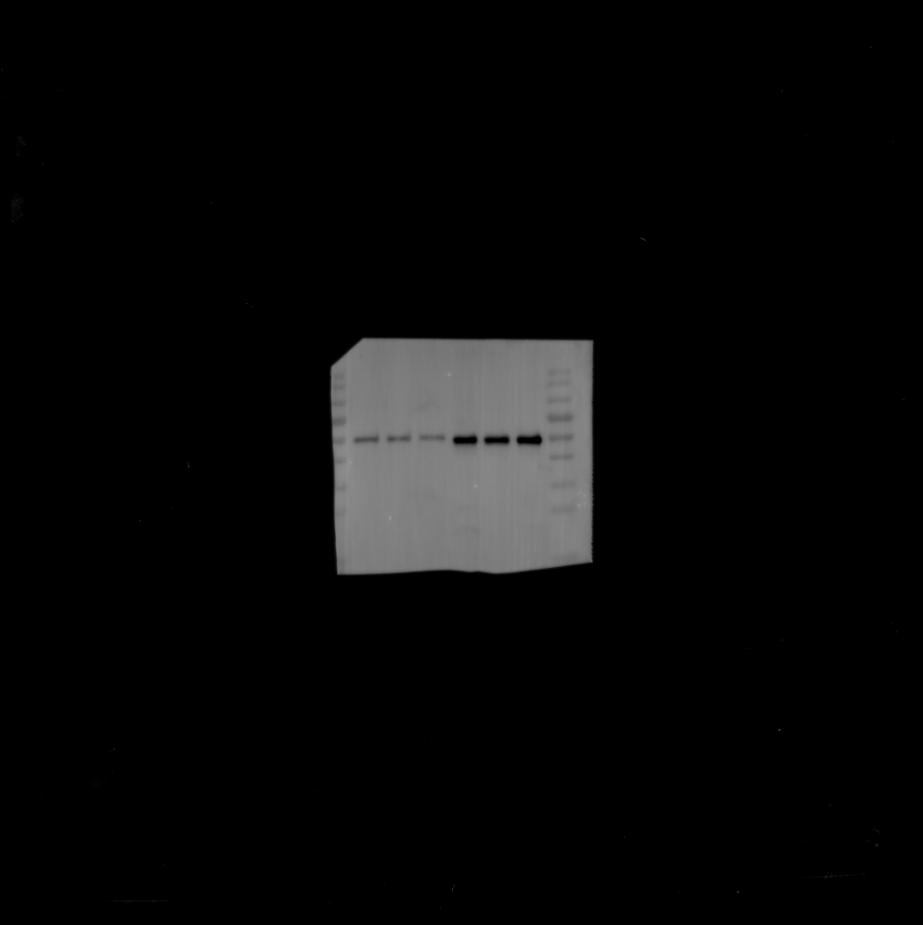

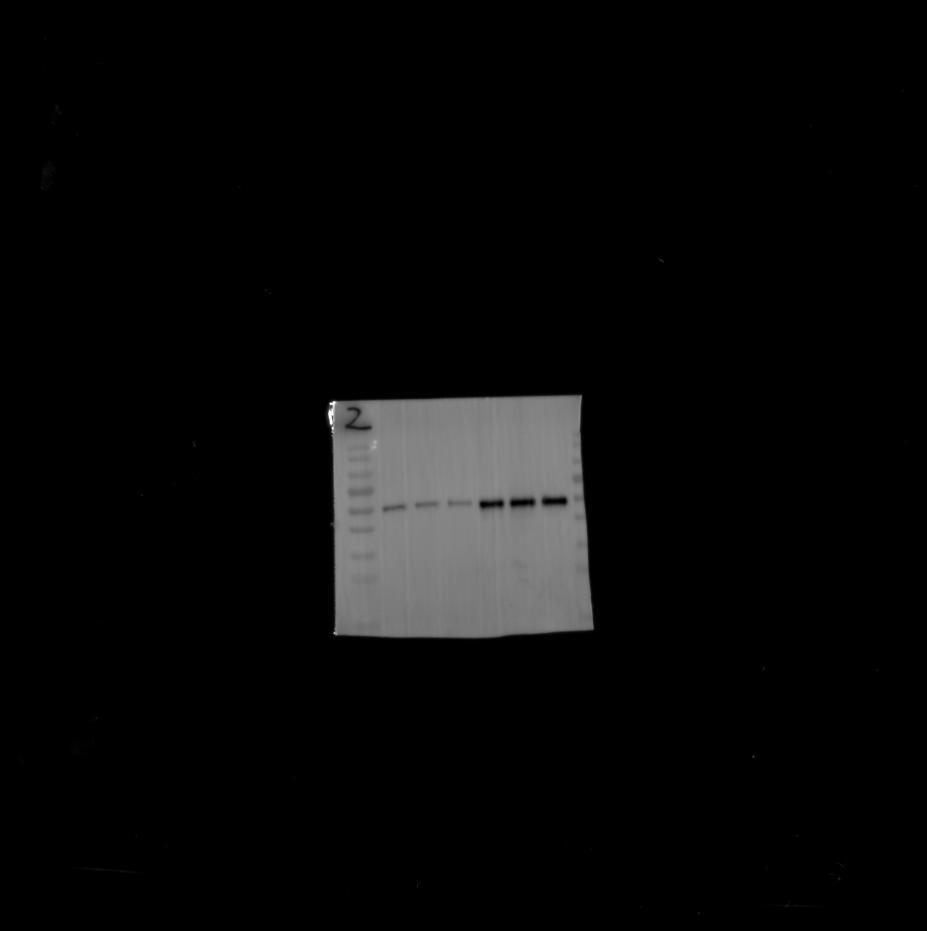
**

**75kda**

**55kda**

**43kda**

**TGFB-1**

**TGFB-1**

**
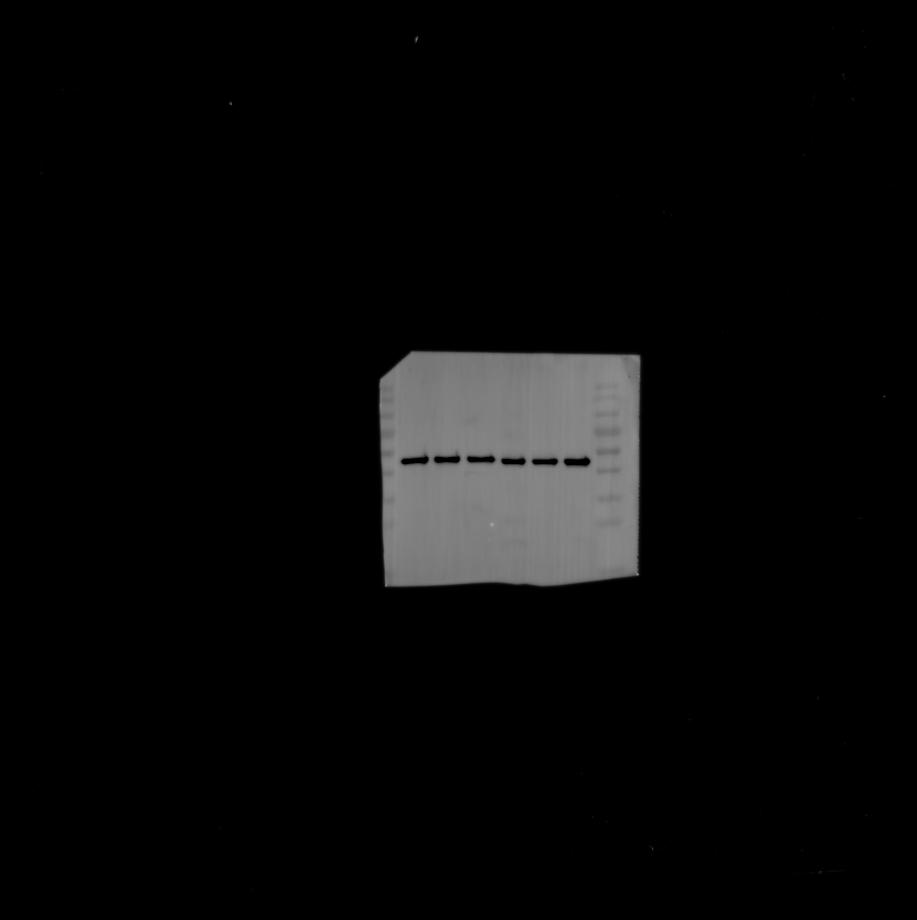

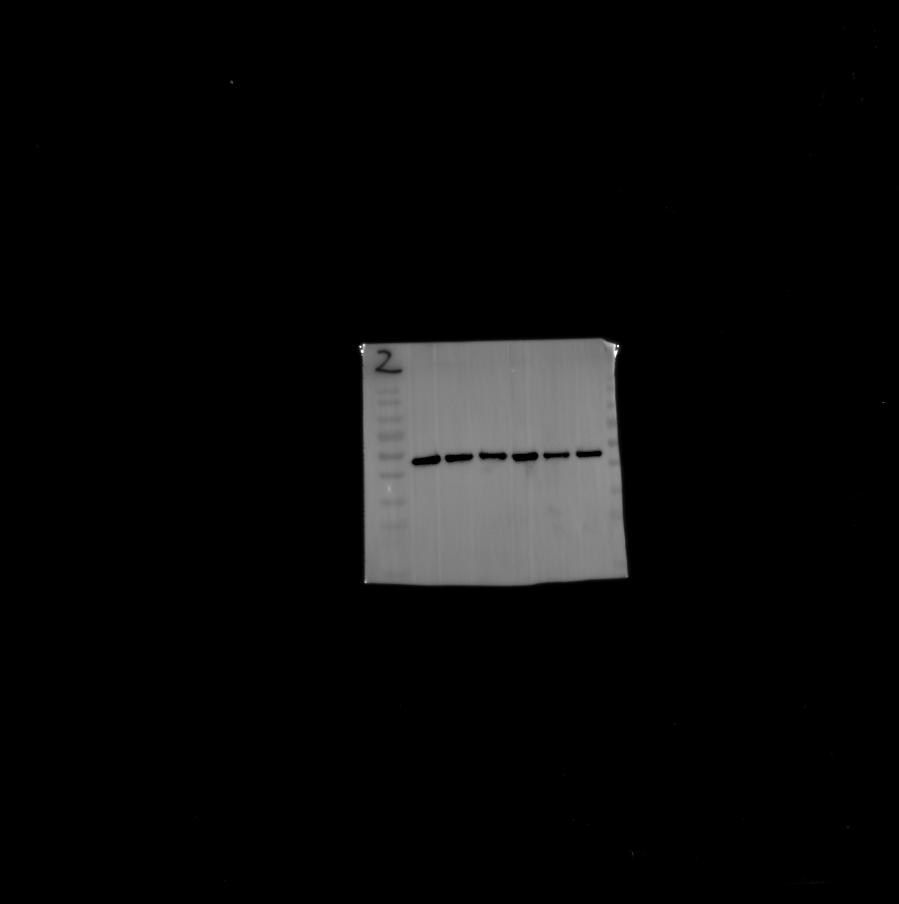
**

**55kda**

**43kda**

**β-actin**

**β-actin**

**
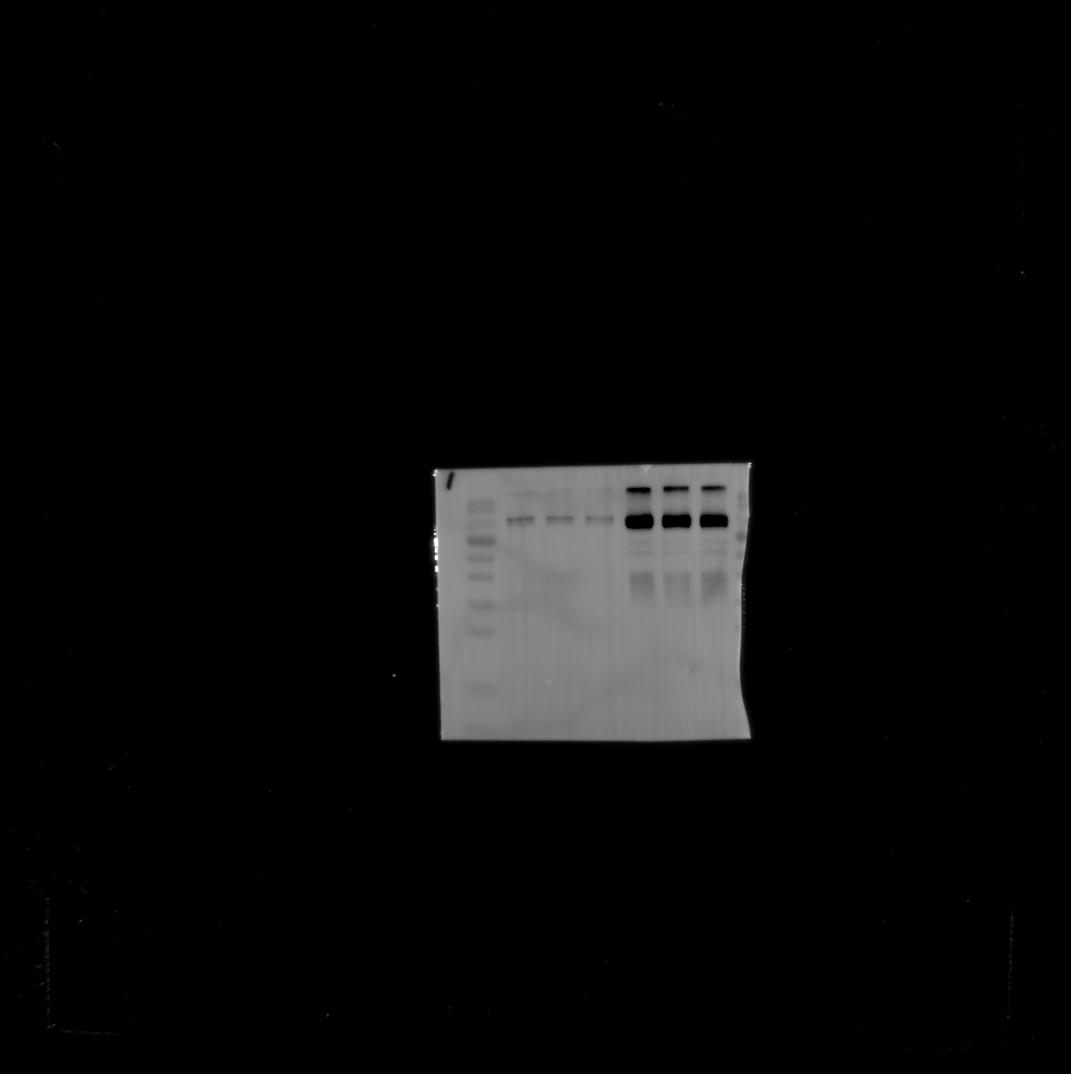
**





**95kda**

**75kda**

**MMP9**

**MMP9**

**
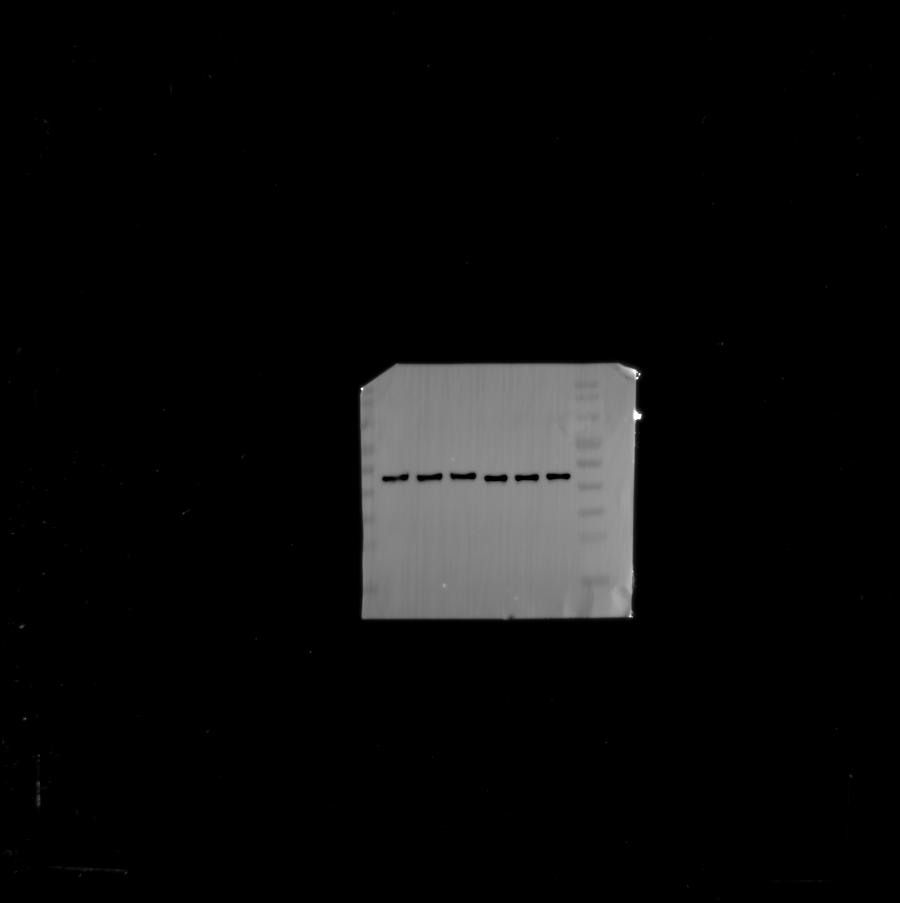

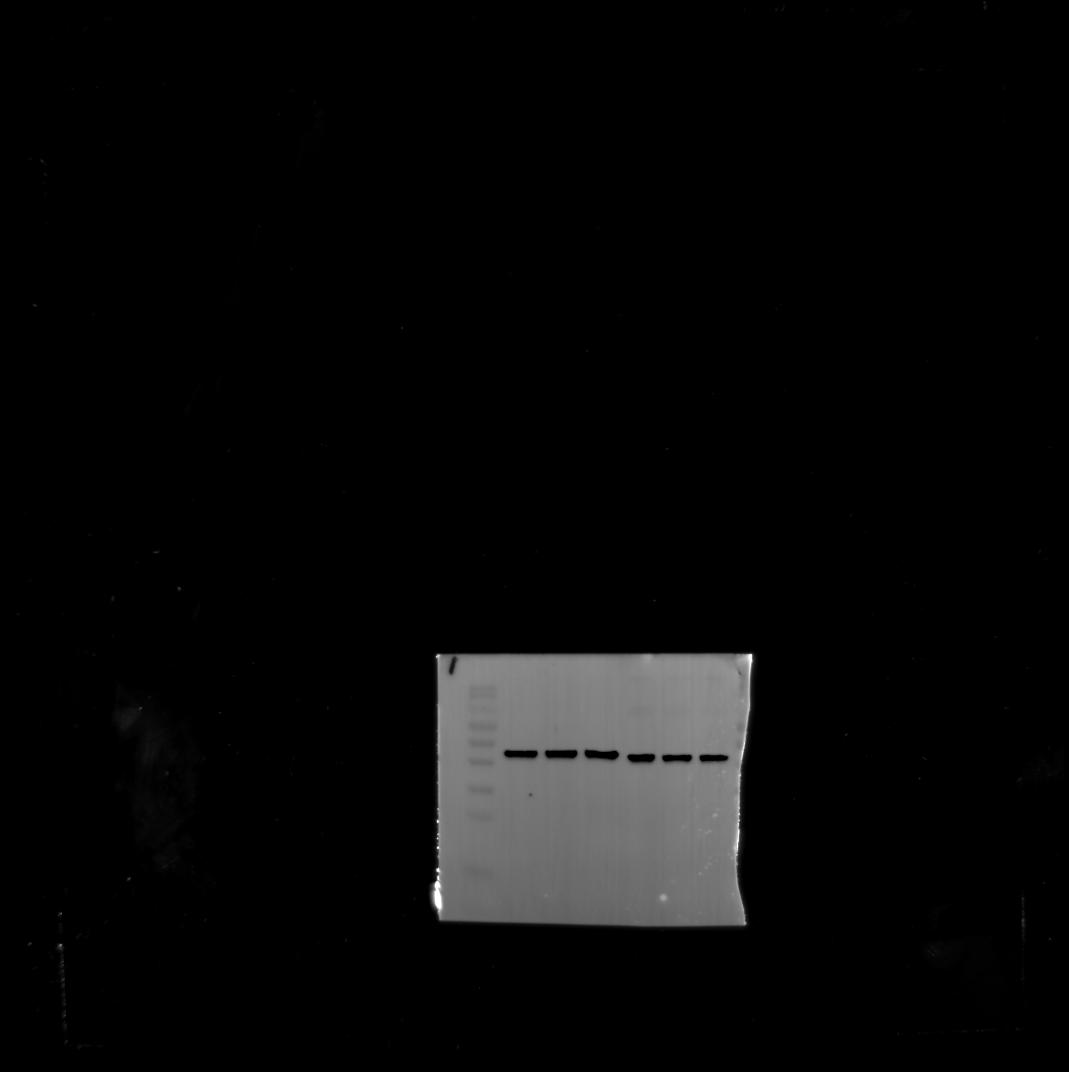
**

**55kda**

**43kda**

**β-actin**

**β-actin**

**Uncropped Gels and Blots image(s) in Figure 4L**

**
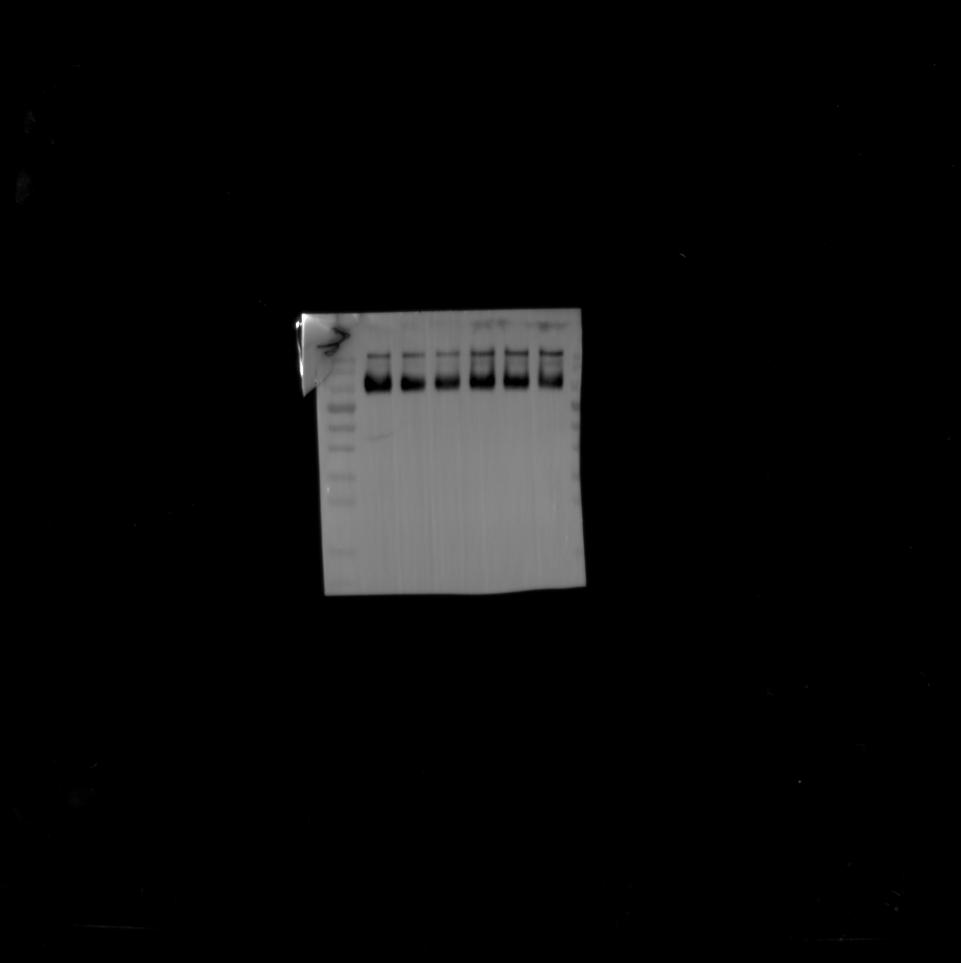

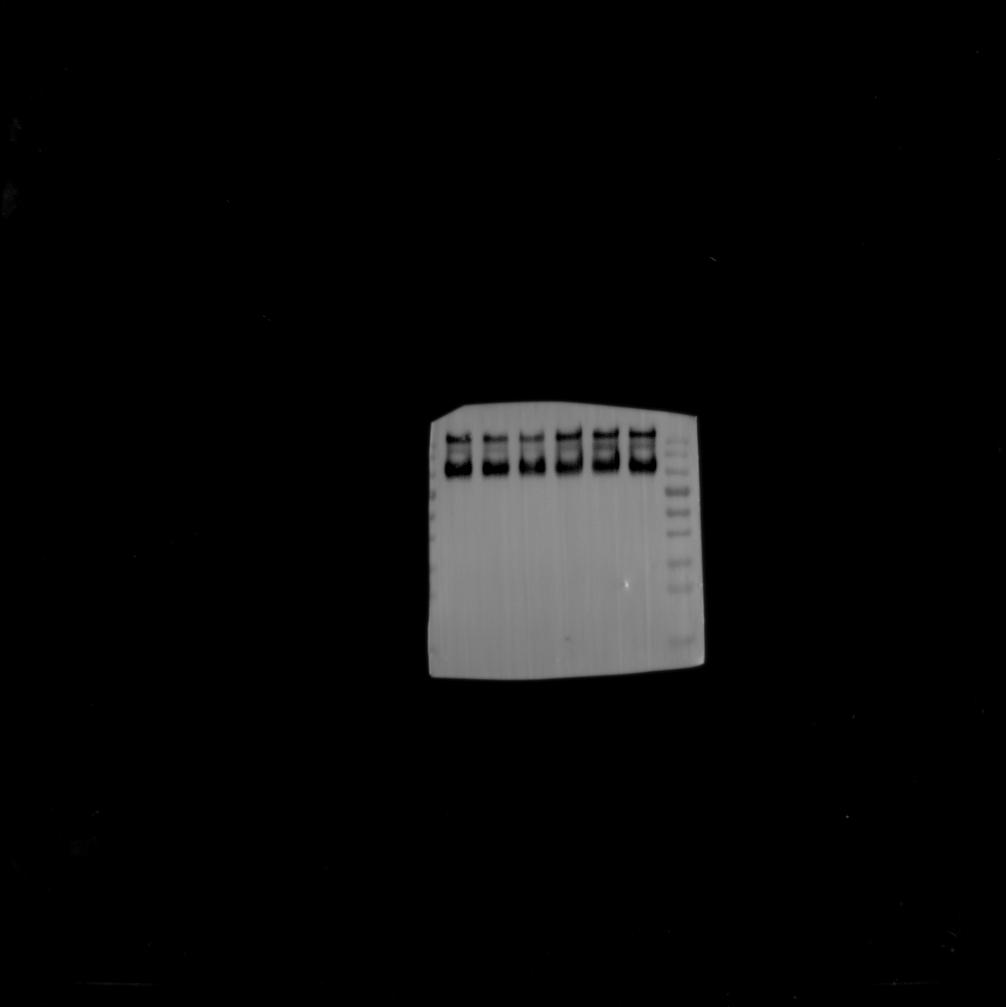
**

**130kda**

**95kda**

**75kda**

**CD68**

**CD68**

**
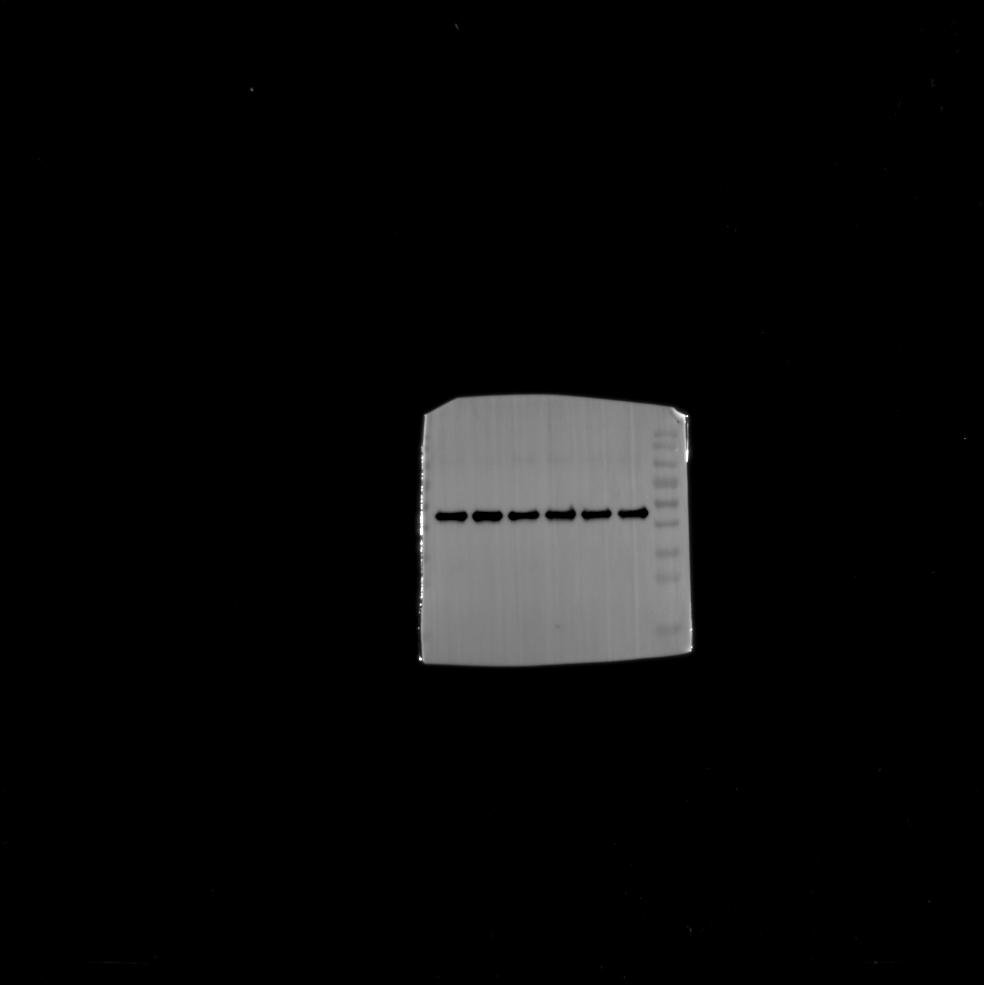

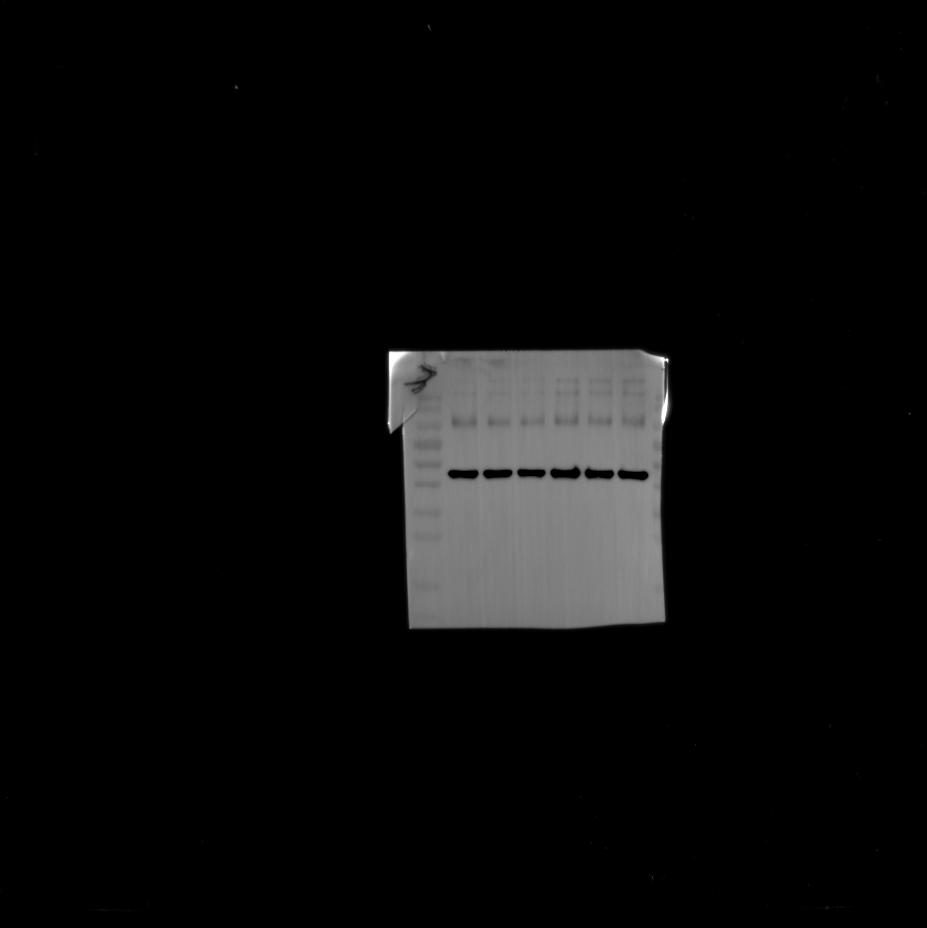
**

**55kda**

**43kda**

**β-actin**

**β-actin**

**
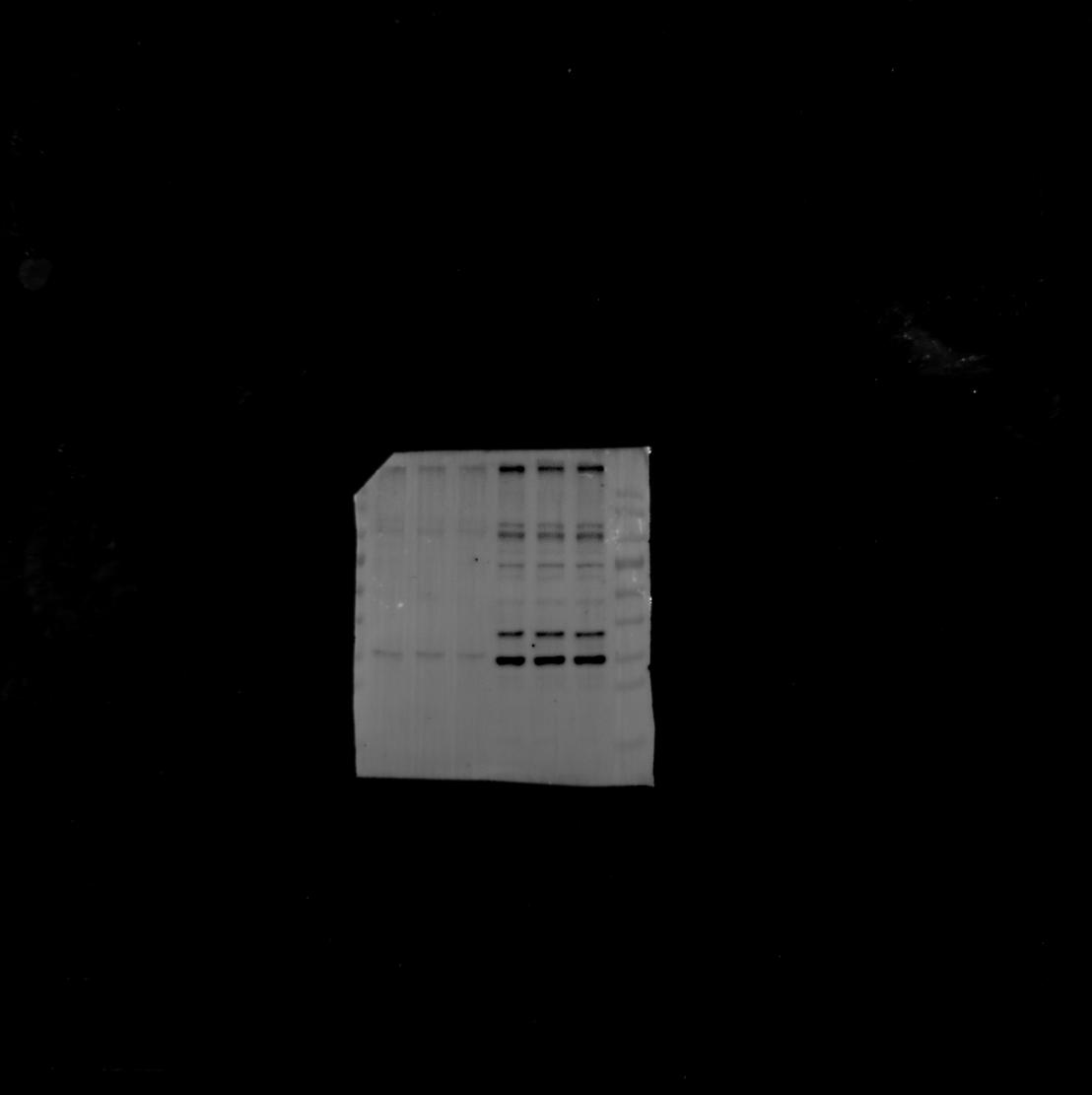

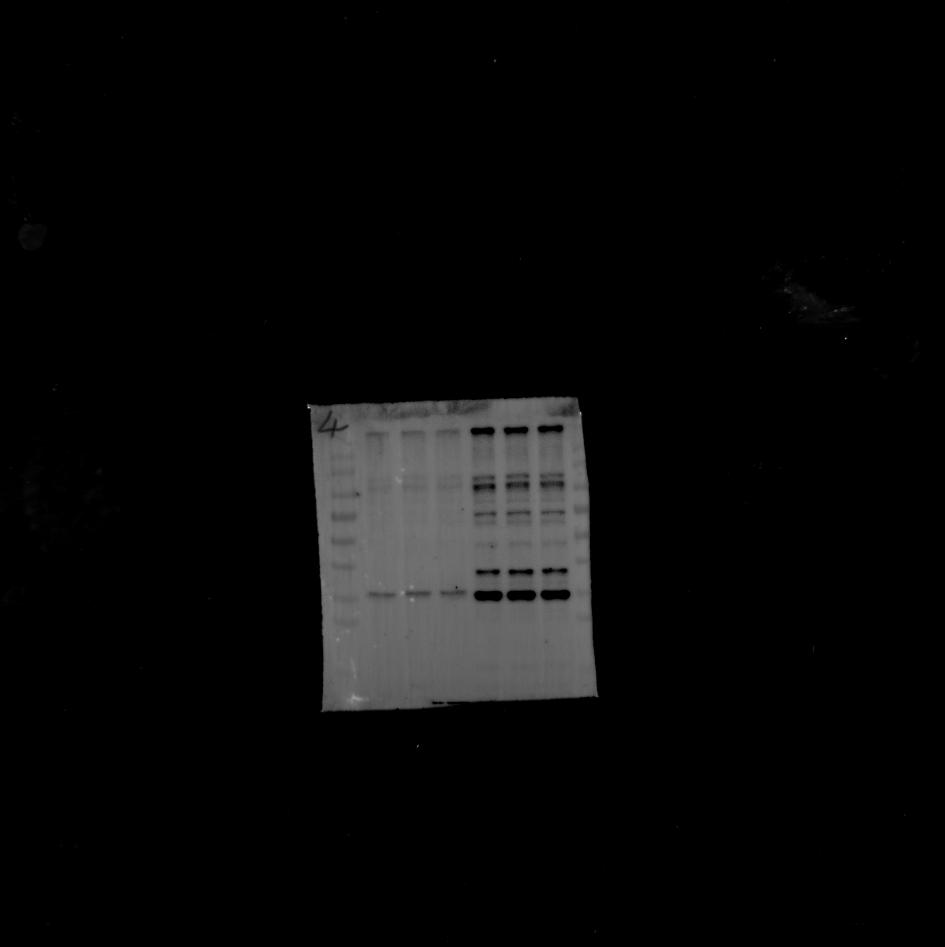
**

**55kda**

**43kda**

**33kda**

**Arg-1**

**Arg-1**

**
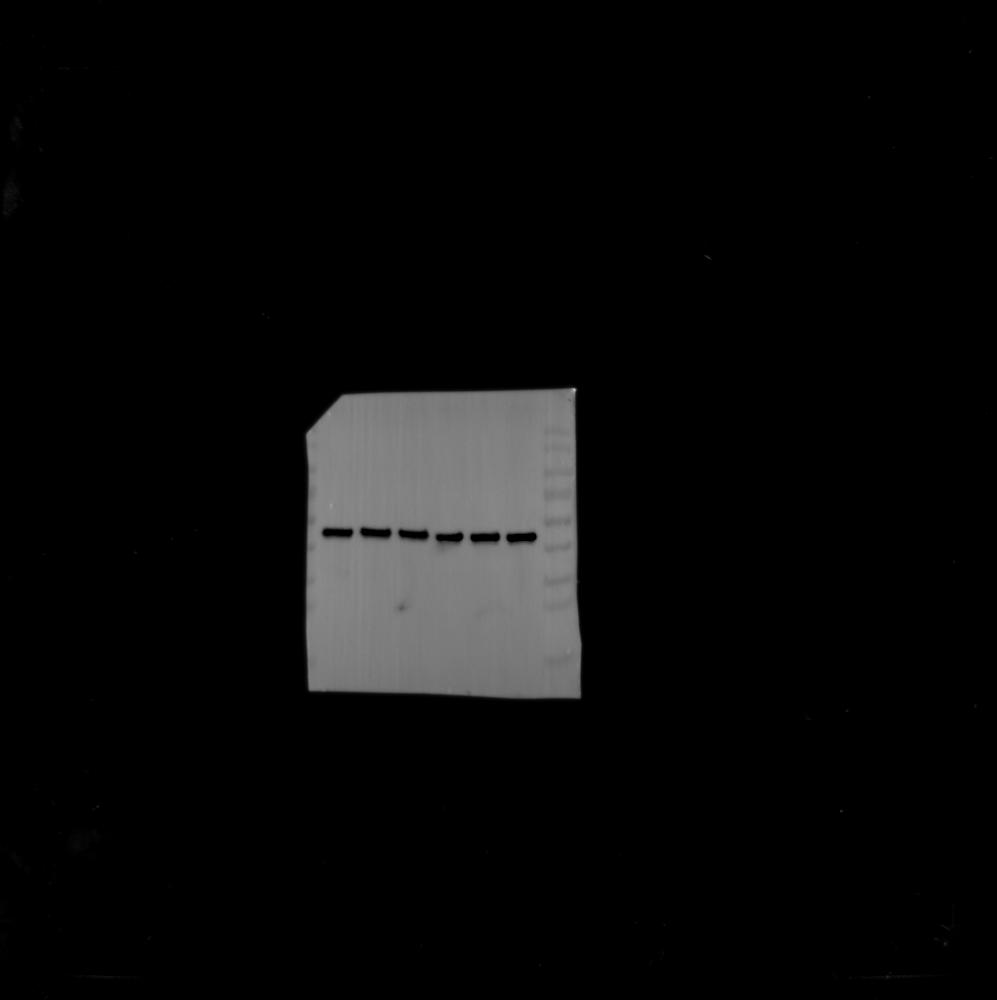

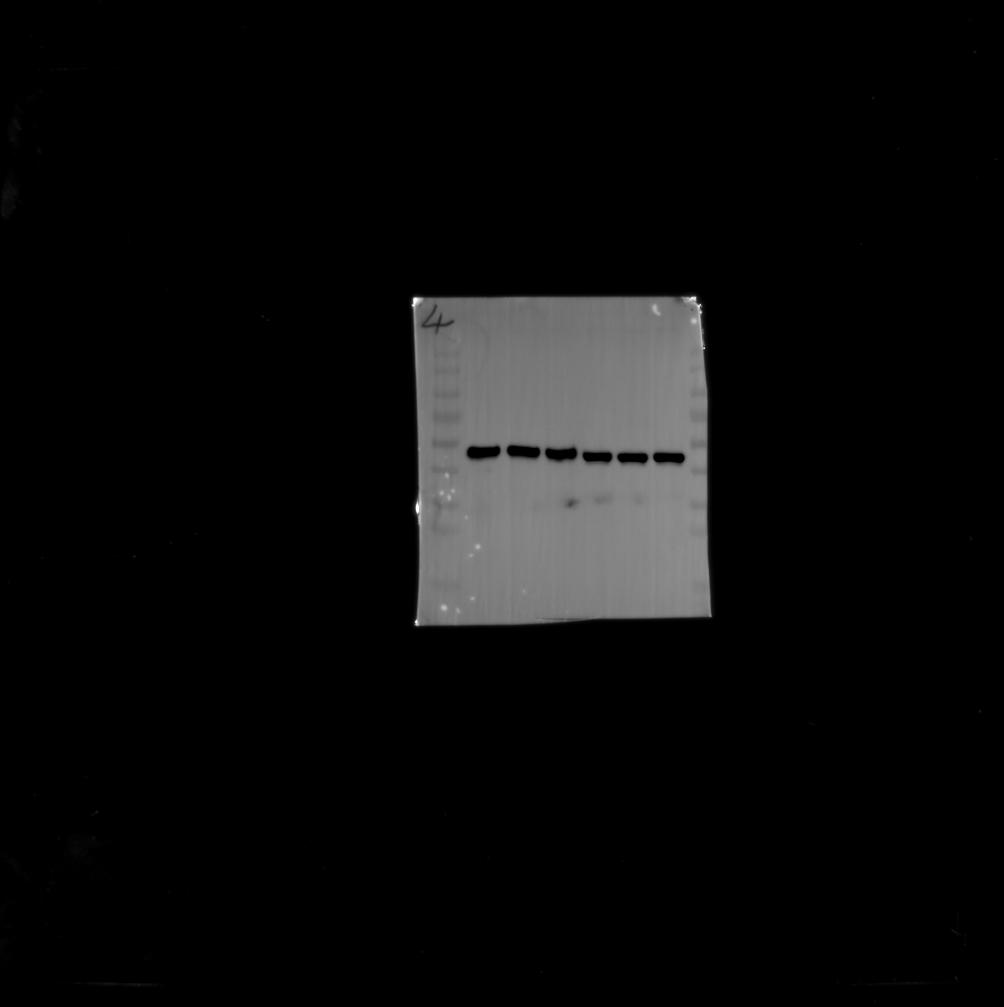
**

**55kda**

**43kda**

**β-actin**

**β-actin**

**
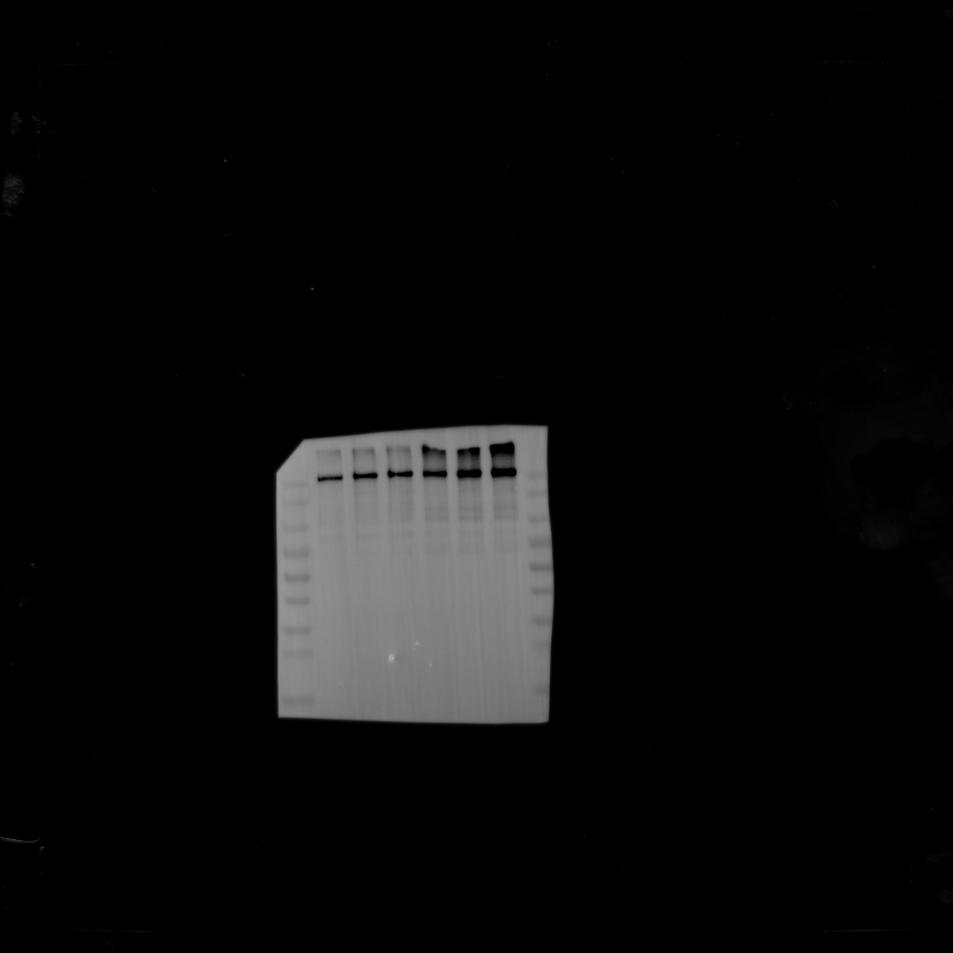

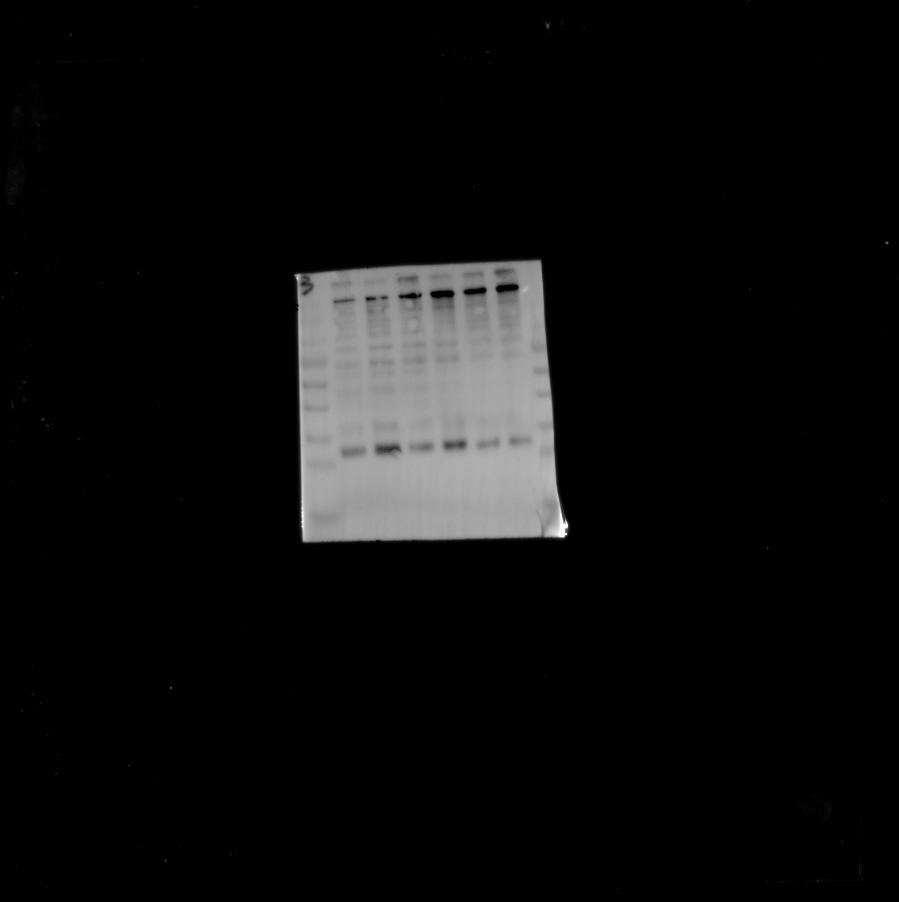
**

**180kda**

**130kda**

v

v

**CD206**

**CD206**

**
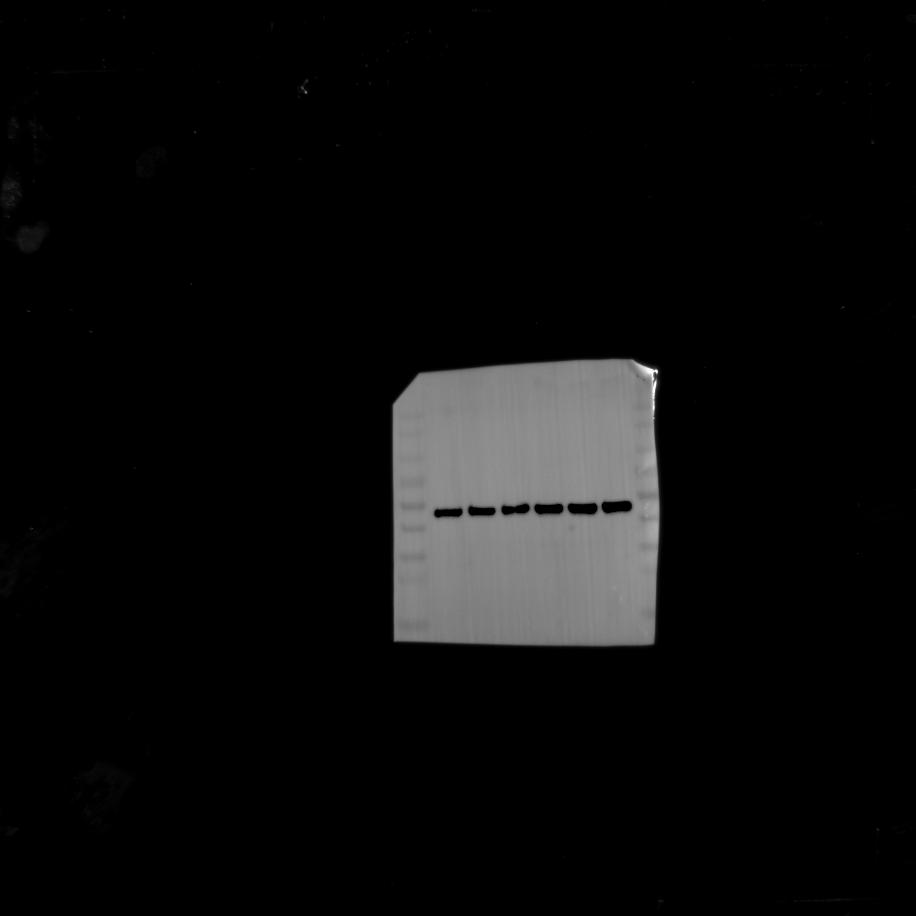

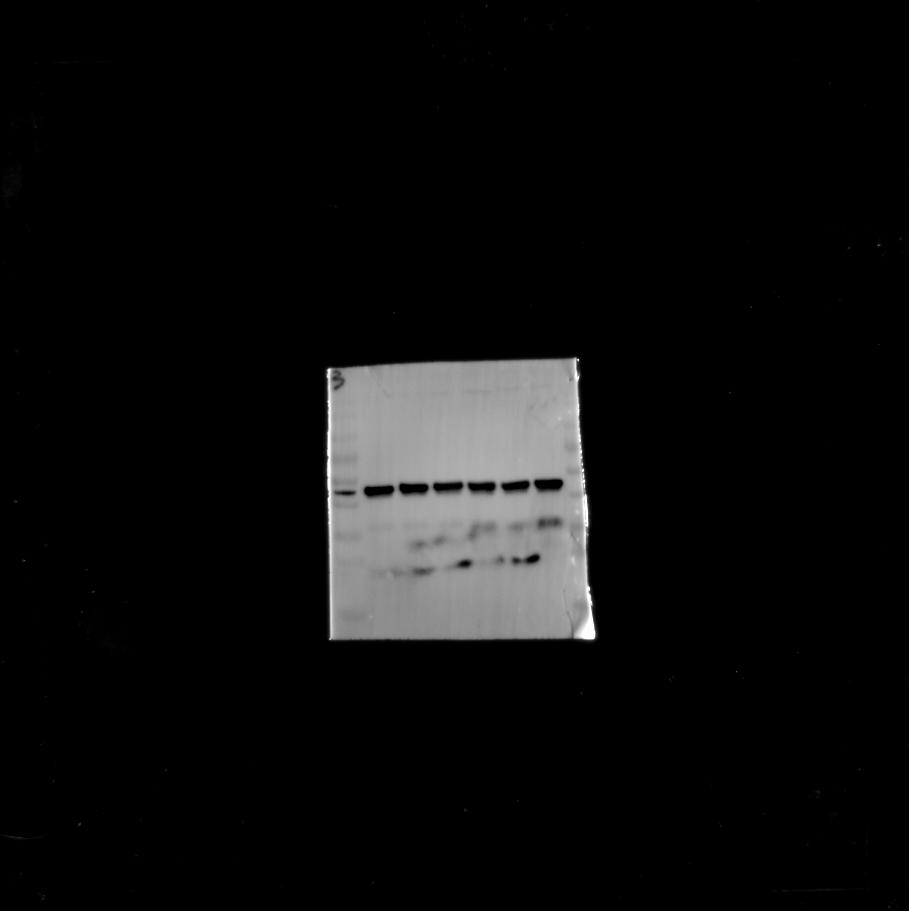
**

**55kda**

**43kda**

**β-actin**

**β-actin**

**Uncropped Gels and Blots image(s) in Figure 5B**

**
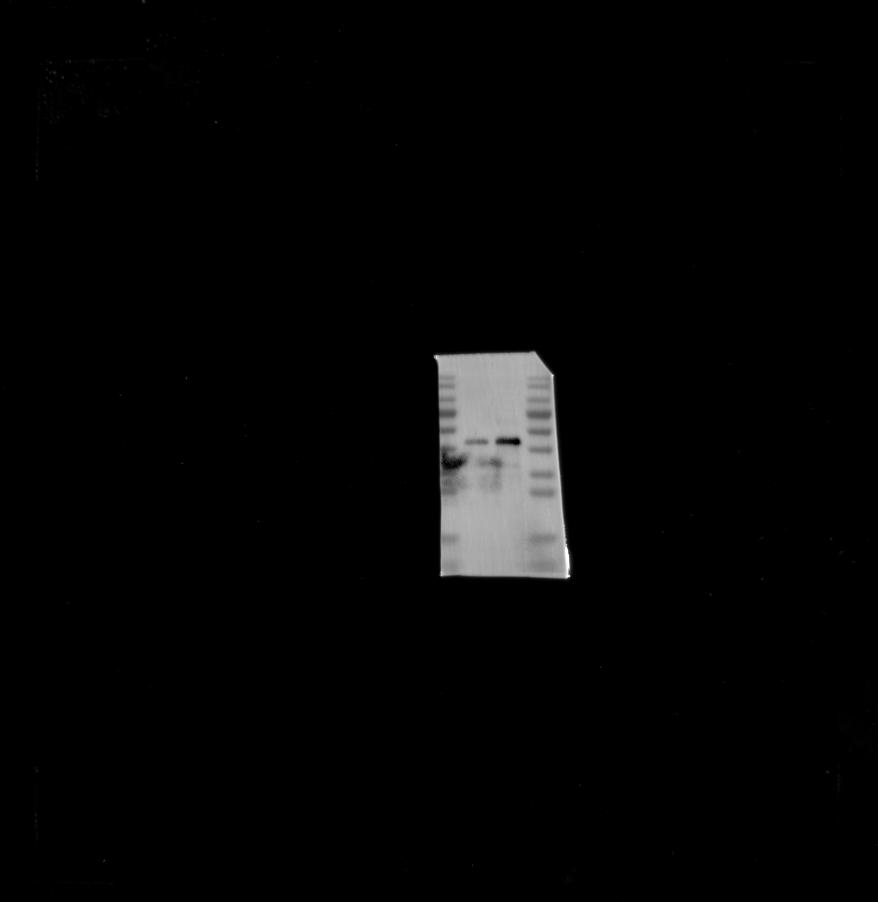

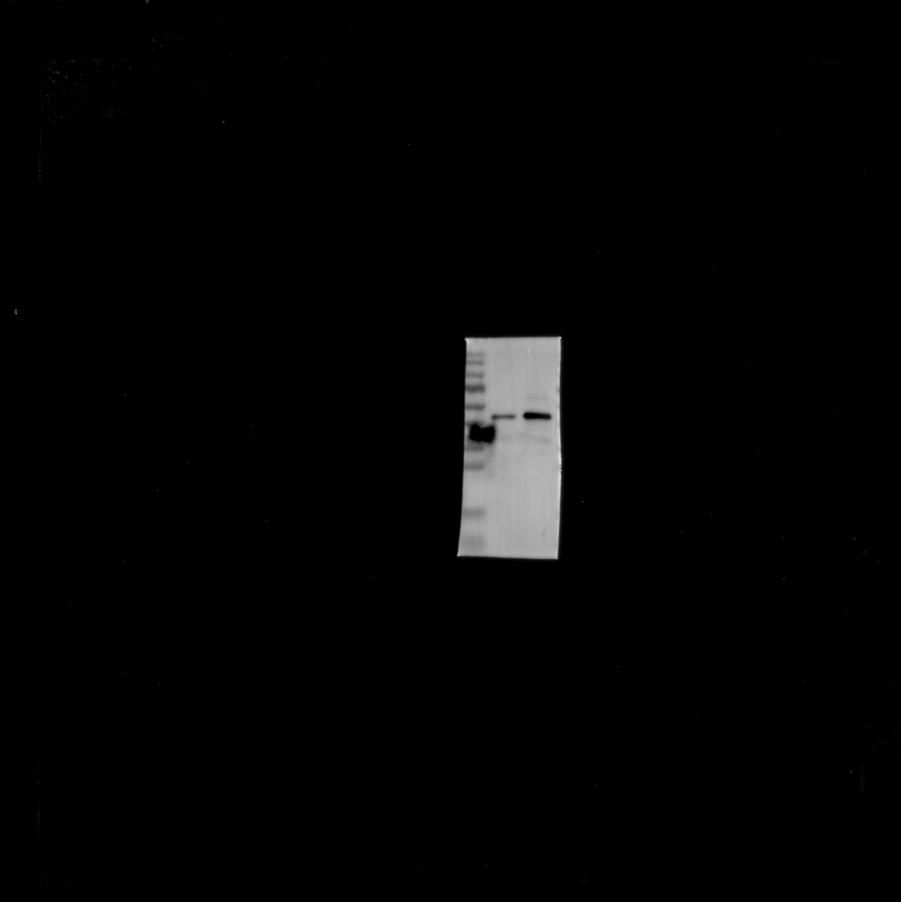

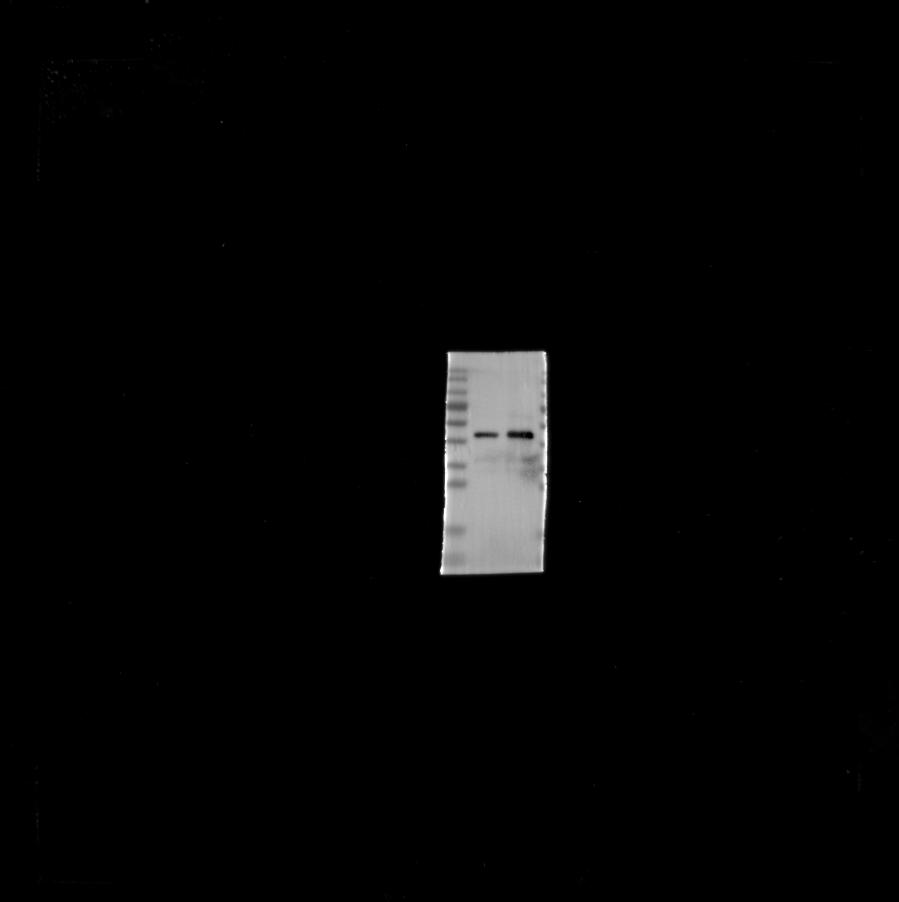
**

**55kda**

**43kda**

**aSMA**

**aSMA**

**aSMA**





**
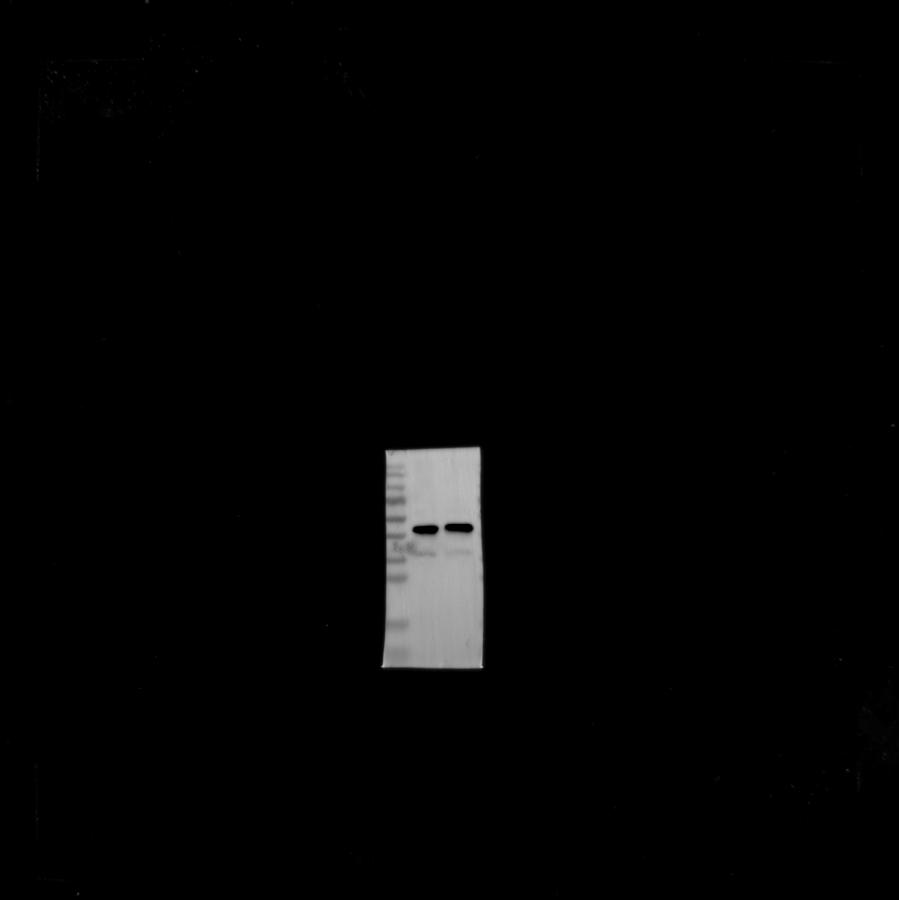
**

**55kda**

**43kda**

**β-actin**

**β-actin**

**β-actin**

**Uncropped Gels and Blots image(s) in Figure 5G
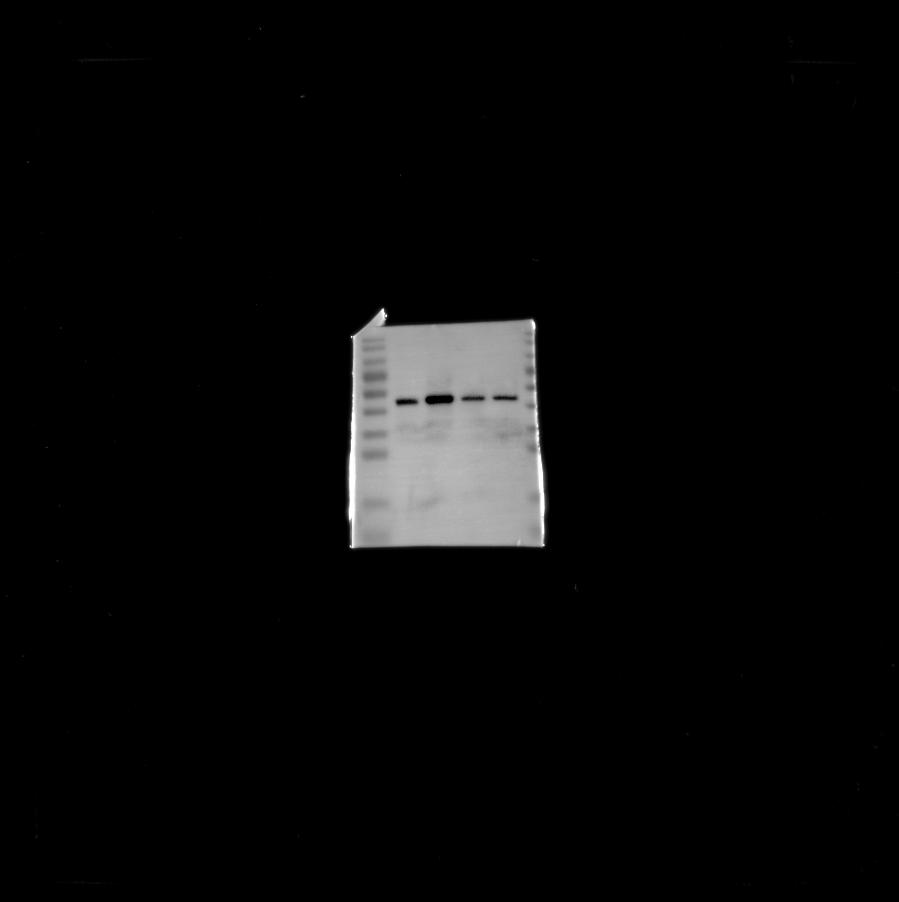
**

**
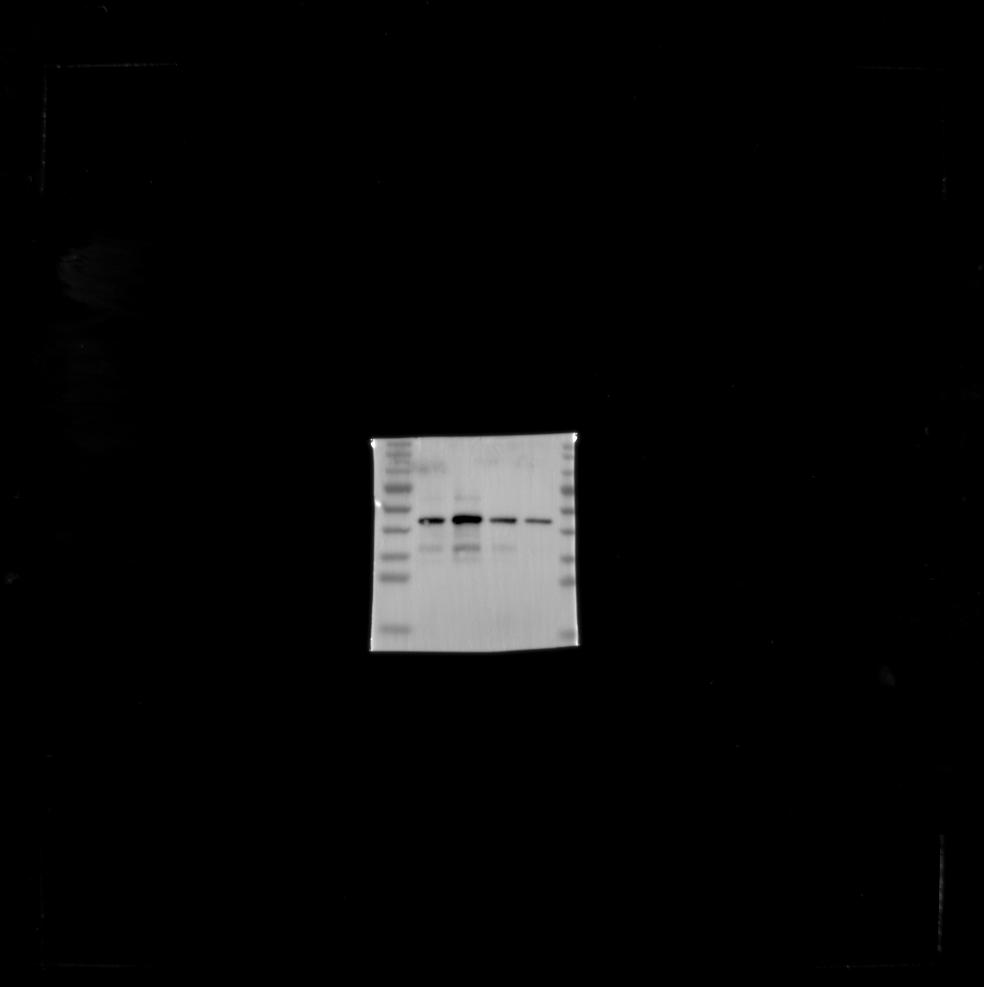

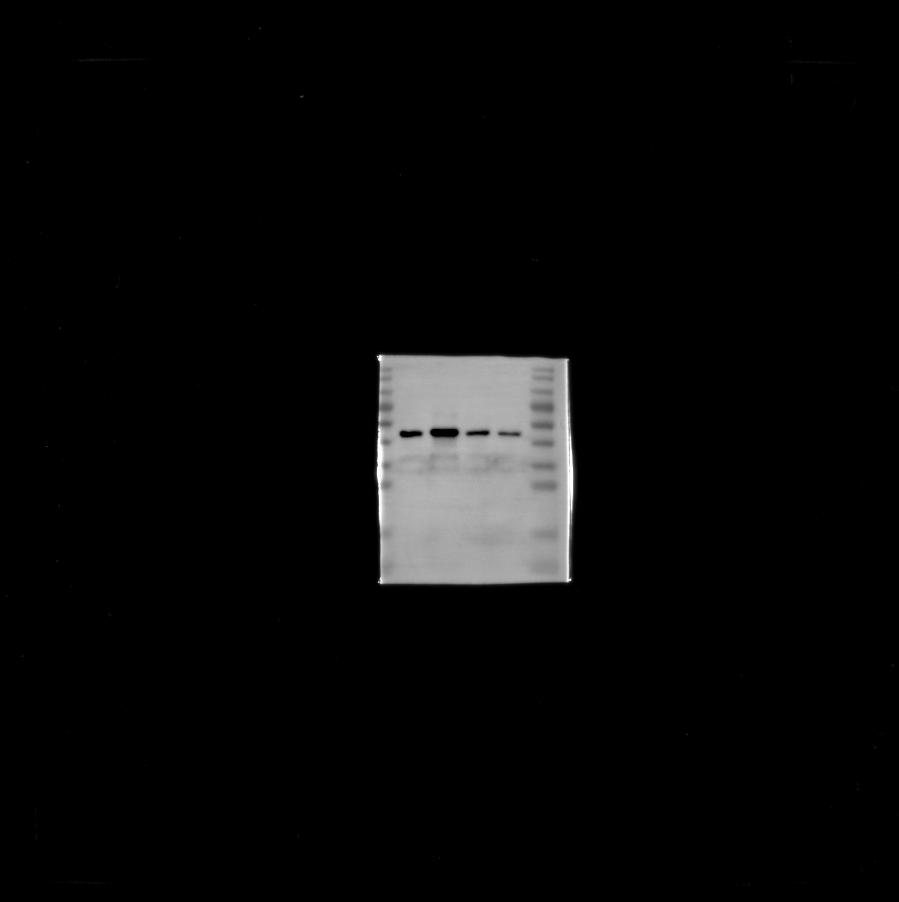
**

**55kda**

**43kda**

**aSMA**

**aSMA**

**aSMA**

**
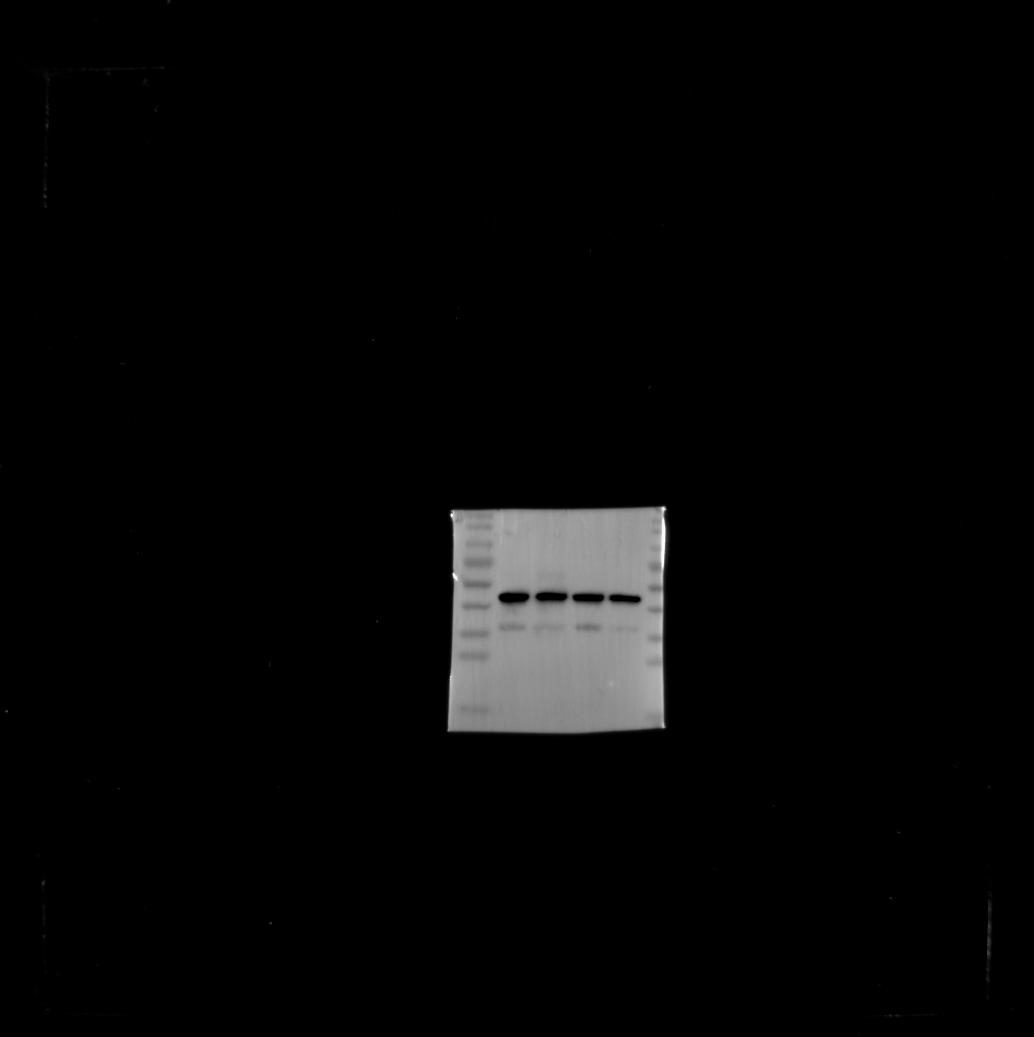

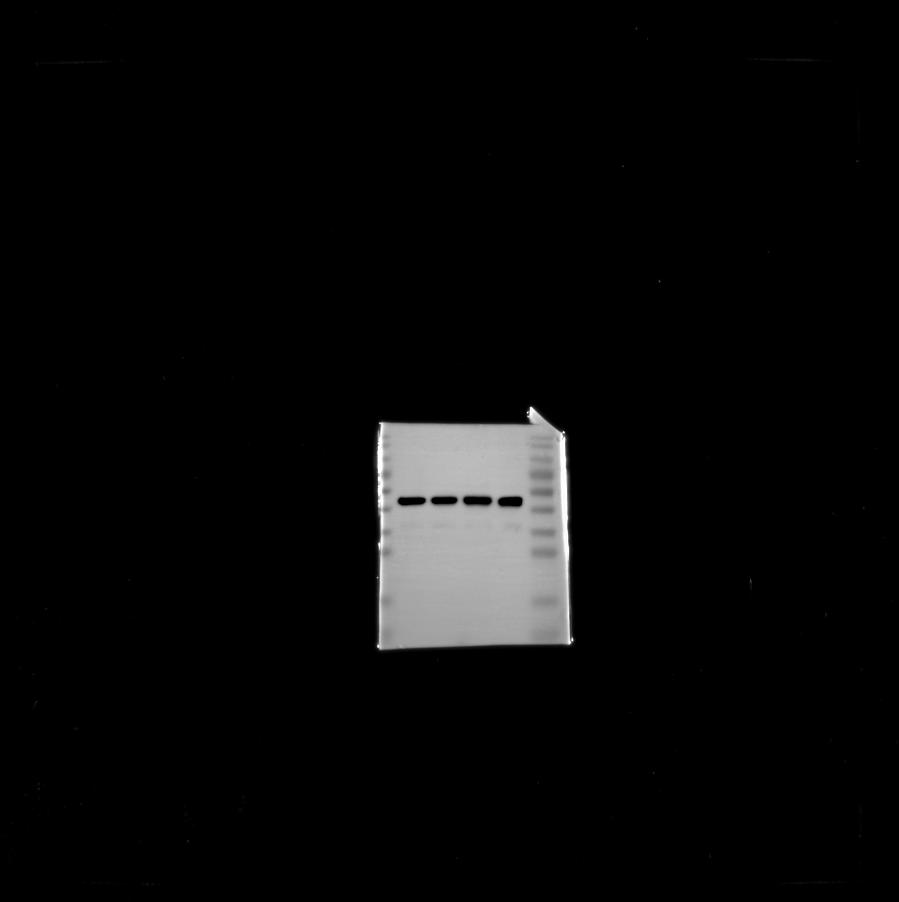

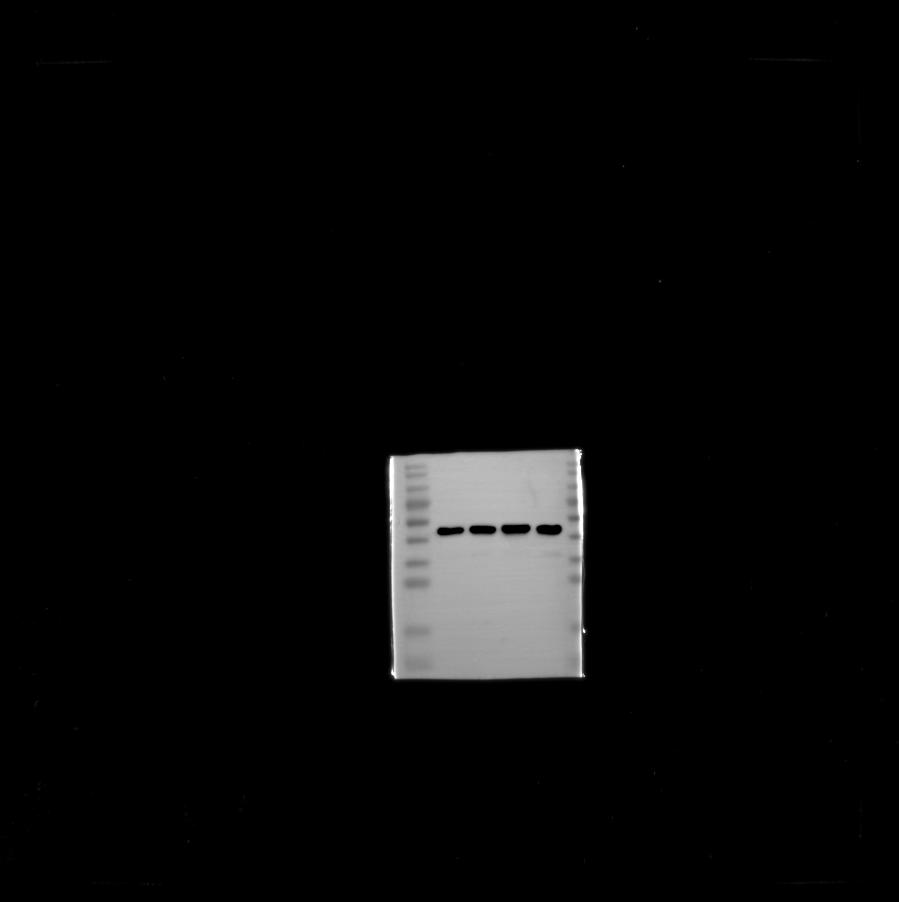
**

**55kda**

**43kda**

**β-actin**

**β-actin**

**β-actin**

**Uncropped Gels and Blots image(s) in Figure 5H**

**
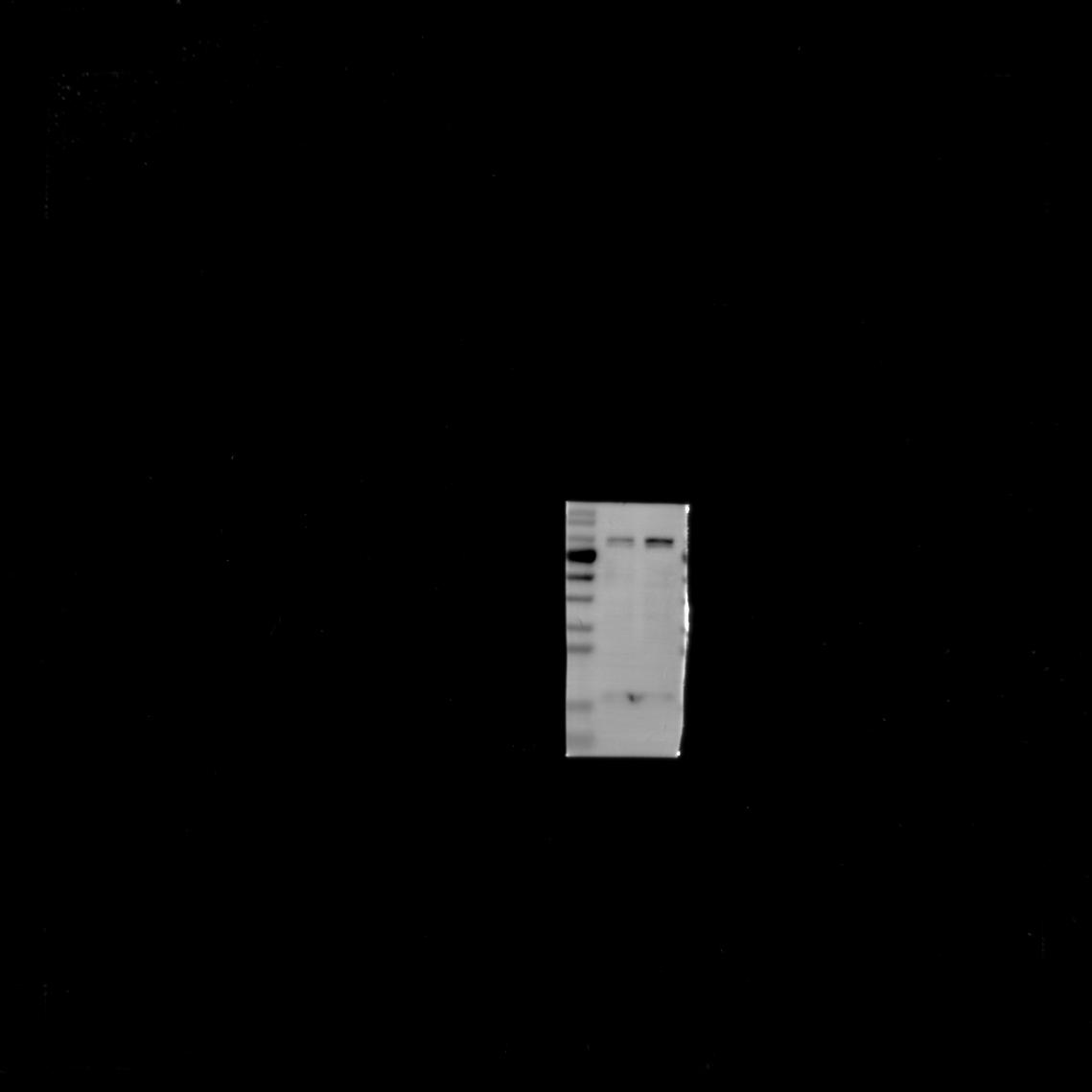

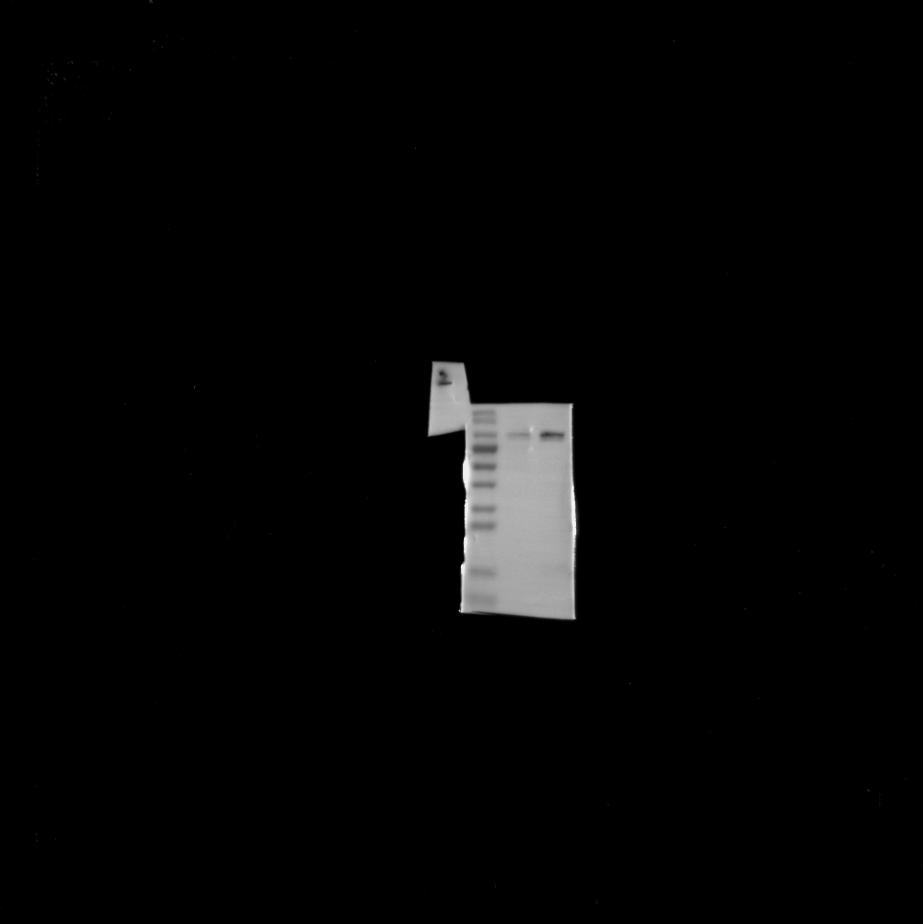
**

**
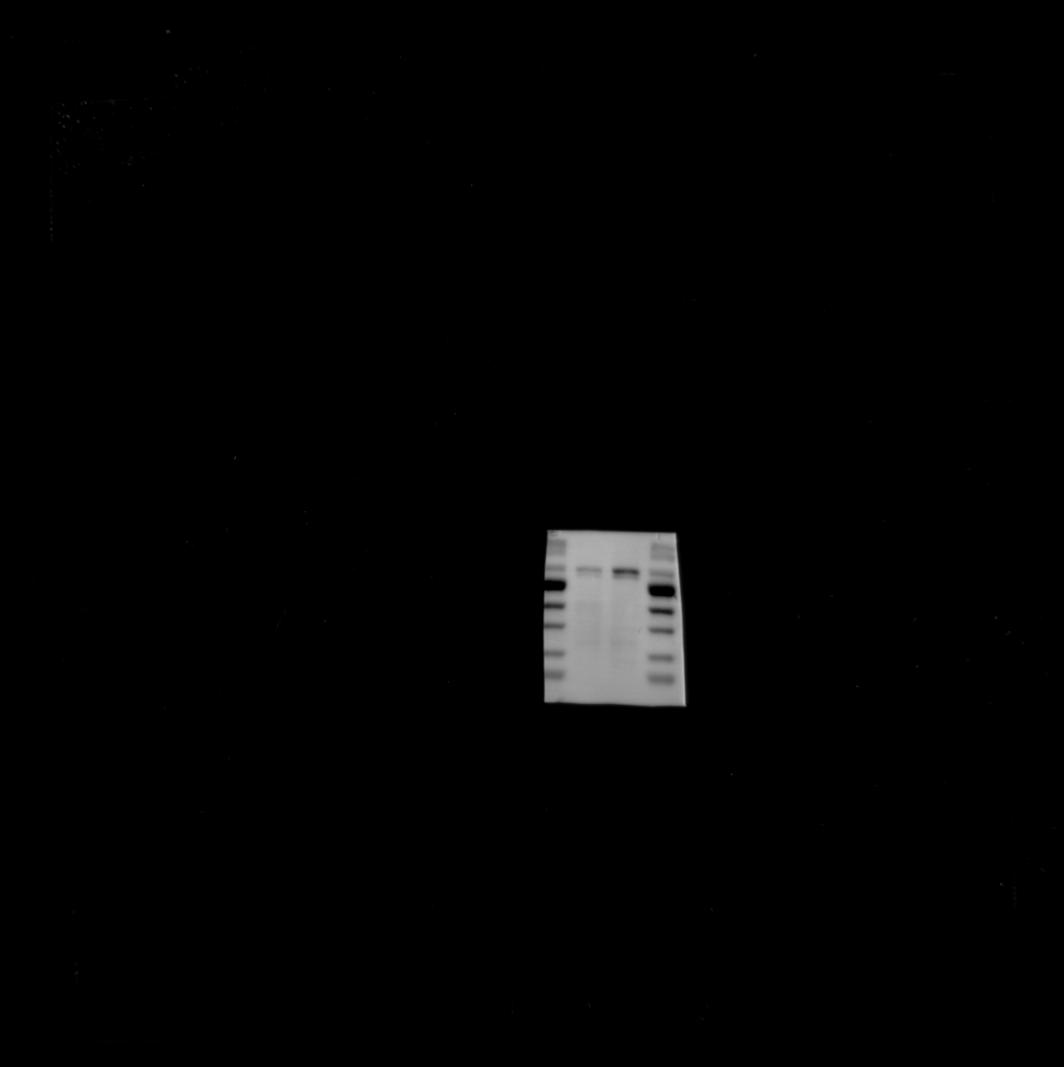
**

**95kda**

**PSTAT3**

**PSTAT3**

**PSTAT3**

**
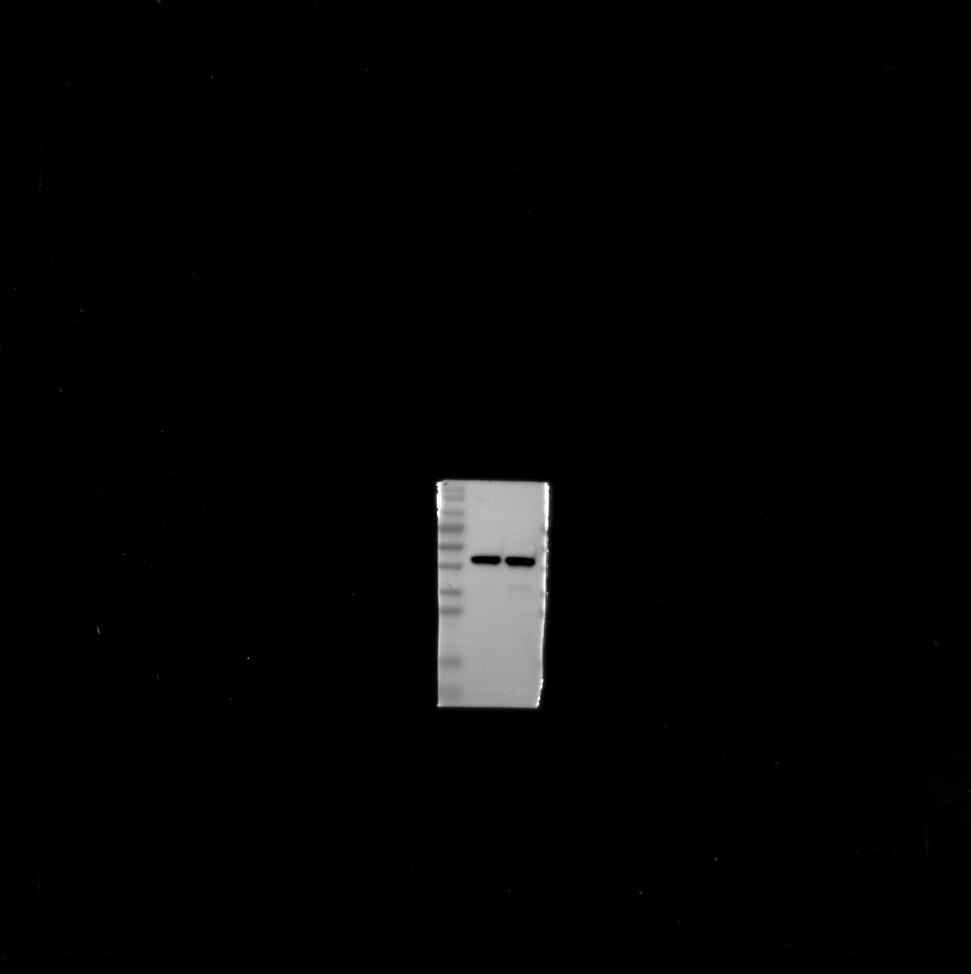
**

**75kda**

**
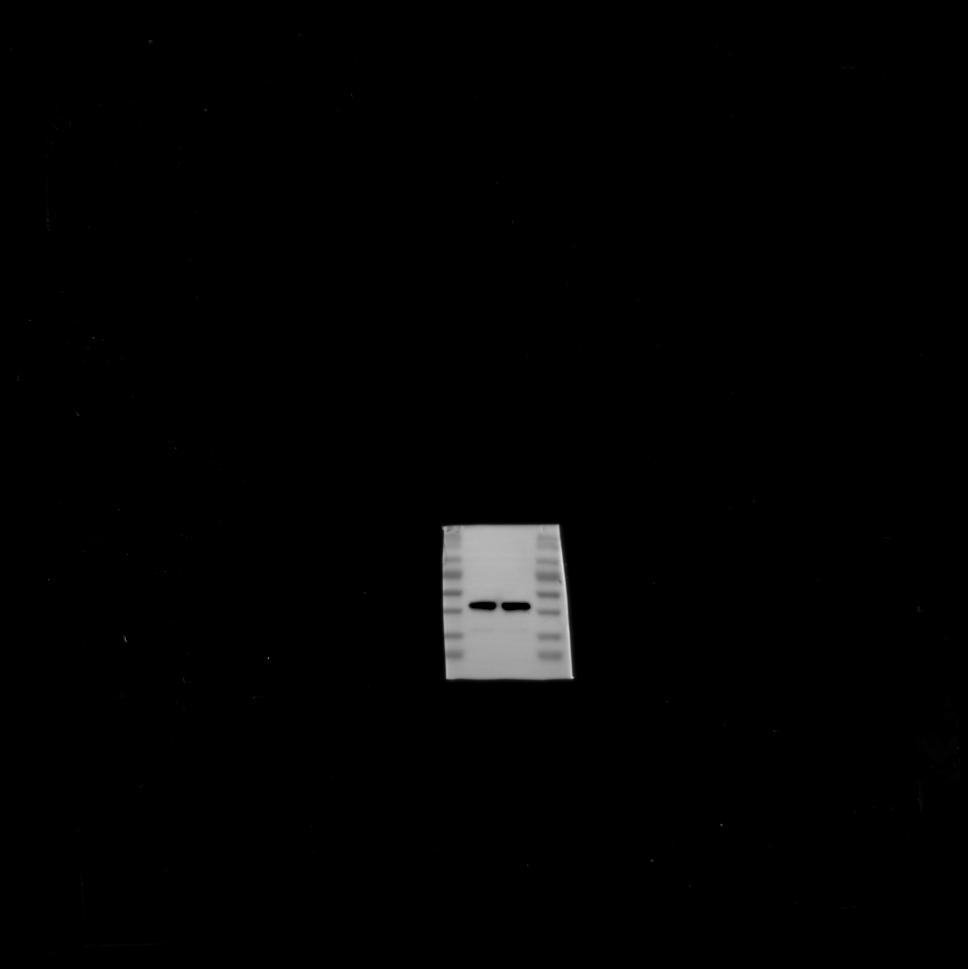

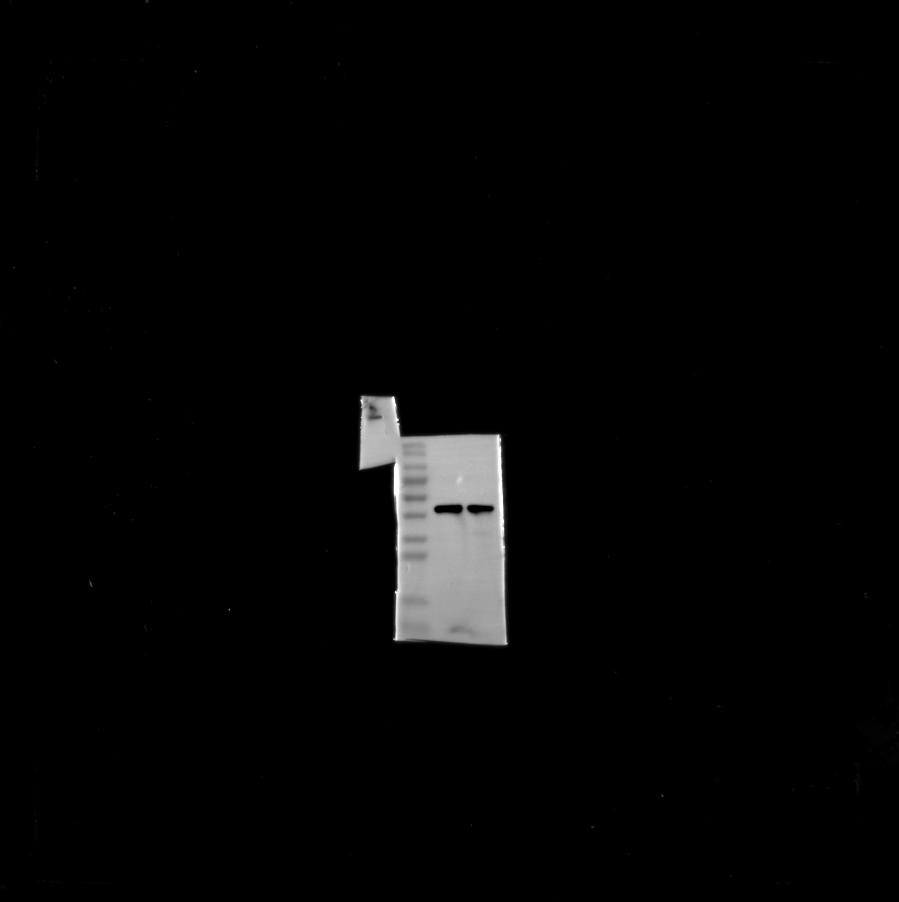
**

**55kda**

**β-actin**

**43kda**

**β-actin**

**β-actin**

**
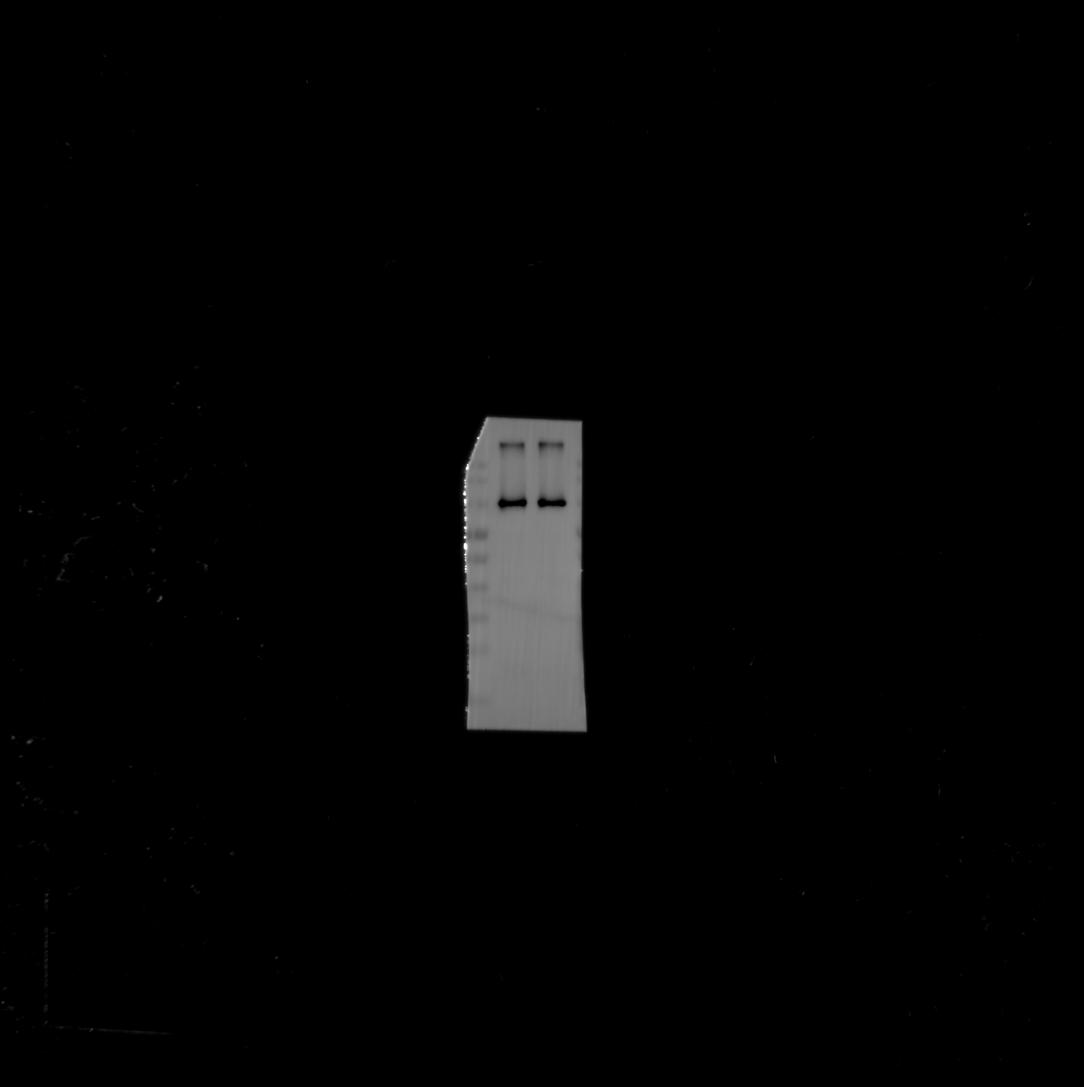

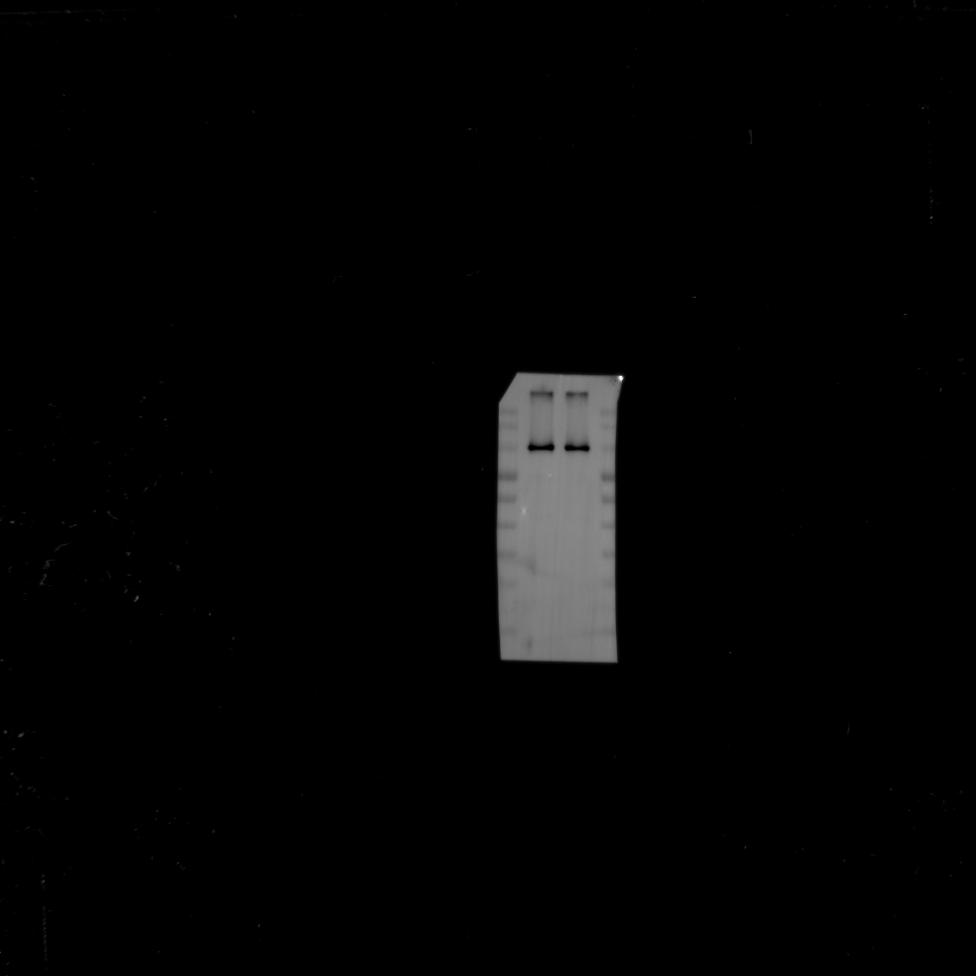

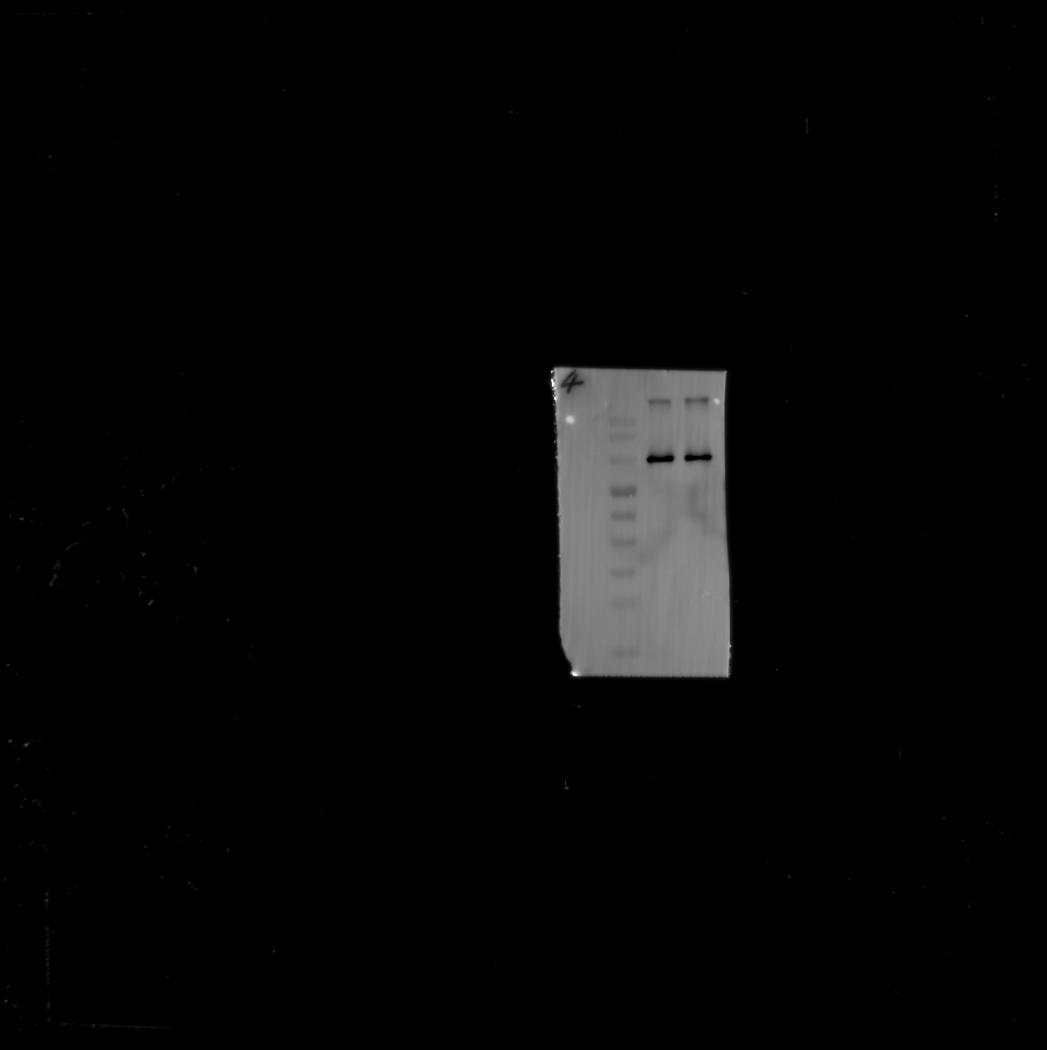
**

**130kda**

**95kda**

**STAT3**

**STAT3**

**75kda**

**STAT3**

**
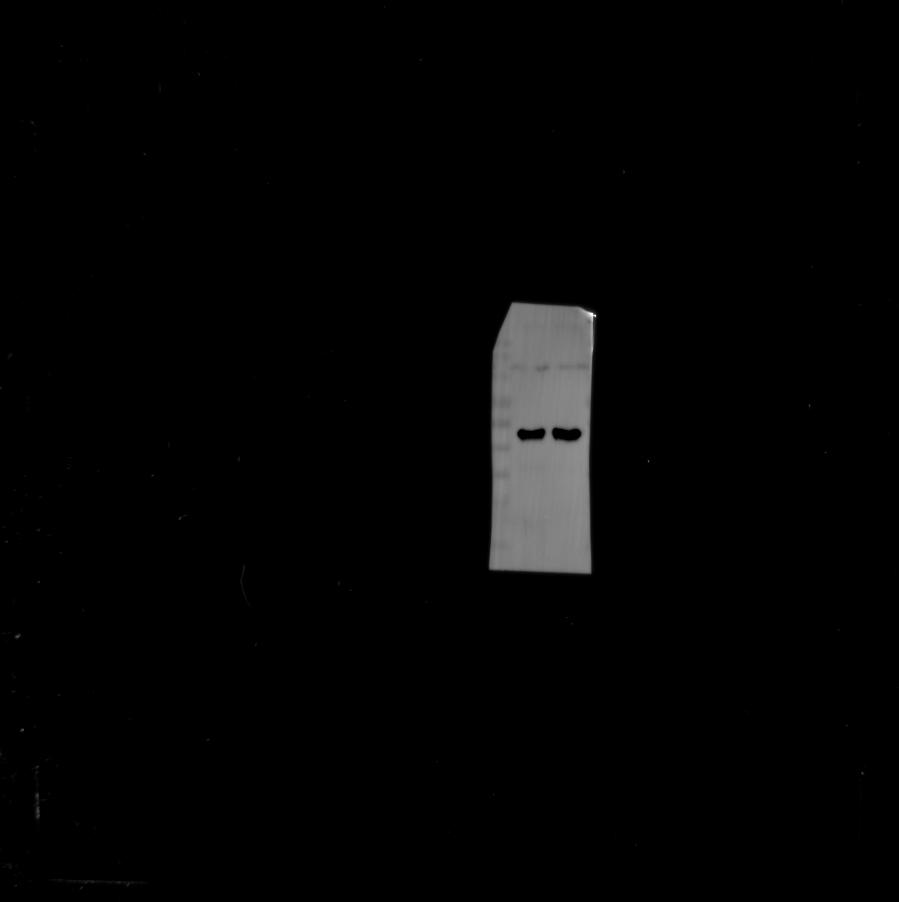
**

**
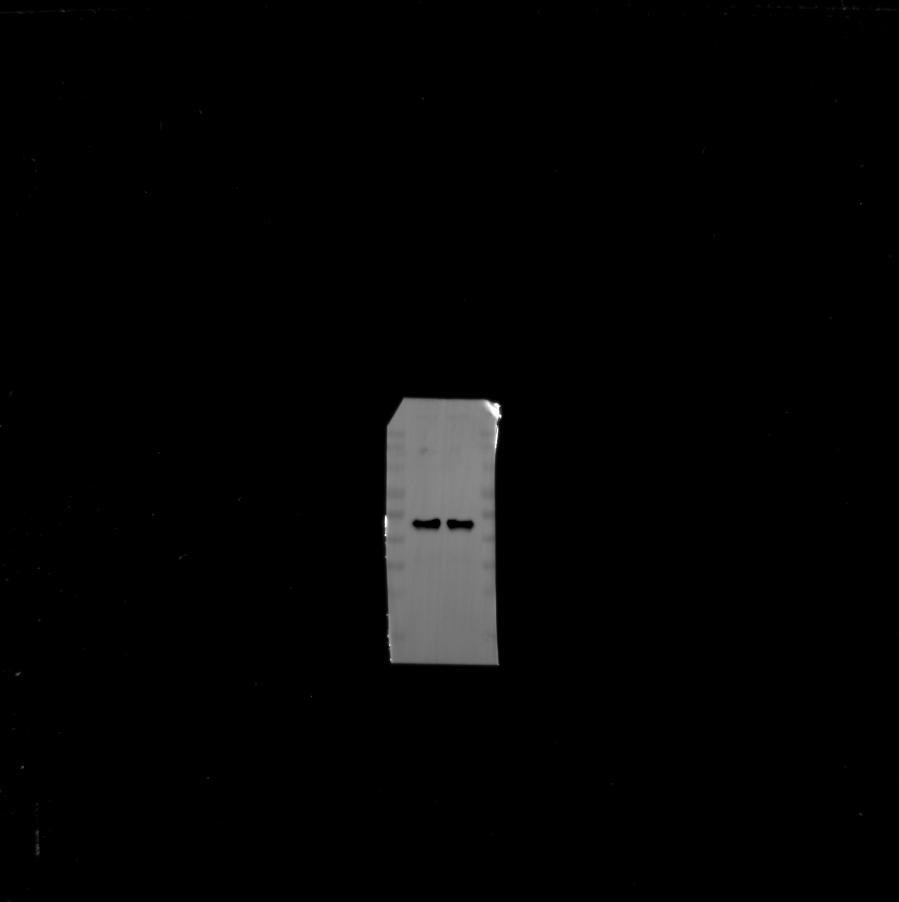

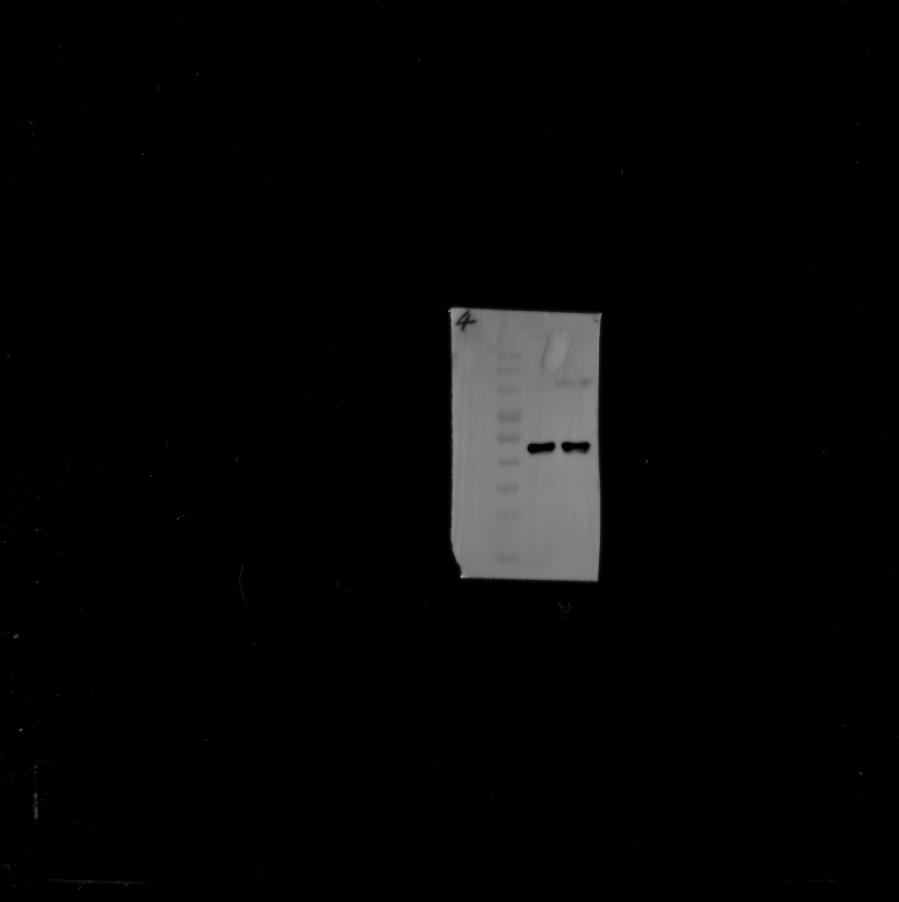
**

**55kda**

**43kda**

**β-actin**

**β-actin**

**β-actin**

**Uncropped Gels and Blots image(s) in
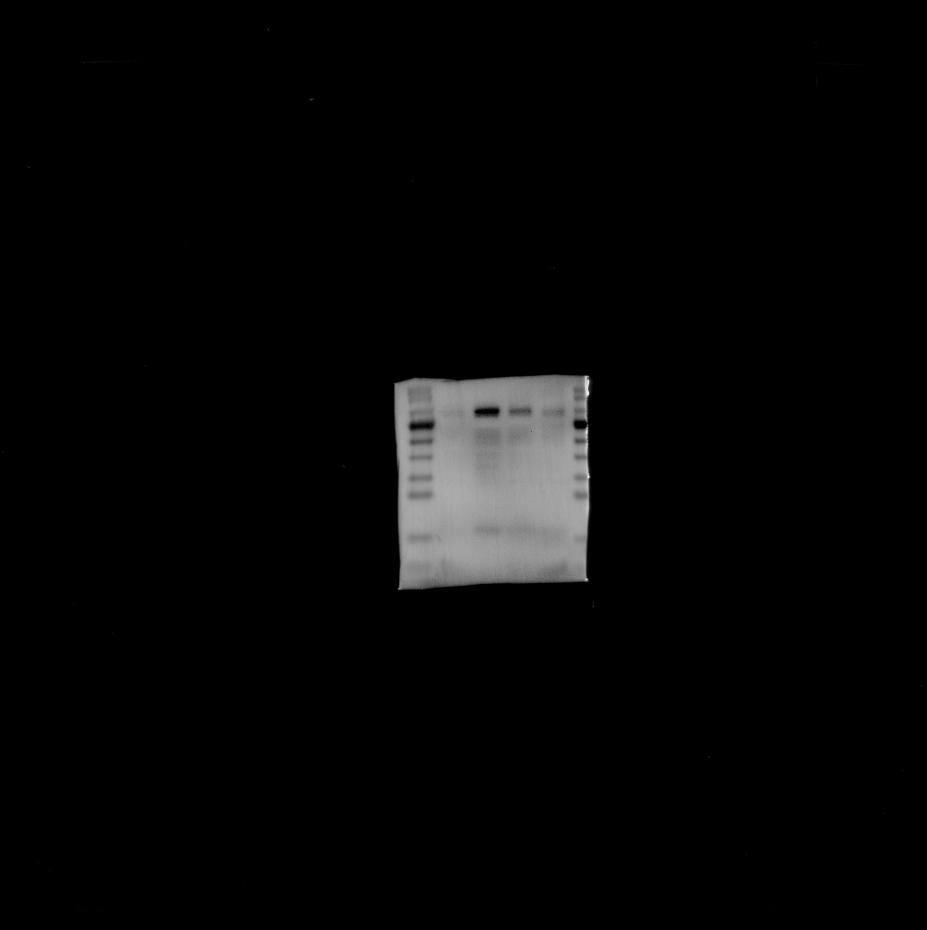
Figure 5I**

**
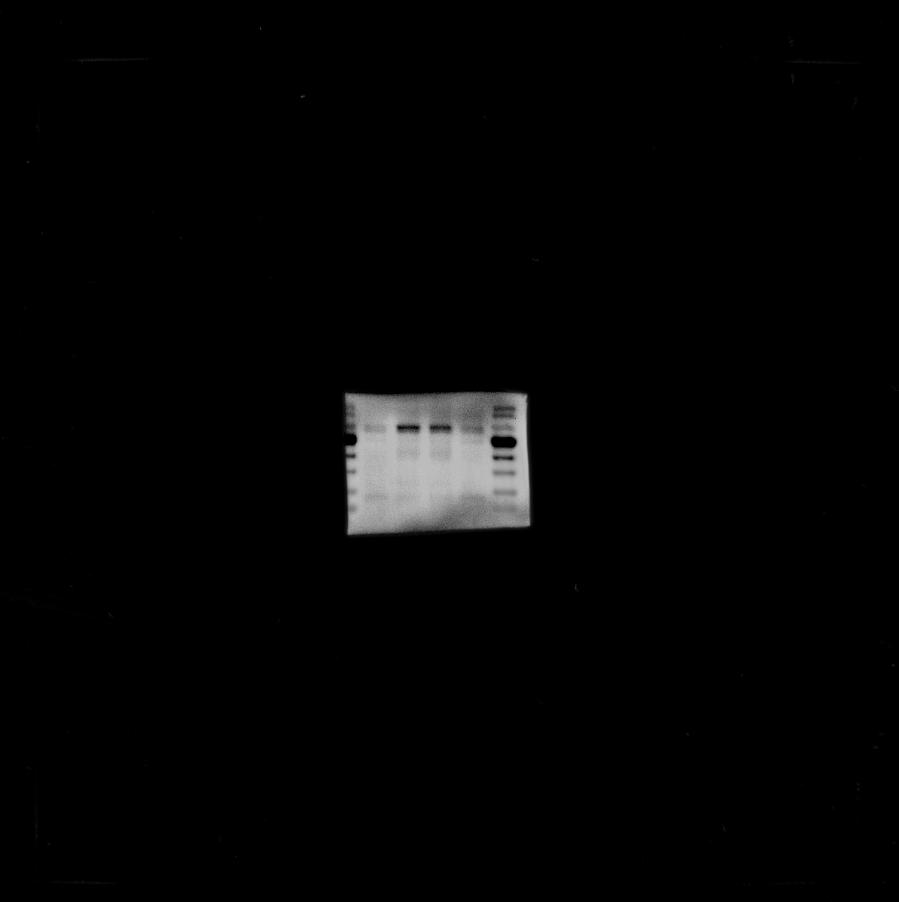

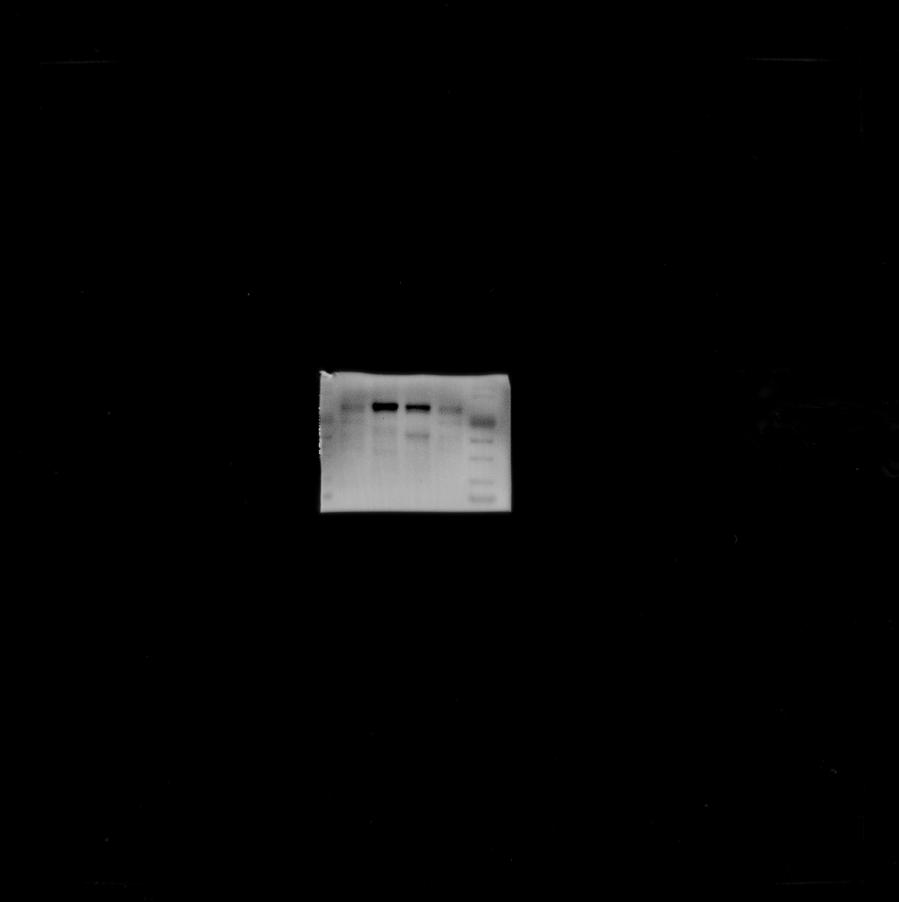
**

**95kda**

**PSTAT3**

**75kda**

**PSTAT3**

**PSTAT3**

**
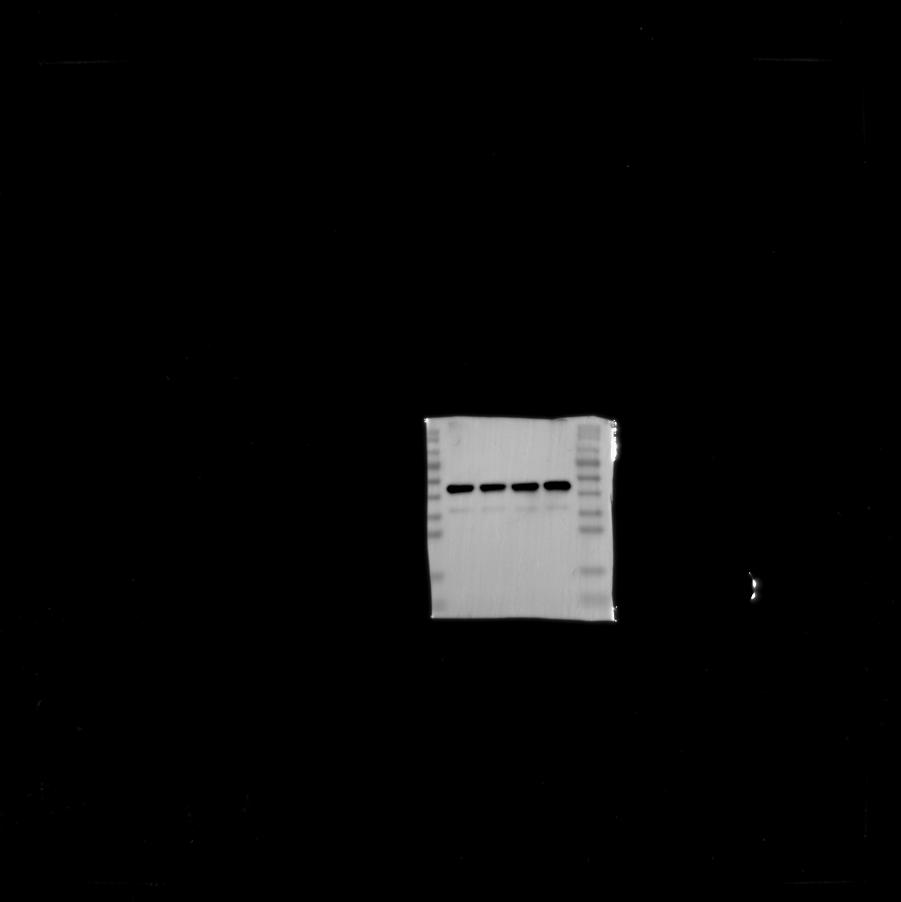
**

**
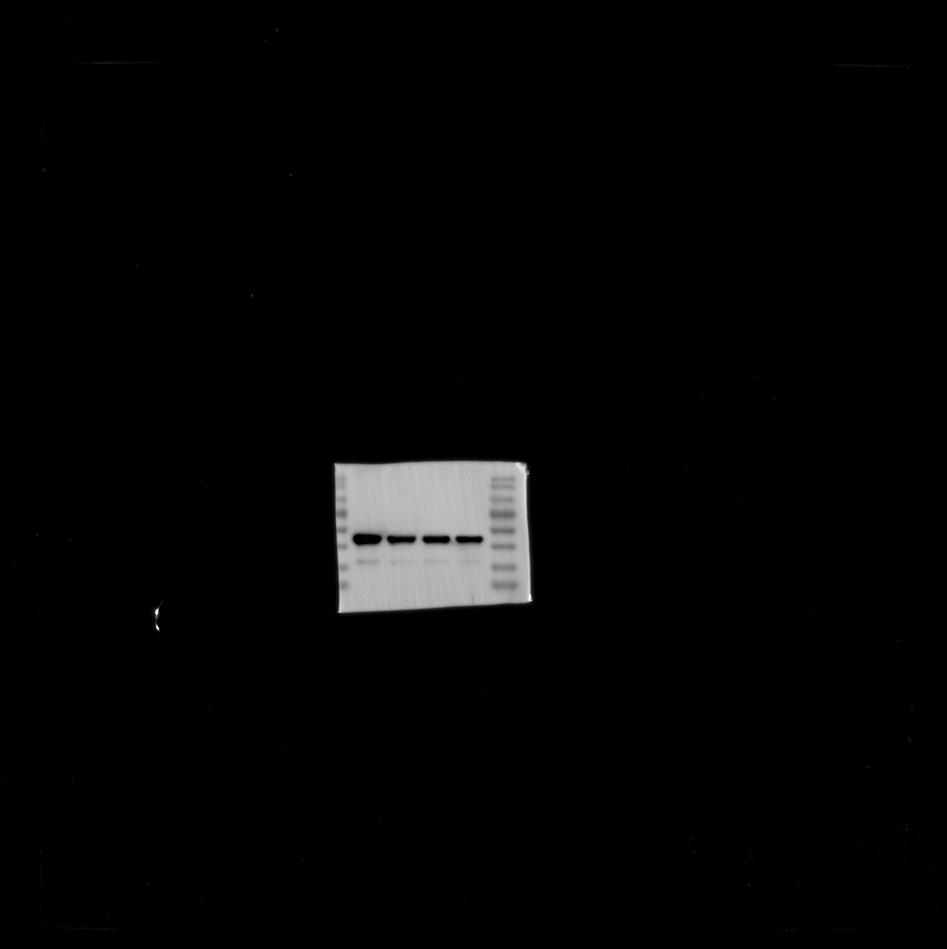

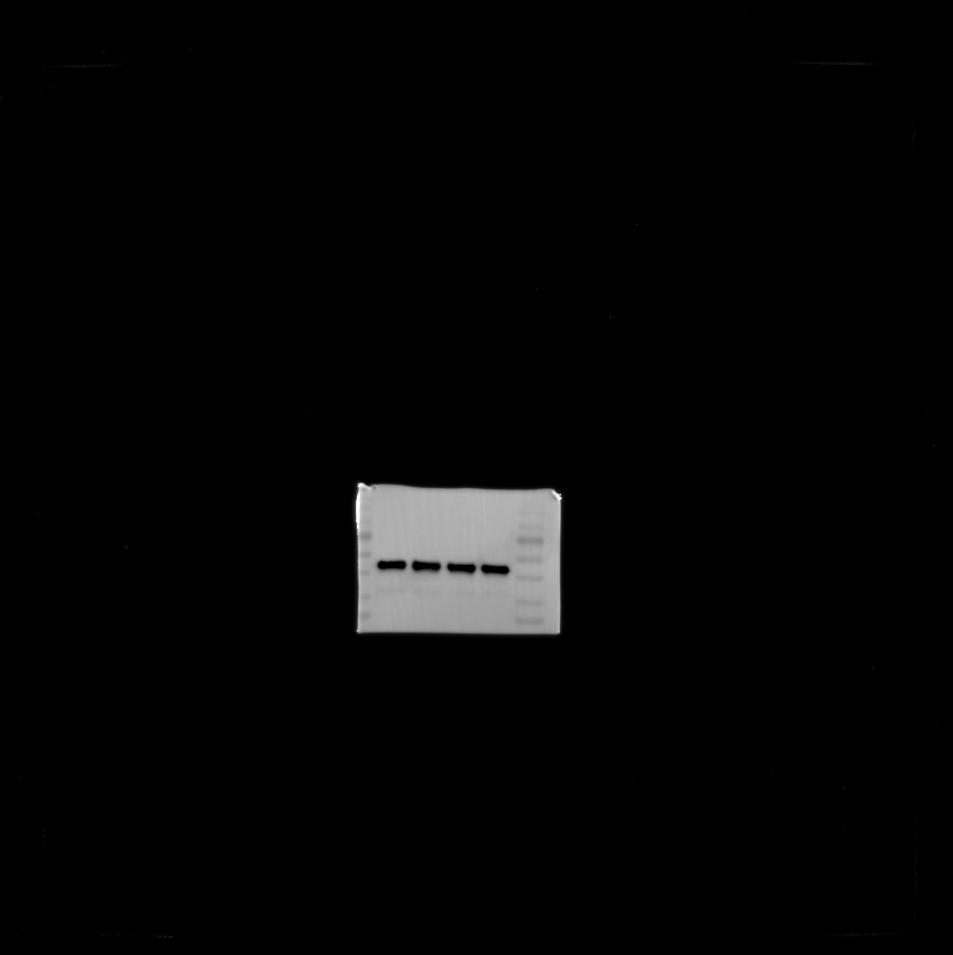
**

**55kda**

**β-actin**

**43kda**

**β-actin**

**β-actin**

**
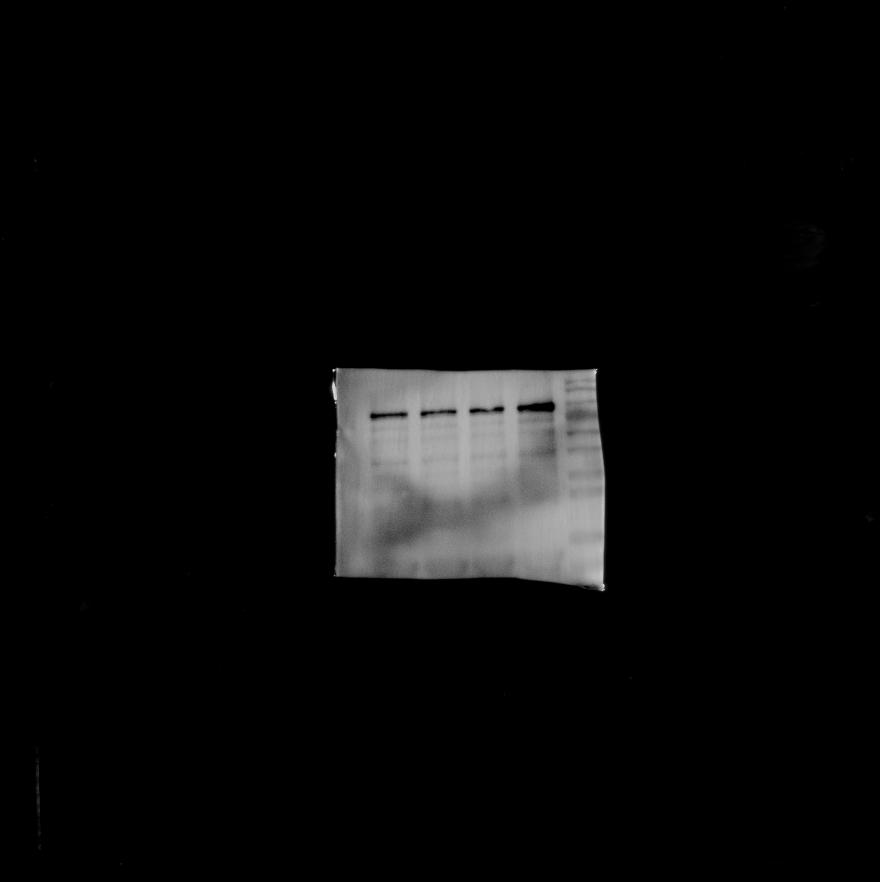
**

**
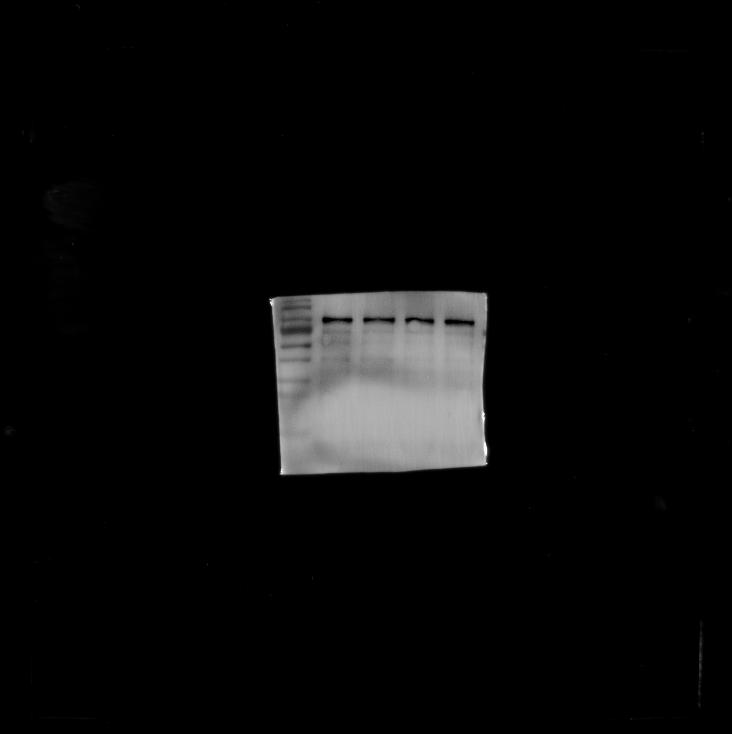

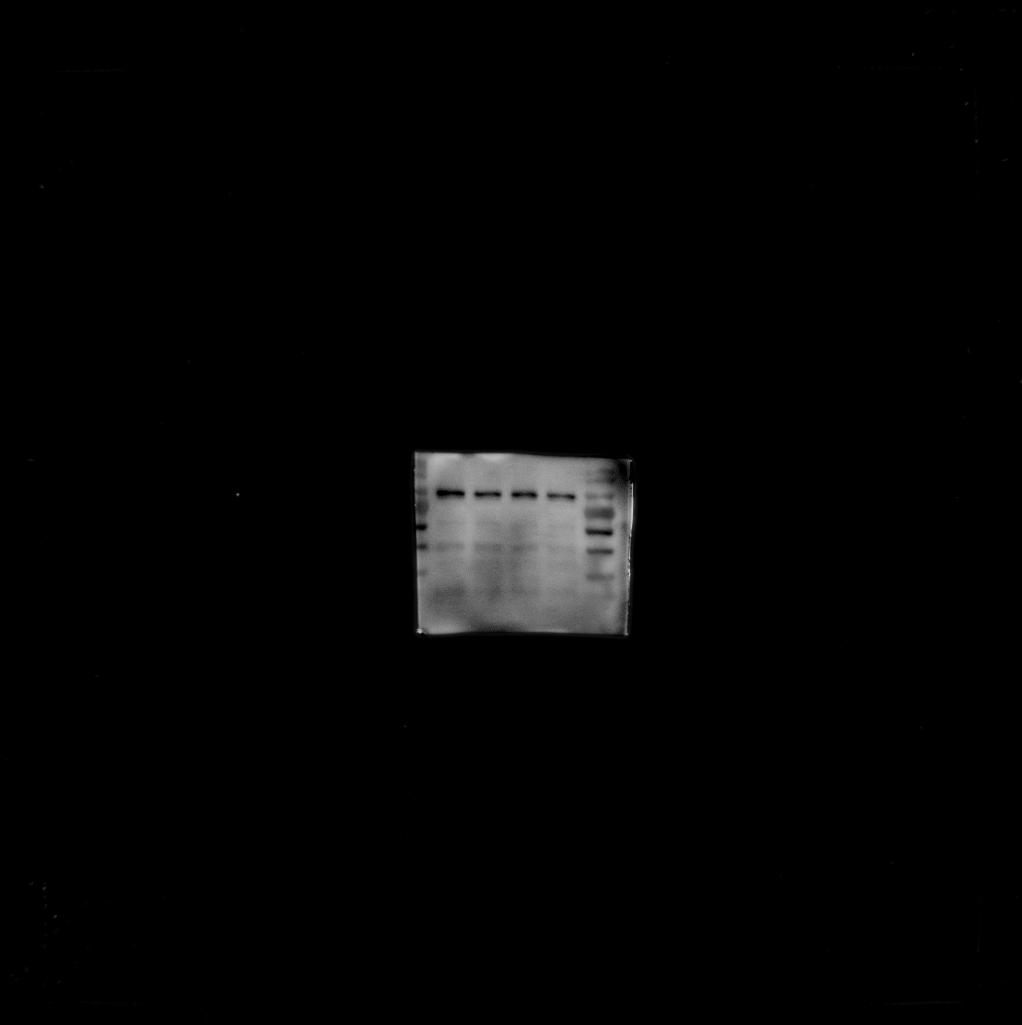
**

**95kda**

**STAT3**

**75kda**

**STAT3**

**STAT3**

**
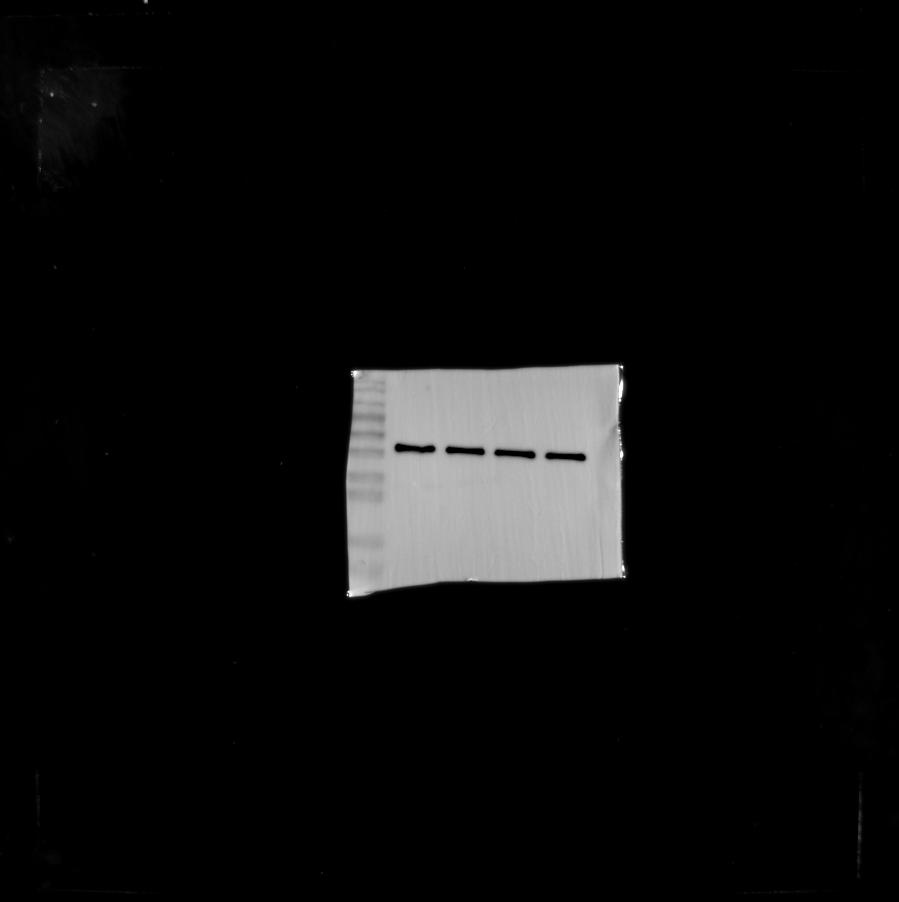
**

**
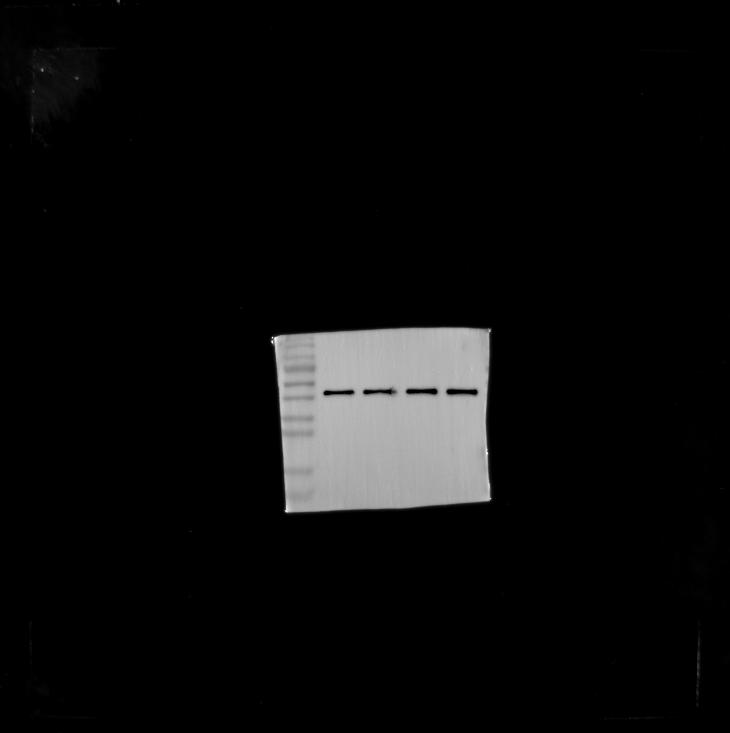

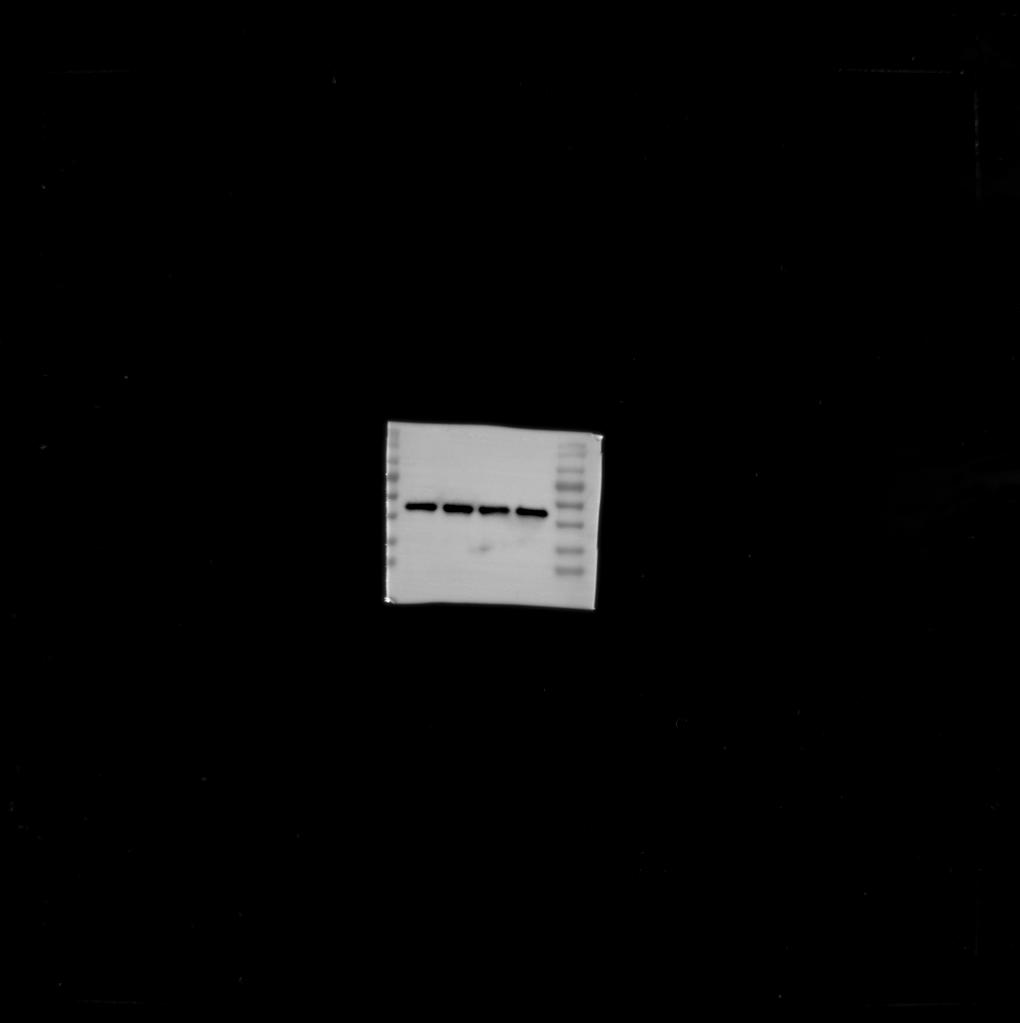
**

**55kda**

**43kda**

**β-actin**

**β-actin**

**β-actin**

**Table S1 Clinical Baseline Characteristics of Patients with Acute Pulmonary Thromboembolism**

| Indicator | APE-1 | APE-2 | APE-3 | APE-4 | APE-5 | APE-6 |
| --- | --- | --- | --- | --- | --- | --- |
| Gender | Male | Male | Female | Male | Female | Female |
| Age (years) | 60 | 35 | 66 | 60 | 49 | 54 |
| BMI (kg/m²) | 17.3 | 32.65 | 30.39 | 20.76 | 20.0 | 25.67 |
| Smoking history | Yes | No | No | No | No | No |
| Systolic pressure (mmHg) | 110 | 129 | 161 | 126 | 101 | 116 |
| Diastolic pressure (mmHg) | 71 | 86 | 79 | 72 | 62 | 75 |
| Heart rate (beats/min) | 77 | 82 | 80 | 89 | 89 | 87 |
| Respiratory rate (breaths/min) | 18 | 36 | 34 | 26 | 24 | 29 |
| SpO2 (%) | 85.5 | 73.29 | 79.14 | 77.85 | 59.7 | 76.45 |
| Oxygenation index (cmH₂O/mmHg) | 131.54 | 192.87 | 239.82 | 108.13 | 66.33 | 124.67 |
| Body temperature (°C) | 36.5 | 37.0 | 36.7 | 36.5 | 37.2 | 36.9 |
| Consciousness status | Impaired | Clear | Clear | Clear | Clear | Clear |
| History of VTE | - | - | Yes | Yes | Yes | Yes |
| Chronic heart or lung disease | - | - | Yes | - | - | - |
| Cancer or myeloproliferative disorder | - | - | - | Yes | - | - |
| Diabetes | - | - | - | - | - | - |
| Hypertension | - | - | Yes | Yes | - | - |
| Chronic liver disease | - | - | - | - | - | - |
| Renal insufficiency | - | - | - | - | - | - |
| Chronic inflammation/autoimmune disease | - | - | - | - | - | - |
| Recent surgery/trauma (past 30 days) | - | - | - | - | - | - |
| Recent immobilization (past 30 days) | - | - | - | - | - | - |
| Chronic obstructive pulmonary disease (COPD) | - | - | Yes | - | Yes | - |
| Hormone therapy | - | - | - | - | - | - |
| Pregnancy/lactation period | - | - | - | - | - | - |
| WBC count (×10⁹/L) | 7.7 | 8.11 | 4.82 | 7.1 | 7.31 | 7.97 |
| Neutrophil count (×10⁹/L) | 6.87 | 6.05 | 2.36 | 5.66 | 6.51 | 6.01 |
| Eosinophil count (×10⁹/L) | 0.59 | 1.48 | 1.96 | 0.85 | 0.43 | 1.23 |
| Monocyte count (×10⁹/L) | 0.17 | 0.54 | 0.3 | 0.44 | 0.29 | 0.25 |
| Hemoglobin (g/L) | 113 | 138 | 110 | 104 | 154 | 114 |
| Platelet count (×10⁹/L) | 266 | 210 | 243 | 227 | 130 | 287 |
| Total protein (g/L) | 76.3 | 73.1 | 77.3 | 73.8 | 75.1 | 76.2 |
| Albumin (g/L) | 30.5 | 36.2 | 36 | 33.9 | 34.9 | 31.6 |
| Total bilirubin (μmol/L) | 6.5 | 14.5 | 9.2 | 8.6 | 8.1 | 7.5 |
| Aspartate aminotransferase (U/L) | 19.5 | 141.1 | 23.3 | 23.5 | 2.8 | 4.8 |
| Alanine aminotransferase (U/L) | 26.4 | 45.6 | 17.5 | 16.6 | 12.8 | 17.9 |
| Uric acid (μmol/L) | 272.5 | 265.3 | 263.8 | 307.6 | 380 | 304.9 |
| Creatinine (μmol/L) | 62 | 69 | 46 | 116 | 63 | 67 |
| Blood glucose (mmol/L) | 6.6 | 4.6 | 5.3 | 6.7 | 8.3 | 6.2 |
| Urea nitrogen (mmol/L) | 6.83 | 4.67 | 6.62 | 7.28 | 11.17 | 7.58 |
| C-reactive protein (mg/L) | 94.18 | 25.81 | 6.05 | 48.32 | 100.54 | 56.85 |
| NT-proBNP (pg/mL) | 1322.9 | 186.9 | 161.6 | 2384.7 | 8894.9 | 2375.7 |
| hs-cTnI (ng/mL) | 0.0162 | 0.0112 | 0.0657 | 0.0193 | 0.0657 | 0.0156 |
| D-dimer (mg/L) | 2.25 | 6.66 | 3.66 | 10.37 | 3.17 | 3.25 |
| sPESI score | 1 | 1 | 2 | 1 | 2 | 1 |
| Overall risk level | Moderate to high | Moderate to high | Moderate to high | Moderate to high | Moderate to high | Moderate to high |
